# Stereo- and Enantioselective Addition of Organolithiums to 2-Oxazolinylazetidines as a Synthetic Route to 2-Acylazetidines

**DOI:** 10.3389/fchem.2019.00614

**Published:** 2019-09-10

**Authors:** Pantaleo Musci, Marco Colella, Flavio Fanelli, Angela Altomare, Luisa Pisano, Claudia Carlucci, Renzo Luisi, Leonardo Degennaro

**Affiliations:** ^1^Flow Chemistry and Microreactor Technology FLAME-Lab, Department of Pharmacy–Drug Sciences, University of Bari “A. Moro”, Bari, Italy; ^2^Crystallography Institute of the National Research Council (IC-CNR), Bari, Italy; ^3^Department of Chemistry and Pharmacy, University of Sassari, Sassari, Italy

**Keywords:** azetidine, lithiation, oxazoline, stereoselectivity, NMR calculations, oxazolidine

## Abstract

A new synthetic route to *N*-alkyl-2-acylazetidines was developed through a highly stereoselective addition of organolithiums to *N*-alkyl-2-oxazolinylazetidines followed by acidic hydrolysis of the resulting oxazolidine intermediates. This study revealed an unusual reactivity of the C=N bond of the oxazoline group when reacted with organolithiums in a non-polar solvent such as toluene. The observed reactivity has been explained considering the role of the nitrogen lone pair of the azetidine ring as well as of the oxazolinyl group in promoting a complexation of the organolithium, thus ending up with the addition to the C=N double bond. The high level of stereoselectivity in this addition is supported by DFT calculations and NMR investigations, and a model is proposed for the formation of the oxazolidine intermediates, that have been isolated and fully characterized. Upon acidic conditions, the oxazolidine moieties were readily converted into 2-acylazetidines. This synthetic approach has been applied for the preparation of highly enantioenriched 2-acylazetidines starting from chiral not racemic *N*-alkyl-2-oxazolinylazetidines.

## Introduction

**Figure 1:**
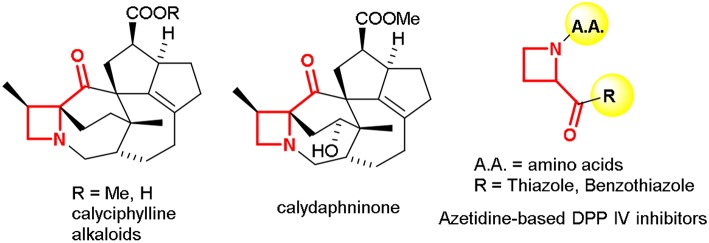
Examples of 2-acylazetidine motif in natural products and biologically active compounds.

The four-membered saturated heterocycle azetidine is a valuable scaffold exploited in several active research areas (Singh et al., 2008; Couty and Evano, 2009; Antermite et al., 2017). The strain and dynamic phenomena associated to the azetidine ring allows exploring new chemical space for organic synthesis and drug discovery purposes (Degennaro et al., 2014a,b; De Ceglie et al., 2011). Recently, different approaches have been reported for the synthesis of azetidine derivatives for targeting lead compounds and bioisosteres for drug discovery (Couty et al., 2004; Ferraris et al., 2007; Pérez-Faginas et al., 2011). Despite the great interest for this small-sized heterocycle, some structural motifs, such as 2-ketoazetidines, seems to be poorly investigated. Biologically active compounds, incorporating the 2-acylazetidine moiety, include natural products such as alkaloids found in the genus *Daphniphyllum* (Kobayashi and Kubota, 2009) as well as 2-aroylazetidines known as potent inhibitors of dipeptidyl peptidase IV (DPP-IV) (Ferraris et al., 2004), a proline-specific serine protease used as target in several therapeutic areas such as diabetes (Weber, 2004) pain (Ronai et al., 1999), and cognition enhancement (During et al., 2003; Figure 1).

**Scheme 1:**
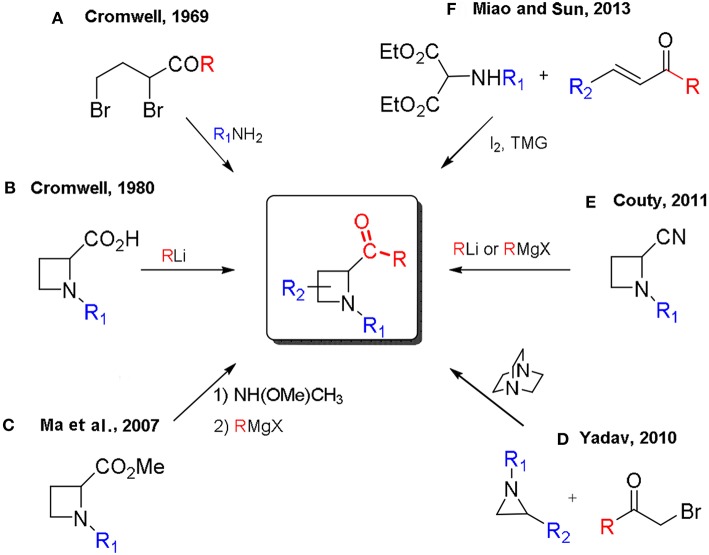
Reported synthesis of 2-acylazetidines.

Methods for the preparation of 2-acylazetidines could be traced back to 1969 with the first synthesis reported by Cromwell, by cyclization of 2,4-dibromoketones with primary amines (Scheme 1, A) or the addition of organolithiums to azetidines carboxylic acids (Rodebaugh and Cromwell, 1969, 1971; Kulkarni and Cromwell, 1977; Arnould et al., 1980; Scheme 1, B). Selected examples, for the stereoselective synthesis of 2-acylazetidines, include the nucleophilic addition of aryllithiums (Couty and Prim, 2002) and Grignard reagents (Couty et al., 2011) to optically active 2-cyanoazetidines (Scheme 1, E) or the addition of Grignard reagents to enantiopure Weinreb amides prepared from methyl 1-phenylethylazetidine-2-carboxylates (Ma et al., 2007; Scheme 1, C). Recently, the synthesis of 2-acylazetidines, by a one-pot tetramethylguanidine/I_2_-mediated formal [2+2] cycloaddition reaction of α-amidomalonate with enones, has been reported by Miao and Sun (Miao et al., 2013; Scheme 1, F). Yadav reported the ring expansion of aziridines, employing phenacyl bromide derivatives via *in situ* generated ammonium ylides in a silica gel-water reaction medium (Garima et al., 2010; Scheme 1, D).

**Scheme 2:**
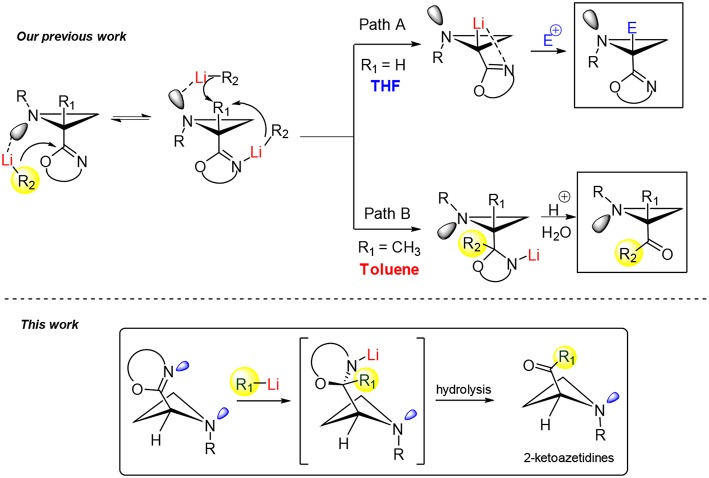
Example of reactivity controlled by dynamics in small nitrogenated heterocycles.

In 2011, our group reported an intriguing approach for the synthesis of 2-ketoaziridines exploiting an unprecedented reactivity profile of chiral 2-oxazolinylaziridines when subjected to reaction with organolithiums (Degennaro et al., 2011). This work focused on a regioselective α-lithiation of optically active *N*-phenylethyl-2-oxazolinylaziridines by using a strong base such as *n*-buthyllithium (*n*-BuLi) in a coordinating solvent (THF) at −78°C within 1 h. The resulting lithiated species proved to be chemically and configurationally stables, under the experimental conditions, and were trapped with electrophiles in highly stereoselective manner (Scheme 2, path A). In particular, this study demonstrated the presence of a mixture of two equilibrating invertomers for *N*-alkyl-2-oxazolinylaziridines as a result of the nitrogen inversion (Capriati et al., 2002; Luisi et al., 2005; Scheme 2). It was demonstrated by NMR and DFT calculations, that the dynamics at nitrogen was dependent on the nature of the substituent at the C2 of the azetidine ring. A dynamic model was proposed to account for a diverse and unexpected reactivity observed in a non-polar solvent such as toluene. In fact, as a consequence of a competing complexation of the organolithium reagent with the lone pair of the aziridine nitrogen led to an unusual attack to the C = N bond of the oxazoline ring with consequent formation of an oxazolidine intermediate, useful precursor of the corresponding 2-acylaziridine (Scheme 2, path B). Better results in term of yield and stereoselectivity were obtained by reacting C2-substituted oxazolinylaziridines with organolithiums in toluene and at higher temperature (0°C). Therefore, this model suggests that controlling the nitrogen dynamics, in these systems, could be possible to address the reactivity providing both α-substituted aziridines and 2-ketoaziridines.

Inspired by these preliminary results on 2-oxazolinylaziridines, highlighting the role of nitrogen dynamics on the reactivity of this three-membered heterocycles, we were keen to build on this ground a stereoselective synthesis of 2-ketoazetidines, likely starting from the corresponding 2-oxazolinyl azetidines (Scheme 2). We are glad to report herein the results obtained in this investigation.

## Results and Discussion

### Substrates Synthesis

**Scheme 3:**
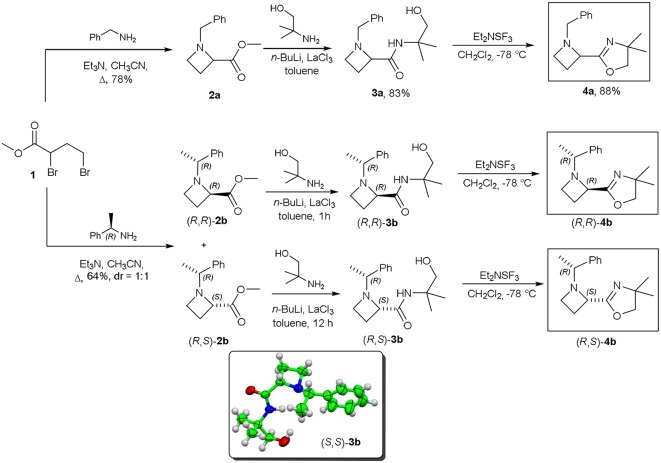
Substrates synthesis.

The first step for the synthesis of 2-oxazolinylazetidines **4a** (*R, R*)**-4b** and (*R, S*)**-4b**, reported in Scheme 3, involved the reaction of methyl 2,4-dibromobutanoate **1**, commercially available, with a suitable primary amine. When ester **1** was reacted with benzylamine, azetidinylester **2a** was obtained in high yield, and, by subsequent treatment with 2-amino-2-methylpropanol, *n*-hexyllithium, in presence of catalytic amount of LaCl_3_ afforded hydroxamide **3a** (Jang et al., 2015; Scheme 3). The last step consisted in an intramolecular cyclization of hydroxamide **3a** mediated by diethylaminosulfur trifluoride at low temperature, that provided the desired oxazoline **4a**. A similar protocol was applied to the preparation of 2-methylcarboxylates (*R, R*)**-2b**, and (*R, S*)**-2b**, by using (*R*)-1-phenylethylamine as chiral not racemic amine, to yield orthogonally protected and chromatographically separable diastereomeric mixture of esters in 64% yield in 1:1 ratio. Hydroxamides (*R, R*)**-3b** and (*R, S*)**-3b** where separately synthesized as previously described for amide **3a** and, in the case of amide (*S, S*)**-3b**, prepared from (*S*)-1-phenylethylamine, the structure has been solved by X-Ray analysis, confirming the chemical structure and the absolute stereochemistry of the compound (see Supplementary Material). Finally, the cyclization step yielded 2-oxazolinylazetidines (*R, R*)**-4b** and (*R, S*)**-4b**, as single diastereoisomers, each with excellent enantiomeric ratio (er = 98:2).

### Optimization Study and Scope

According to a recent report by Couty and coworkers, the addition of organolithiums or Grignard reagents to azetidine carboxylic esters (*R, S*)-**2b**, occurred with low chemoselectivity, providing the corresponding tertiary alcohol as the main product (Couty et al., 2011). This prompted us to consider the approach reported in Scheme 2 as an alternative route to 2-acylazetidines. We started our investigation considering the reaction of azetidine **4a** with *n-*BuLi in toluene (Table 1). Pleasingly, oxazolidine **5a** was the sole product observed in these experiments with no evidence for the α-deprotonation of the azetidine ring.

**Table 1:** Reaction of azetidine **4a** with *n*-BuLi.

**No**.	**Organolithium equivalents**	**Temperature (^**°**^C)**	**Diastereomeric ratio (d.r.)^b^**	**Yield^a^**
1	1.1	20	82:18	30
2	1.1	0	82:18	50
3	1.1	−40	88:12	65
4	1.1	−78	98:2	87
5	2	0	80:20	41
6 ^c^	4	0	-	-

a, b Calculated by ^1^H NMR or GC analysis on the crude mixture;

c *Complex mixture formed*.

**Scheme 4:**
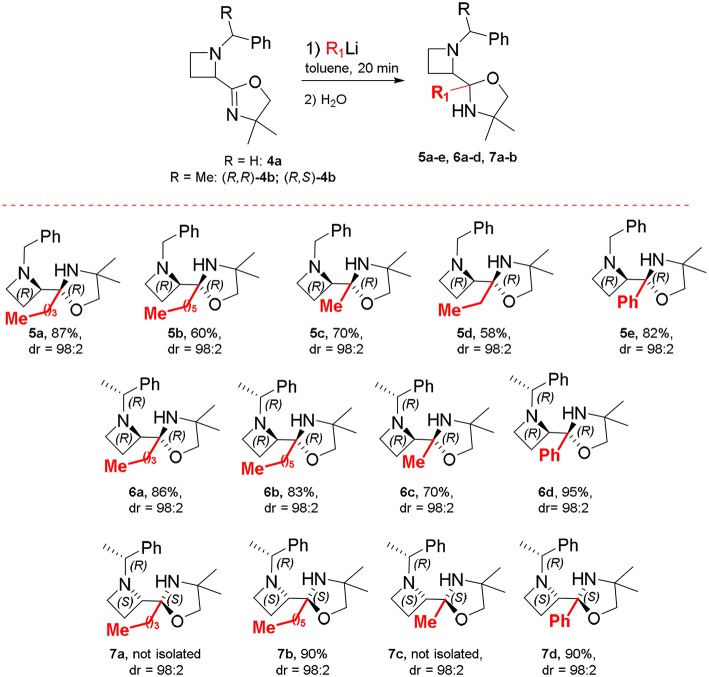
Oxazolidine synthesis.

By adding *n*-BuLi at room temperature, within 20 min, azetidine **5a** was obtained in 30% yield, after aqueous work up, as a 82:18 mixture of diastereomers (Table 1, No. 1). With the aim to improve both yield and diastereoselectivity, the reaction was conducted at lower temperature. To our delight, running the reaction at −78°C furnished the product **5a** in high yield and as single diastereoisomer (Table 1, No. 4). The use of an excess *n*-BuLi, considerably lowered the yields, giving also a complex mixture of products (Table 1, No. 5-6). We considered the conditions in No. 4 as optimal for the examination of the reaction scope. Under optimized conditions, the nucleophilic addition of *n*-hexylLi, MeLi and EtLi proceeded in highly stereoselective manner affording compounds **5b-d** as single diastereoisomer with satisfactory yields (Scheme 4). By using PhLi as nucleophile, the reaction required longer times (3 h) and product **5e** was obtained with 82% yield and 98:2 dr. Surprisingly, the reaction with 2-thienyllithium did not afford any product, even keeping the reaction up to 6 h at −78 and 0°C, presumably as a consequence of the presence of ethereal solvents, namely THF, in 2-thienyllithium solution. Based on these results, the same protocol (i.e., toluene, −78°C), was tested using optically active oxazolinylazetidine (*R, R*)-**4b**. Unexpectedly, the addition reaction was not observed at −78°C, but full conversion was obtained running the reaction at 0°C in 20 min, obtaining oxazolidine **6a** in 86% yield as a single diastereomer (dr > 98:2) and highly enantioenriched (er = 98:2). The modified conditions were also employed for preparing in good yield and diastereoselectivity oxazolidines **6b-d**, and **7a-d** starting from oxazolinylazetidine (*R, R*)*-***4b** and (*R, S*)*-***4b**, respectively (Scheme 4).

### Proposed Model for the Stereoselective Addition

**Figure 2:**
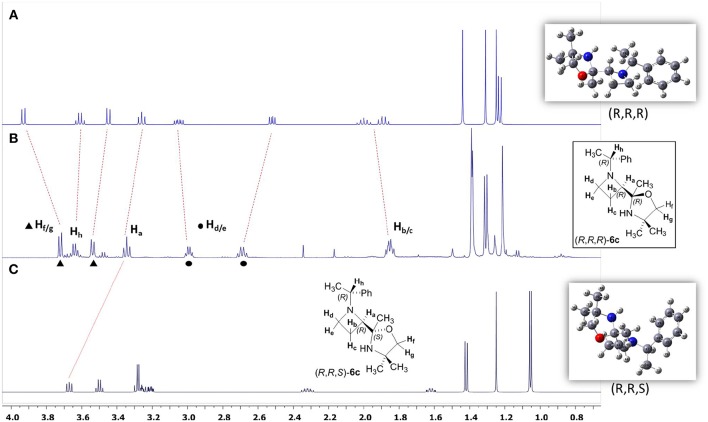
**(A)** Calculated spectrum of (*R,R,R*)-**6c**; **(B)** Real spectrum of (*R,R,R*)-**6c**; **(C)** Calculated spectrum of (*R,R,S*)-**6c**.

The high stereoselectivity observed for the C = N addition prompted us to consider the stereochemistry at the quaternary stereogenic center of the oxazolidine ring. All attempts to get suitable crystals for X-ray analysis were unsuccessful, and NOESY experiments were inconclusive. Based on our previous experience on the stereochemical assignments in three and four-membered heterocycles by using DFT calculations and NMR predictions, we decided to pursue this approach for solving this stereochemical puzzle (Azzena et al., 2018). The diastereoiomers (*R, R, R*)-**6c** and (*R, R, S*)-**6c** were considered. A relaxed potential energy surface (PES) scan of the dihedral angles C_43_N_16_C_17_C_19_ and O_2_C_3_C_11_C_12_, which define the relative position of the two substituents (oxazolidine ring and benzyl group) on the azetidinyl ring, was performed. All other parameters were allowed to vary freely during the PES scan; the single point energies were calculated at DFT/B3LYP/3-21G level in vacuo. After conformational minimization, the lowest energy conformers of the two diastereoisomers was subjected to fully unconstrained geometry optimization at SMD/DFT/B3LYP/6-311++G(d,p) level, followed by vibrational analysis. The free energy values provided by the vibrational analysis calculations indicated that diastereoisomer (*R, R, R*)-**6c** was 3.8 Kcal/mol more stable with respect to (*R, R, S*)-**6c**. At the end, optimized structures were used for prediction of nuclear shieldings using the gauge independent atomic orbital (GIAO) approach. GIAO NMR calculations were performed at SMD(CHCl_3_)/DFT/ MPW1PW91/gen level and the shielding tensors (σ_calc_) scaled to obtain the predicted chemical shifts (δscal, see Supplementary Material). The NMR chemical shifts (δ) were calculated as the differences of isotropic shielding constants (σ) with respect to the TMS (tetramethylsilane) reference, calculated at the same level of theory. The pcS-2 basis set, specifically designed for NMR shielding constant calculations (Jensen, 2008), was used for H and C atoms and 6-311++G(d,p) for N and O atoms. For indirect spin-spin coupling constants *J*_HH_ calculation, we selected the SMD/DFT/B3LYP/6-311++G(d,p) level of theory, a good compromise between accuracy and computational cost. Indeed in our earlier studies, we noticed the performance of this method in predicting proton and carbon NMR shieldings as well as the spin–spin coupling constants *J*_HH_ (Azzena et al., 2011; Carroccia et al., 2014; Zenzola et al., 2014; Degennaro et al., 2015a,b; Pisano et al., 2016)_._ All calculations were performed with the Gaussian 09 program at DFT level and the solvent effect was modeled using the self-consistent reaction field (SCRF) calculations within the SMD model (Marenich et al., 2009; Supplementary Material). The statistical parameters CMAE and R^2^ of δ_scaled_/δ_expt_ were determined to establish the consistency between the theoretical and experimental magnetic parameters of the two possible diastereoisomers, and the best fit in all cases was found for the diastereoisomer (*R, R, R*)-**6c** (see Supplementary Material). In Figure 2 the comparison between experimental and calculated ^1^H NMR spectra of azetidine **6c** is reported. A better match can be assessed between the real and calculated spectra of (*R,R,R*)-**6c** (Figures 2A,B).

**Scheme 5:**
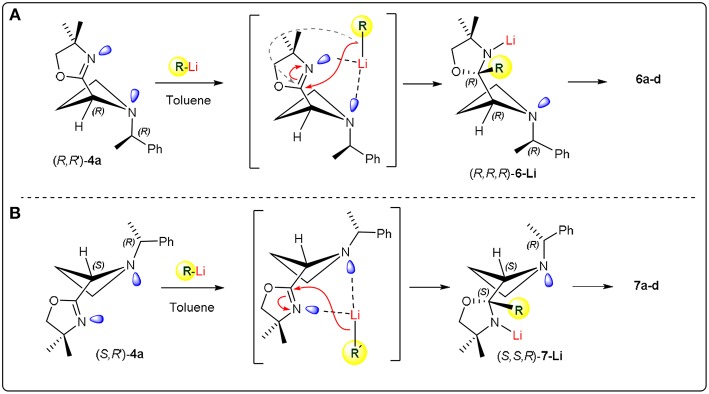
Proposed model.

Based on the results obtained by DFT calculations, we assumed that the configuration at the new created stereocentre of **6c** might be (*R*). With the aim to rationalize the stereochemical outcome of the nucleophilic addition of organolithiums to the C = N double bond of the oxazoline ring, we considered the stereodynamic model proposed for oxazolinylaziridines (Scheme 2). Assuming that oxazolinylazetidines could show dynamic phenomena associated with both nitrogen inversion and ring puckering (Parisi et al., 2016), the stereochemistry of substrates (*R, R*)**-4b** and (*R, S*)**-4b** was first assessed by NOESY experiments (see Supplementary Material). This stereochemical evaluation demonstrated a *trans* relationship between the nitrogen substituent and the oxazoline ring. Considering such stereochemical arrangement, the two lone pairs belonging to both azetidine and oxazoline nitrogens could likely be oriented in such a way to promote an easy formation of a complex with the organolithium reagent (Scheme 5). Under these stereochemical restrictions, it is reasonable to foresee the nucleophilic addition of the organolithium to the most accessible face of the planar C = N bond, resulting an (*R*) configuration at the new stereocenter starting from (*R, R*)**-4b**, and (*S*) configuration starting from (*R, S*)**-4b** (Scheme 5). On the basis of this model, the absolute configuration for compounds **6a-d** and **7a-b** have been supposed to be (*R,R,R*) and (*R,S,S*), respectively. Similar reasoning can be made for azetidine **4a** leading to adducts **5a-e** with (*R*,^*^R^*^) relative configuration. It is worth pointing out that, in striking contrast to what previously observed for aziridines, where a preferential α-lithiation took place under similar reaction conditions, with the nucleophilic addition occuring in 15–20% extent and low stereoselectivity, in the case of oxazolinylazetidines α-lithiation was never observed even at higher temperature. It is reasonable to assume that such peculiar stereoelectronic requirements realized with azetidines prevented the deprotonation event, leading to a stable complex prone to undergo exclusively nucleophilic addition to the C = N double bond.

### Synthesis of 2-acylazetidines

**Scheme 6:**
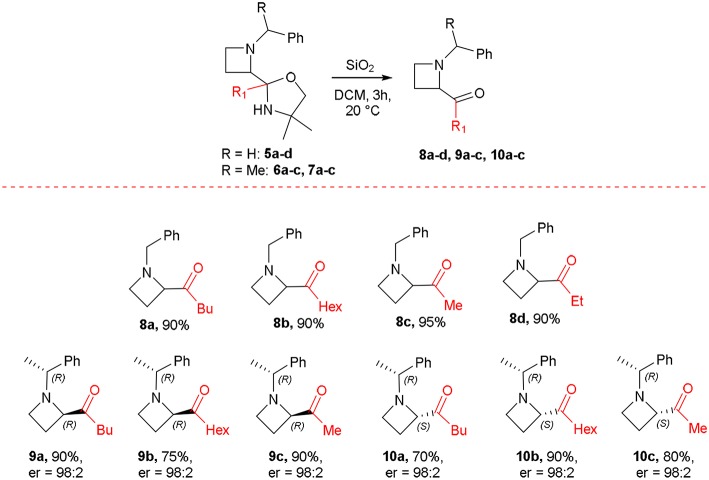
Synthesis of 2-acylazetidines.

All the products **5a-e** were isolated by flash chromatography, even though a partial and expected hydrolysis of the oxazolidine moiety in acidic media by silica gel occurred. This evidence prompted us to explore a mild and easy acidic hydrolysis by using silica gel in dichloromethane. Treatment of **5a-d** with SiO_2_ in dichloromethane (DCM) as solvent for 3 h afforded *N*-alkyl-2-acylazetidines **8a-d** in quantitative yields (Scheme 6). The oxazolidine **5e** resulted unreactive under these conditions, and all the attempts to hydrolyze the cyclic aminal failed. Similarly, the hydrolytic protocol was applied to compounds **6a-c** and **7b** giving good yields of 2-acylazetidines **9a-c** and **10b**, respectively, in enantiopure form as confirmed by HPLC analysis (see Supplementary Material). A partial epimerization for azetidine **9c** in acidic media by silica gel has been observed, leading to the formation of corresponding diastereoisomer **10c** (dr = 50/50). Moreover, complete hydrolysis of oxazolidines **7a** and **7c** taken place already during purification by chromatography on silica gel giving satisfactory yields of 2-acylazetidines **10a** and **10c**. The absolute stereochemistry of all optically active compounds was assigned according to data reported in the literature (Couty et al., 2011).

## Conclusions

We have demonstrated that in an apolar solvent, such as toluene, different organolithiums were capable to give an unexpected regio- and stereoselective addition at the C = N bond of the oxazoline group of *N*-alkyloxazolinylazetidines. Different 1,3-oxazolidinyl azetidines formed in high yield and resulted useful precursors of 2-acylazetidines by acidic hydrolysis. The expected deprotonation event in α position with respect to oxazoline ring did not take place in this conditions. With the aim to rationalize the mechanism and the stereochemical outcome of the addition reaction, a stereodynamic model has been proposed, taking into consideration complexation and dynamic phenomena associated with the azetidine's nitrogen inversion. The configuration assignment, performed on oxazolidinyl azetidines as intermediates by NMR and DFT calculations, resulted mandatory for the validation of proposed model. Even though the stereochemical information generated in the addition reaction is lost in the hydrolysis of oxazolidine ring, this work furnishes an outstanding example of reactivity controlled by dynamics of small nitrogenated heterocycles. Work is in progress to further explore the reactivity of 2-oxazolinylazetidines with organolithium in coordinating solvents.

## Materials and Methods

### General Information

Flash chromatography was performed using 70–230 mesh Al_2_O_3_ (either neutral or basic activity II-IV), with the indicated solvent system according to standard techniques. Analytical thin layer chromatography (TLC) was carried out on precoated 0.25 mm thick plates of Kieselgel 60 F254; visualization was accomplished by UV light (254 nm) or by spraying a solution of 5 % (w/v) ammonium molybdate and 0.2 % (w/v) cerium(III) sulfate in 100 ml 17.6 % (w/v) aq. sulphuric acid and heating to 200°C for some time until blue spots appear. Infrared spectra (v_max_, FT-IR) were recorded in reciprocal centimeters (cm^−1^). Nuclear magnetic resonance spectra were recorded on 300 or 500 MHz spectrometers. The frequency used to record the NMR spectra is given in each assignment and spectrum (^1^H NMR at 300 or 500 MHz; ^13^C NMR at 101 MHz or 176 MHz). Chemical shifts for ^1^H NMR spectra are recorded in parts per million with the residual protic solvent resonance as the internal standard (CDCl_3_: δ = 7.27 ppm). Data are reported as follows: chemical shift [multiplicity (s = singlet, d = doublet, t = triplet, q = quartet, p = pentet, m = multiplet and bs = broad singlet), coupling constant (in Hz), integration and assignment]. ^13^C NMR spectra were recorded with complete proton decoupling. Chemical shifts are reported in parts per million with the residual protic solvent resonance as the internal standard (CDCl_3_: δ = 77.0 ppm). Assignments of ^1^H and ^13^C spectra were based upon the analysis of δ and *J* values, as well as DEPT, COZY and HSQC experiments where appropriate. All reactions involving air sensitive reagents were performed under nitrogen in oven-dried glassware using syringe-septum cap technique. All organolithiums are commercially available by Sigma Aldrich and were titrated before use. All other chemicals were commercially available and used without further purification. Enantiomeric excess was assessed by HPLC (Chiralcel ODH, ADH). Diastereomeric ratio was assessed by GC-MS or ^1^H NMR analysis on the reaction crude.

## Synthesis of Substrates

### Methyl 1-benzyl-2-azetidinecarboxylate 2a

According to procedure reported in literature (Nocquet et al., 2012).

^**1**^**H NMR** (500 MHz, CDCl_3_) δ 7.45–7.14 (m, 5H, Ar-H overlapping CHCl_3_), 3.80 (d, *J* = 12.6 Hz, 1H, CH2Ph), 3.74 (t, *J* = 8.4 Hz, 1H, CHCO), 3.63 (s, 3H, CH_3_), 3.59 (d, *J* = 12.6 Hz, 1H, CH2Ph), 3.34–3.29 (m, 1H, NCH_2_), 2.94 (ddd, *J* = 9.2, 7.8, 6.9 Hz, 1H, NCH_2_), 2.41–2.32 (m, 1H, NCH_2_CH_2_), 2.21 (dtd, *J* = 10.5, 8.1, 2.4 Hz, 1H, NCH_2_CH_2_).

### Methyl 1-[(1*R*)-methyl]Benzylazetidine-(2*R*)-carboxylate (*R,R*)-2b

According to procedure reported in literature (Starmans et al., 1998).

^**1**^**H NMR** (300 MHz, CDCl_3_) δ 7.37–7.14 (m, 5H, Ar-H), 3.84–3.65 (m, 4H, CHCOOCH_3_), 3.45 (q, *J* = 6.6 Hz, 1H, CHCH_3_), 3.11 (ddd, *J* = 8.2, 7.7, 2.9 Hz, 1H, NCH_2_), 2.80 (td, *J* = 8.3, 7.1 Hz, 1H, NCH_2_), 2.36–2.10 (m, 2H, NCH_2_CH_2_), 1.22 (d, *J* = 6.6 Hz, 1H, CHCH_3_).

### Methyl 1-[(1*R*)-methyl]Benzylazetidine-(2*S*)-carboxylate (*R,S*)-2b

According to procedure reported in literature (Starmans et al., 1998).

^**1**^**H NMR** (500 MHz, CDCl_3_) δ 7.33–7.14 (m, 5H, Ar-H), 3.63–3.52 (m, 2H, CHCO and NCHH), 3.40–3.29 (m, 4H, CHPh and OCH_3_), 3.04–2.96 (m, 1H, NCHH), 2.36–2.24 (m, 1H NCH_2_CH_2_), 2.13 (dtd, *J* = 10.4, 8.0, 2.3 Hz, 1H, NCH_2_CH_2_), 1.28 (d, *J* = 6.5 Hz, 1H, CHCH_3_).

### 1-benzyl-*N*-(1-hydroxy-2-methylpropan-2-yl)azetidine-2-carboxamide 3a

General procedure A: To a perfectly dry flask charged with LaCl_3_ (587 mg, 2.40 mmol) was dried by heating under reduced pressure. Then, toluene dry (50 mL) and 2-amino-2-methylpropan-1-ol (4.11 g, 46.13 mmol) were added and after cooling to 0°C, *n*-hexyllithium (2.0 M in hexane, 23.0 mL, 46.13 mmol) was added dropwise and the reaction was heated to 100°C for 15 min. The solution was cooled to room temperature and methyl 1-benzylazetidine-2-carboxylate **2a** (3.78 g, 18.45 mmol) was added and the mixture was stirred over night. The reaction was quenched with water (1 mL) and filtered over celite. The organic phase was dried over sodium sulfate and the solvent was evaporated under reduced pressure to afford the product as a brown oil (4.50 g, yield 93%).

**FT-IR** (film) cm^−1^ 3321, 3054, 2984, 2932, 2856, 2305, 1649, 1532, 1454, 1422, 1265, 1071, 909, 738, and 705.

^**1**^**H NMR** (500 MHz, CDCl_3_) δ 7.35–7.27 (m, 3H, Ar-H), 7.25–7.20 (m, 2H, Ar-H), 7.04 (bs, 1H, NH), 5.08 (bs, 1H, OH), 3.66 (d, *J* = 12.3 Hz, 1H, CH_2_Ph), 3.62 (t, *J* = 8.6 Hz, 1H, CH), 3.51 (d, *J* = 12.3 Hz, 1H, CH_2_Ph), 3.45–3.38 (m, 2H, C*H*_2_OH and NCH_2_), 3.31 (d, *J* = 11.6 Hz, 1H, C*H*_2_OH), 3.08 (dd, *J* = 16.2, 8.7 Hz, 1H, NCH_2_), 2.47–2.38 (m, 1H, NCH_2_C*H*_2_), 2.08–1.95 (m, 1H, NCH_2_C*H*_2_), 1.15 (s, 1H, CH_3_), 0.96 (s, 1H, CH_3_).

^**13**^**C NMR** (126 MHz, CDCl_3_) δ 173.9 (C = O), 137.4 (Ar-C_q_), 129.1(2 x Ar-C), 128.9 (2 x Ar-C), 127.8 (Ar-C), 70.9 (CH_2_OH), 66.9 (CH), 62.7 (CH_2_Ph), 55.5 (C_q_), 51.0 (NCH_2_), 24.7 (CH_3_), 24.4 (CH_3_), 23.2 (NCH_2_*C*H_2_).

**HRMS** (ESI-TOF) [M+Na]^+^ calculated for C_15_H_22_N_2_NaO_2_: 285.1579, found 285.1558.

### (*R*)-*N*-(1-hydroxy-2-methylpropan-2-yl)-1-[(*R*)-1-phenylethyl]azetidine-2-carboxamide (*R,R*)-3b

According to the general procedure A, starting from (*R,R*)-**2b** (800 mg, 3.65 mmol) amide (*R,R*)-**3b** was isolated as white solid, 907 mg, yield 90%, mp 107–109°C, ${\text{[α]}}_{\text{D}}^{20}$ = + 69.49° (*c* = 1, CHCl_3_).

**FT-IR** (KBr, cm^−1^) ν 3334, 3234, 2969, 2852, 1645, 1538, 1451, 1377, 1282, 1072, 767, 702, and 565.

^**1**^**H NMR** (500 MHz, CDCl_3_): δ 7.70 (s, 1H, NH-OH), 7.34–7.21 (m, 5H, Ar-H), 5.24 (s, 1H, NH - OH), 3.75–3.64 (m, 3H, C*H*_2_OH and NC*H*), 3.45 (q, *J* = 6.4 Hz, 1H, C*H*CH_3_), 3.08 (t, *J* = 8.0 Hz, 1H, CH_2_), 2.78 (q, *J* = 8.0 Hz, 1H, CH_2_), 2.38–2.29 (m, 1H, CH_2_), 2.00–1.91 (m, 1H, CH_2_), 1.36 (s, 3H, CH_3_), 1.33 (s, 3H, CH_3_), 1.14 (d, *J* = 6.6 Hz, 3H, CHC*H*_3_).

^**13**^**C NMR** (125 MHz, CDCl_3_): δ 174.7 (C = O), 142.61 (Ar-C_q_), 128.6 (2x Ar-C), 127.4 (Ar-C), 126.9 (2x Ar-C), 70.8 (CH_2_OH), 67.3 (*C*H-Ph), 65.3 (NCH), 55.6 (C_q_), 49.6 (CH_2_N), 24.8 (CH_3_), 24.7 (CH_3_), 22.1 (CH_2_*C*H_2_CH), 21.2 (*C*H_3_CH).

**HRMS** (ESI-TOF) [M+H]^+^ calculated for C_16_H_25_N_2_O_2_: 277.1911, found 277.1917.

### (*S*)-*N*-(1-hydroxy-2-methylpropan-2-yl)-1-[(*R*)-1-phenylethyl]azetidine-2-carboxamide (*R,S*)-3b

According to the general procedure A, starting from (*S,R*)-**2b** (860 mg, 3.94 mmol) amide (*R,S*)-**3b** was isolated as colorless oil, 979 mg, yield 90%, ${\text{[α]}}_{\text{D}}^{20}$ = −118.15° (*c* = 0.4, CHCl_3_).

**FT-IR** (film, cm^−1^) ν 3323, 2969, 2930, 2850, 1651, 1532, 1456, 1380, 1278, 1168, 1071, 709, and 656.

^**1**^**H NMR** (500 MHz, CDCl_3_) δ 7.29 (m, 5H, Ar-H), 6.71 (bs, 1H, NH), 4.99 (t, *J* = 6.1 Hz, 1H, OH), 3.57 (t, *J* = 8.6 Hz, 1H, CH-CO), 3.46 (t, *J* = 7.6 Hz, 1H, CH_2_N), 3.40–3.28 (m, 2H, CHPh and C*H*HN), 3.15–3.05 (m, 2H, C*H*HN and C*H*HOH), 2.44–2.27 (m, 1H, CH_2_), 1.99–1.86 (m, 1H, CH_2_), 1.32 (d, *J* = 6.6 Hz, 3H, CHC*H*_3_), 1.11 (s, 3H, CH_3_), 0.88 (s, 3H, CH_3_).

^**13**^**C NMR** (126 MHz, CDCl_3_): δ 173.9 (C = O), 141.5 (Ar-C_q_), 128.9 (2 x Ar-C), 128.2 (Ar-C), 128.0 (2 x Ar-C), 71.0 (CH_2_OH), 67.0 (CH-Ph), 66.0 (*C*HCH_2_), 55.3 (C_q_), 49.8 (CH_2_N), 24.4 (CH_3_), 24.1 (CH_3_), 22.47 (CH_2_*C*H_2_CH), 18.3 (*C*H_3_CH).

**HRMS** (ESI-TOF) [M+H]^+^ calculated for C_16_H_25_N_2_O_2_: 277.1911, found 277.1933.

General procedure B (cyclization to 4,5-dihydrooxazole): To a solution of hydroxamide (943 mg, 3.42 mmol) in dry dichloromethane (23 mL) at −78°C, diethylaminosulfur trifluoride (497 μL, 3.76 mmol) was added dropwise and the reaction was stirred for 1 h. Then the reaction was stirred at room temperature overnight. The solution was washed with NaHCO_3_ 0.1 M (3 × 7 mL), the organic phase was dried over Na_2_SO_4_ and the solvent was evaporated under reduced pressure.

### 2-(1-benzylazetidin-2-yl)-4,4-dimethyl-4,5-dihydrooxazole 4a

According to the general procedure B, dihydrooxazole **4a** was isolated as yellow oil by chromatography on alumina (20% AcOEt/hexane, Rf 0.4) (1.18 g, yield 88%).

**FT-IR** (film, cm^−1^) 3062, 3027, 2966, 2928, 2865, 1666, 1454, 1363, 1297, 1174, 1004, 935, 735, and 701.

^**1**^**H NMR** (700 MHz, CDCl_3_) δ 7.31–7.27 (m, 4H, Ar-H), 7.22 (t, *J* = 7.1 Hz, 1H, Ar-H), 3.86 (d, *J* = 8.0 Hz, 1H, OCH_2_), 3.82 (t, *J* = 8.2 Hz, 1H, azetidine-CH), 3.77 (d, *J* = 8.0 Hz, 1H, OCH_2_), 3.67 (AB system, d, *J* = 12.6 Hz, 2H, CH_2_Ph), 3.41–3.37 (m, 1H, azetidine-CH_2_), 3.00 (dt, *J* = 9.4, 7.4 Hz, 1H, azetidine-CH_2_), 2.45–2.37 (m, 1H, azetidine-CH_2_), 2.16 (m, 1H, azetidine-CH_2_), 1.18 (s, 3H, CH_3_), 1.07 (s, 3H, CH_3_).

^**13**^**C NMR** (126 MHz, CDCl_3_): δ 165.2 (C = N), 137.4 (Ar-C_q_), 129.4 (2 × Ar-C), 128.3 (2 × Ar-C), 127.2 (Ar-C), 79.23 (oxazoline-CH_2_), 66.9 (oxazoline-C_q_), 62.8 (CH_2_Ph), 61.5 (azetidine-CH), 51.8 (azetidine-CH_2_), 28.3 (CH_3_), 28.3 (CH_3_) 22.0 (azetidine-CH_2_).

**HRMS** (ESI-TOF) [M+H]^+^ calculated for C_15_H_21_N_2_O: 245.1654, found 245.1649.

### 4,4-dimethyl-2-{(*R*)-1-[(*R*)-1-phenylethyl]azetidin-2-yl}-4,5-dihydrooxazole (*R,R*)-4b

According to the general procedure B, dihydrooxazole **(*R,R*)-4b** was isolated as light brown oil by chromatography on silica (40% AcOEt/hexane, Rf 0.5) (536 mg, yield 92%), ${\text{[α]}}_{\text{D}}^{20}$ = + 81.20° (*c* = 1, CHCl_3_). (ADH, 99:1 Hex:iPrOH, 1 mL/min).

**FT-IR** (film, cm^−1^) ν 2967, 2929, 2869, 1737, 1660, 1493, 1453, 1364, 1175, 1072, 1028, 1004, 975, 764, and 701.

^**1**^**H NMR** (500 MHz, CDCl_3_): δ 7.35–7.26 (m, 4H, Ar-H), 7.24–7.20 (m, 1H, Ar-H), 4.04–3.98 (m, 2H, OCH_2_), 3.87 (t, *J* = 8.1 Hz, 1H, NCH), 3.49–3.40 (m, 1H, CH-Ph), 3.12–3.04 (m, 1H, CH_2_), 2.82–2.73 (m, 1H, CH_2_), 2.38–2.29 (m, 1H, CH_2_), 2.14–2.06 (m, 1H, CH_2_), 1.29 (s, 3H, CH_3_), 1.28 (s, 3H, CH_3_), 1.23 (d, *J* = 6.4 Hz, 3H, CHC*H*_3_).

^**13**^**C NMR** (126 MHz, CDCl_3_): δ 166.3 (C = N), 142.9 (Ar-C_q_), 128.4 (2 × Ar-C), 127.6 (2 × Ar-C), 127.2 (Ar-C), 79.4 (OCH_2_), 68.1 (CH-Ph), 67.0 (C_q_), 61.2 (NCH), 50.2 (CH_2_), 28.2 (CH_3_), 28.2 (CH_3_), 21.1 (CH*C*H_3_), 21.07 (CH_2_).

**HRMS** (ESI-TOF) [M+H]^+^ calculated for C_16_H_23_N_2_O: 259.1805, found 259.1805.

### 4,4-dimethyl-2-{(*S*)-1-[(*R*)-1-phenylethyl]azetidin-2-yl}-4,5-dihydrooxazole (*R,S*)-4b

According to the general procedure B, dihydrooxazole **(*R,S*)-4b** was isolated as light yellow oil by chromatography on silica (40% AcOEt/hexane, Rf 0.4) (747 mg, yield 94%), ${\text{[α]}}_{\text{D}}^{20}$ = −56.30° (*c* = 1, CHCl_3_). (ADH, 99:1 Hex:iPrOH, 0.5 mL/min).

**FT-IR** (film, cm^−1^) ν 2960, 2932, 2874, 1735, 1658, 1497, 1444, 1371, 1168, 1072, 1022, 1010, 985, 760, and 704.

^**1**^**H NMR** (500 MHz, CDCl_3_) δ 7.34–7.16 (m, 5H overlapping CHCl_3_, Ar-H), 3.76–3.68 (m, 2H, OC*H*H and C*H*CH_2_), 3.58–3.49 (m, 2H, OC*H*H and NC*H*H), 3.34 (q, *J* = 6.5 Hz, 1H, C*H*CH_3_), 2.99 (q, *J* = 7.7 Hz, 1H, OC*H*H), 2.38–2.27 (m, 1H, C*H*_2_CH), 2.13–2.04 (m, 1H, C*H*_2_CH), 1.26 (d, *J* = 6.6 Hz, 3H, C*H*_3_CH), 1.03 (s, 1H, CH_3_), 0.81 (s, 1H, CH_3_).

^**13**^**C NMR** (126 MHz, CDCl_3_): δ 164.8 (C = N), 142.6 (Ar-C_q_), 128.3 (2 × Ar-C), 128.1 (2 × Ar-C), 127.4 (Ar-C), 79.0 (0CH_2_), 68.5 (CHPh), 66.5 (C_q_), 61.4 (NCH), 50.9 (NCH_2_), 28.2 (CH_3_), 28.0 (CH_3_), 21.3 (*C*H_2_CH), 20.3 (*C*H_3_CH).

**HRMS** (ESI-TOF) [M+H]^+^ calculated for C_16_H_23_N_2_O: 259.1805, found 259.1805.

## Addition TO 4,5-dihydrooxazoles

General procedure: To a solution of 2-(1-benzylazetidin-2-yl)-4,4-dimethyl-4,5-dihydrooxazole **4a** (1 eq) in dry toluene cooled at −78°C, organolithium (R-Li, 1.1-1.5 eq) was added dropwise. The reaction was stirred for 20 min and quenched with water (1 mL). The crude was extracted with water/ethyl acetate and the collected organic phases were dried over sodium sulfate. The solvent was evaporated under reduced pressure and the alumina chromatography afforded the desire product.

### (R^*^, R^*^)-2-(1-benzylazetidin-2-yl)-2-butyl-4,4-dimethyl-1,3-oxazolidine 5a

According to the General Procedure, the reaction was carried out using 2-(1-benzylazetidin-2-yl)-4,4-dimethyl-4,5-dihydrooxazole **4a** (60 mg, 0.25 mmol) in dry toluene (5 mL) and butyllithium (1.15 M in hexane, 235 μL, 0.27 mmol) affording **5a** as yellow oil (66 mg, yield 87%, dr = 98:2). R_f_ 0.9 (20% AcOEt/hexane).

**FT-IR** (film, cm^−1^) 3265, 2958, 2928, 2857, 1599, 1463, 1364, 1268, 1142, 1045, 935, 799, and 732.

^**1**^**H NMR** (500 MHz, CDCl_3_) δ 7.31–7.21 (m, 5H, Ar-H), 4.03 (d, *J* = 13.6 Hz, 1H, CH_2_Ph), 3.63 (d, *J* = 7.6 Hz, 1H, oxazolidine-CH_2_), 3.54 (t, *J* = 8.4 Hz, 1H, CH), 3.41 (d, *J* = 7.6 Hz, 1H, oxazolidine-CH_2_), 3.39 (d, *J* = 13.6 Hz, 1H, CH_2_Ph), 3.15 (m, 1H, azetidine-CH_2_), 2.71 (dd, *J* = 15.6, 8.7 Hz, 1H, azetidine-CH_2_), 1.99–1.91 (m, 2H, azetidine-CH_2_), 1.80–1.68 (m, 1H, butyl-H), 1.55–1.46 (m, 1H, butyl-H), 1.34 (s, 3H, oxazoline-CH_3_), 1.33 (s, 3H, oxazolidine-CH_3_), 1.31–1.23 (m, 5H, butyl-H), 0.90 (t, *J* = 7.0 Hz, 1H, CH_2_-C*H*_3_).

^**13**^**C NMR** (176 MHz, CDCl_3_) δ 138.8 (Ar-C_q_), 128.4 (2 × Ar-C), 128.3 (2 × Ar-C), 126.9 (Ar-C), 100.6 (OC_q_NH), 77.4 (OCH_2_), 68.7 (CH), 62.9 (CH_2_Ph) 62.8 (butyl-CH_2_), 59.6 (butyl-CH_2_), 50.52 (NCH_2_), 36.9 (butyl-CH_2_), 29.9 (oxazolidine-CH_3_), 29.1 (oxazolidine-CH_3_), 28.0 (butyl-CH_2_), 26.51 (butyl-CH_2_), 23.55 (butyl-CH_2_), 20.9 (NCH_2_CH_2_), 14.17 (butyl-CH_3_).

**HRMS** (ESI-TOF) [M+H]^+^ calculated for C_19_H_31_N_2_O: 303.2436, found 303.2428.

### (R^*^, R^*^)-2-(1-benzylazetidin-2-yl)-2-hexyl-4,4-dimethyl-1,3-oxazolidine 5b

According to the General Procedure, the reaction was carried out using 2-(1-benzylazetidin-2-yl)-4,4-dimethyl-4,5-dihydrooxazole **4a** (70 mg, 0.29 mmol) in dry toluene (6 mL) and hexyllithium (1.25 M in hexane, 255 μL, 0.32 mmol) affording **5b** as yellow oil (58 mg, yield 60%, dr = 98:2), R_f_ 0.9 (20% AcOEt/hexane).

^**1**^**H NMR** (500 MHz, CDCl_3_) δ 7.41–7.10 (m, 5H, Ar-H overlapping CHCl_3_ signal), 4.03 (d, *J* = 13.5 Hz, 1H, CH_2_Ph), 3.63 (d, *J* = 7.5 Hz, 1H, oxazolidine-H), 3.54 (t, *J* = 8.5 Hz, 1H, CH), 3.44 – 3.33 (m, 2H, CH_2_Ph and oxazolidine-H), 3.17–3.12 (m, 1H, azetidine-CH_2_), 2.70 (q, *J* = 8.0 Hz, 1H, azetdine-CH_2_), 1.94 (m, 2H, azetdine-CH_2_), 1.77–1.68 (m, 1H, hexyl-CH_2_), 1.49 (t, *J* = 10.3 Hz, 1H, hexyl-CH_2_), 1.34 (s, 3H, oxazoline- CH_3_), 1.32 (s, 3H, oxazoline-CH_3_), 1.31–1.23 (m, 9H, hexyl-CH_2_), 0.88 (t, *J* = 6.2 Hz, 3H, CH_2_-CH_3_).

^**13**^**C NMR** (126 MHz, CDCl_3_) δ 138.7 (Ar-C_q_), 128.4 (2 × Ar-C), 128.3 (2 × Ar-C), 126.9 (Ar-C) 100.6 (OC_q_N), 77.4 (oxazolidine-CH_2_), 68.6 (azetidine-CH), 62.8 (CH_2_Ph), 59.6 (oxazolidine-C_q_), 50.5 (azetidine-CH_2_), 37.2 (hexyl-CH_2_), 31.9 (hexyl-CH_2_), 30.15 (hexyl-CH_2_), 29.11 (oxazolidine-CH_3_), 28.05 (oxazolidine-CH_3_), 24.3 (hexyl-CH_2_), 22.7 (hexyl-CH_2_), 20.9 (azetidine-CH_2_), 14.25 (hexyl-CH_3_).

**HRMS** (ESI-TOF) [M+H]^+^ calculated for C_21_H_35_N_2_O: 331.2749, found 331.2743.

### (R^*^, R^*^)-2-(1-benzylazetidin-2-yl)-2,4,4-trimethyl-1,3-oxazolidine 5c

According to the General Procedure, the reaction was carried out using 2-(1-benzylazetidin-2-yl)-4,4-dimethyl-4,5-dihydrooxazole **4a** (50 mg, 0.21 mmol) in dry toluene (5 mL) and methyllithium (1.1 M in diethoxy methane, 210 μL, 0.23 mmol) affording 2-(1-benzylazetidin-2-yl)-2,4,4-trimethyloxazolidine **5c** as yellow oil (37 mg, yield 70%, dr = 98:2). R_f_ 0.9 (5% AcOEt/hexane).

**FT-IR** (film, cm^−1^) 3029, 2963, 2928, 2853, 1637, 1454, 1371, 1261, 1044, 797, and 748.

^**1**^**H NMR** (300 MHz, CDCl_3_): δ 7.39–7.11 (m, 5H, Ar-H overlapping CHCl_3_ signal), 4.01 (d, *J* = 13.6 Hz, 1H, CH_2_Ph), 3.68 (d, *J* = 8.2 Hz, 1H, OCH_2_), 3.43–3.33 (m, 3H, CH_2_Ph, OCH_2_ and azetidine-CH), 3.15 (t, *J* = 6.5 Hz, 1H, azetidine-CH_2_), 2.73 (q, *J* = 7.9 Hz, 1H, azetidine-CH_2_), 2.02–1.86 (m, 2H, azetidine-CH_2_), 1.34 (s, 6H, oxazolidine-CH_3_), 1.25 (s, 1H, CH_3_).

^**13**^**C NMR** (126 MHz, CDCl_3_) δ 138.7 (Ar-C_q_), 128.4 (2 × Ar-C), 128.4 (2 × Ar-C), 127.0 (Ar-C), 98.8 (OC_q_N), 77.7 (OCH_2_), 70.8 (azetidine-CH), 62.6 (CH_2_Ph), 59.9 (oxazoline-C_q_), 50.2 (azetidine-CH_2_), 28.8 (oxazolidine-CH_3_), 27.5 (oxazolidine-CH_3_), 23.5 (CH_3_), 20.8 (azetidine-CH_2_).

**HRMS** (ESI-TOF) [M+H]^+^ calculated for C_16_H_25_N_2_O: 261.1967, found 261.1957.

### (R^*^, R^*^)-2-(1-benzylazetidin-2-yl)-2-ethyl-4,4-dimethyl-1,3-oxazolidine 5d

According to the General Procedure, the reaction was carried out using 2-(1-benzylazetidin-2-yl)-4,4-dimethyl-4,5-dihydrooxazole **4a** (120 mg, 0.49 mmol) in dry toluene (10.5 mL) and ethyllithium (0.1 M in benzene/cyclohexane, 5.4 mL, 0.54 mmol) affording 2-(1-benzylazetidin-2-yl)-2-ethyl-4,4-dimethyloxazolidine **5d** as colorless oil (78 mg, yield 58%, dr = 98:2). R_f_ 0.9 (10% AcOEt/hexane).

**FT-IR** (film, cm^−1^) 3267, 2963, 2927, 2855, 1455, 1366, 1260, 1208, 1045, 916, 798, 734, and 697.

^**1**^**H NMR** (500 MHz, CDCl_3_) δ 7.34–7.27 (m, 4H, Ar-H), 7.25–7.20 (m, 1H, Ar-H), 4.03 (d, *J* = 13.6 Hz, 1H, oxazolidine-CH_2_), 3.62 (d, *J* = 7.7 Hz, 1H, CH_2_Ph), 3.56 (t, *J* = 8.5 Hz, 1H, azetidine-CH), 3.42 (d, *J* = 7.7 Hz, 1H, CH_2_Ph), 3.39 (d, *J* = 13.6 Hz, 1H, oxazolidine-CH_2_), 3.15 (td, *J* = 6.8, 3.8 Hz, 1H, azetidine-CH_2_), 2.76–2.68 (m, 1H, azetidine-CH_2_), 1.98–1.91 (m, 2H, azetidine-CH_2_), 1.84–1.74 (m, 1H, C*H*_2_CH_3_), 1.52 (tt, *J* = 14.4, 7.2 Hz, 1H, C*H*_2_CH_3_), 1.34 (s, 3H, oxazolidine-CH_3_), 1.32 (s, 3H, oxazolidine-CH_3_), 0.91 (t, *J* = 7.6 Hz, 3H, CH_2_C*H*_3_).

^**13**^**C NMR** (126 MHz, CDCl_3_) δ 138.7 (Ar-C_q_), 128.4 (2 × Ar-C), 128.3 (2 × Ar-C), 126.9 (Ar-C), 100.7 (OC_q_N), 77.3 (OCH_2_), 68.1 (azetidine-CH), 62.8 (CH_2_Ph), 59.6 (C_q_), 50.5 (azetidine-CH_2_), 29.5 (*C*H_2_CH_3_), 29.0 (oxazolidine-CH_3_), 28.1 (oxazoline-CH_3_), 20.8 (azetidine-CH_2_), 8.68 (CH_2_*C*H_3_).

**HRMS** (ESI-TOF) [M+H]^+^ calculated for C_17_H_27_N_2_O: 275.2123, found 275.2113.

### (R^*^, R^*^)-2-(1-benzylazetidin-2-yl)-4,4-dimethyl-2-phenyl-1,3-oxazolidine 5e

According to the General Procedure, the reaction was carried out using 2-(1-benzylazetidin-2-yl)-4,4-dimethyl-4,5-dihydrooxazole **4a** (73 mg, 0.30 mmol) in dry toluene (7 mL) and phenyllithium (1.0 M in dibutyl ether, 330 μL, 0.33 mmol) affording 2-(1-benzylazetidin-2-yl)-4,4-dimethyl-2-phenyloxazolidine **5e** as colorless oil (79 mg, yield 82%, dr = 98:2). R_f_ 0.9 (5% AcOEt/hexane).

**FT-IR** (film, cm^−1^) 3262, 3084, 3061, 3028, 3001, 2963, 2928, 2857, 1953, 1887, 1812, 1652, 1494, 1453, 1366, 1235, 1038, 941, 738, and 700.

^**1**^**H NMR** (300 MHz, CDCl_3_) δ 7.62 (d, *J* = 8.1, 2H, Ar-H), 7.35–7.21 (m, 8H, Ar-H), 4.17 (d, *J* = 13.4 Hz, 1H, CH_2_Ph), 3.51–3.41 (m, 4H, *H*CHPh, oxazolidine-CH_2_ overlapping azetidine-CH), 3.18–3.09 (m, 1H, azetidine-CH_2_), 2.63 (dd, *J* = 16.2, 7.7 Hz, 1H, azetidine-CH_2_), 1.96 (dt, *J* = 17.9, 9.0 Hz, 1H, azetidine-CH_2_), 1.52 (dtd, *J* = 10.2, 7.9, 2.1 Hz, 1H, azetidine-CH_2_), 1.38 (s, 3H, CH_3_), 0.93 (s, 3H, CH_3_).

^**13**^**C NMR** (176 MHz, CDCl_3_) δ 143.1 (Ar-C_q_), 138.9 (Ar-C_q_), 128.5 (2 × Ar-C), 128.4 (2 × Ar-C), 127.8 (2 x Ar-C), 127.4 (Ar-C), 126.9 (Ar-C), 126.9 (2 × Ar-C), 101.2 (OC_q_N), 77.3 (OCH_2_), 70.8 (CH), 62.6 (CH_2_Ph), 60.1 (C_q_), 49.7 (azetidine-CH_2_), 28.00 (CH_3_), 27.9 (CH_3_), 20.6 (azetidine-CH_2_).

**HRMS** (ESI-TOF) [M+H]^+^ calculated for C_21_H_27_N_2_O: 323.2123, found 323.2109.

### (*R*)-2-butyl-4,4-dimethyl-2-{(*R*)-1-[(*R*)-1-phenylethyl]azetidin-2-yl}-1,3-oxazolidine 6a

According to the General Procedure, the reaction was carried out using dihydrooxazole (*R,R*)-**4b** (50 mg, 0.19 mmol) in dry toluene (3 mL) and butyllithium (1.15 M in hexane, 235 μL, 0.28 mmol) affording **6a** as a pale yellow oil, yield 86%, 53 mg, dr = 95:5, ${\text{[α]}}_{\text{D}}^{20}$ = + 59.15° (*c* = 1, CHCl_3_).

**FT-IR** (film, cm^−1^) ν 3412, 3028, 2960, 2930, 2860, 1492, 1453, 1380, 1260, 1227, 1164, 1047, 968, 859, 761, and 701.

^**1**^**H NMR** (500 MHz, CDCl_3_) δ 7.35–7.30 (m, 2H, Ar-H), 7.29–7.22 (m, 3H, Ar-H), 3.65 (m, 2H, CHPh and OCH_2_), 3.58 (d, *J* = 7.8 Hz, 1H, OCH_2_), 3.49 (t, *J* = 8.5 Hz, 1H, NCH), 3.00–2.95 (m, 1H, NCH_2_), 2.67 (q, *J* = 8.8 Hz, 1H, NCH_2_), 1.92–1.70 (m, 3H, 2 x NCH_2_C*H*_2_ and butyl-H), 1.49–1.35 (m, 8H, 2 × butyl-H and 2 x oxazolidin-CH_3_), 1.30 (d, *J* = 6.9 Hz, 3H, CHC*H*_3_), 1.29–1.24 (m, 2H, butyl-H), 1.23–1.16 (m, 1H, butyl-H), 0.87 (t, *J* = 7.2 Hz, 3H, butyl-CH_3_).

^**13**^**C NMR** (126 MHz, CDCl_3_): δ 142.2 (Ar-C) 128.2 (2 × Ar-C), 128.2 (2 × Ar-C), 127.0 (Ar-C), 100.7 (NHC_q_O), 77.2 (OCH_2_), 65.4 (NCH), 64.2 (*C*HCH_3_), 59.3 (oxazolidine-C_q_), 46.2 (NCH_2_), 35.9 (butyl-CH_2_), 30.0 (oxazolidine-CH_3_), 28.9 (oxazolidine-CH_3_), 26.3 (butyl-CH_2_), 23.5 (butyl-CH_2_), 20.9 (CH*C*H_3_), 20.1 (NCH_2_CH_2_), 14.2 (butyl-CH_3_).

**HRMS** (ESI-TOF) [M+H]^+^ calculated for C_20_H_33_N_2_O: 317.2587, found 317.2587.

### (*R*)-2-hexyl-4,4-dimethyl-2-{(*R*)-1-[(*R*)-1-phenylethyl]azetidin-2-yl}-1,3-oxazolidine 6b

According to the General Procedure, the reaction was carried out using dihydrooxazole (*R,R*)-**4b** (60 mg, 0.23 mmol) in dry toluene (4 mL) and hexyllithium (2.0 M in hexane, 170 μL, 0.35 mmol) affording **6b** as yellow oil, 66 mg, yield 83%, dr = 95:5, R_f_ 0.9 (20% AcOEt/hexane), ${\text{[α]}}_{\text{D}}^{20}$ = + 69.49° (*c* = 1, CHCl_3_).

**FT-IR** (film, cm^−1^) ν 3261, 2959, 2927, 2856, 1492, 1454, 1380, 1265, 1210, 1164, 1047, 928, 851, 761, 701, and 665.

^**1**^**H NMR** (500 MHz, CDCl_3_) δ 7.35–7.30 (m, 2H, Ar-H), 7.28–7.22 (m, 3H, Ar-H), 3.67–3.61 (m, 2H, CHPh and OCH_2_), 3.58 (d, *J* = 7.8 Hz, 1H, OCH_2_), 3.49 (t, *J* = 8.5 Hz, 1H, NCH), 3.00–2.95 (m, 1H, NCH_2_), 2.67 (q, *J* = 8.8 Hz, 1H, NCH_2_), 1.93–1.70 (m, 3H, NCH_2_C*H*_2_ and hexyl-H), 1.49–1.40 (m, 1H, hexyl-H), 1.39 (s, 3H, oxazolidine-CH_3_), 1.36 (s, 3H, oxazolidine-CH_3_), 1.32–1.21 (m, 9H, CHC*H*_3_ and 6 × hexyl-H), 0.87 (t, *J* = 7.0 Hz, 3H, hexyl-CH_3_).

^**13**^**C NMR** (126 MHz, CDCl_3_) δ 142.2 (Ar-C_q_), 128.2 (2 × Ar-C), 128.2 (2 × Ar-C), 127.0 (Ar-C), 100.7 (NHC_q_O), 77.2 (OCH_2_), 65.4 (NCH), 64.2 (CHCH_3_), 59.3 (oxazolidine-C_q_), 46.2 (NCH_2_), 36.2 (hexyl-CH_2_), 31.9 (hexyl-CH_2_), 30.1 (oxazolidine-CH_3_), 30.0 (hexyl-CH_2_), 28.9 (oxazolidine-CH_3_), 24.1 (hexyl-CH_2_), 22.7 (hexyl-CH_2_), 20.9 (CHCH_3_), 20.1 (NCH_2_CH_2_), 14.2 (hexyl-CH_3_).

**HRMS** (ESI-TOF) [M+H]^+^ calculated for C_22_H_37_N_2_O: 345.2900, found 345.2894.

### (*R*)-2,4,4-trimethyl-2-{(*R*)-1-[(*R*)-1-phenylethyl]azetidin-2-yl}-1,3-oxazolidine 6c

According to the General Procedure, the reaction was carried out using dihydrooxazole (*R,R*)-**4b** (50 mg, 0.19 mmol) in dry toluene (3 mL) and methyllithium (1.0 M in diethoxymethane, 290 μL, 0.29 mmol) affording oxazolidine **6c** as yellow oil, 36 mg, yield 70%, dr = 95:5). R_f_ 0.9 (5% AcOEt/hexane). ${\text{[α]}}_{\text{D}}^{20}$ = + 69.10° (*c* = 1, CHCl_3_).

**FT-IR** (film, cm^−1^) ν 3258, 2965, 2928, 2861, 1707, 1452, 1371, 1262, 1230, 1166, 1046, 846, and 701.

^**1**^**H NMR** (300 MHz, CDCl_3_) δ 7.37–7.20 (m, 5H, Ar-H overlapping CHCl_3_), 3.72 (d, *J* = 7.7 Hz, 1H, OCH_2_), 3.64 (q, *J* = 6.8 Hz, 1H, CHPh), 3.53 (d, *J* = 7.7 Hz, 1H, OCH_2_), 3.34 (t, *J* = 8.5 Hz, 1H, NCH), 2.99 (dd, *J* = 12.4, 6.1 Hz, 1H, NCH_2_), 2.69 (td, *J* = 8.9, 7.1 Hz, 1H, NCH_2_), 1.85 (td, *J* = 8.8, 6.0 Hz, 2H, NCH_2_C*H*_2_), 1.39 (s, 1H, oxazolidine-CH_3_), 1.38 (s, 1H, oxazolidine-CH_3_), 1.31 (d, *J* = 6.9 Hz, 1H, CHCH_3_), 1.21 (s, 1H, oxazolidine-CH_3_).

^**13**^**C NMR** (126 MHz, CDCl_3_) δ 142.1 (Ar-C_q_), 128.3 (2 × Ar-C), 128.2 (2 × Ar-C), 127.1 (Ar-C), 99.0 (OC_q_NH), 77.5 (OCH_2_), 67.6 (NCH), 64.1 (CHPh), 59.6 (oxazolidine-C_q_), 46.1 (NCH_2_), 29.7 (oxazolidine-CH_3_), 28.4 (oxazolidine-CH_3_), 23.0 (oxazolidine-CH_3_), 20.8 (CH*C*H_3_), 20.2 (NCH_2_).

**HRMS** (ESI-TOF) [M+Na]^+^ calculated for C_17_H_26_N_2_NaO: 297.1943, found 297.1946.

### (*R*)-4,4-dimethyl-2-phenyl-2-{(*R*)-1-[(*R*)-1-phenylethyl]azetidin-2-yl}-1,3-oxazolidine 6d

According to the General Procedure, the reaction was carried out using dihydrooxazole (*R,R*)-**4b** (50 mg, 0.19 mmol) in dry toluene (3 mL) and phenyllithium (1.0 M in dibutyl ether, 290 μL, 0.29 mmol) affording oxazolidine **6d** as colorless oil, 62 mg, yield 95%, dr = 95:5). R_f_ 0.8 (10% AcOEt/hexane). ${\text{[α]}}_{\text{D}}^{20}$ = + 40.10° (*c* = 1, CHCl_3_). (ADH, 99.5:0.5 Hex:iPrOH + 0.2% DEA, 0.3 mL/min).

**FT-IR** (film, cm^−1^) ν 3257, 3026, 2927, 2865, 1450, 1382, 1268, 1200, 1164, 1039, 961, 851, 752, 720, 701, and 647.

^**1**^**H NMR** (500 MHz, CDCl_3_) δ 7.62–7.58 (m, 2H, Ar-H), 7.37–7.20 (m, 8H, Ar-H), 3.75–3.68 (m, 1H, CHPh), 3.55 (d, *J* = 7.5 Hz, 1H, OCH_2_), 3.47–3.38 (m, 2H, NCH and OCH_2_), 3.01–2.90 (m, 1H, NCH_2_), 2.60–2.52 (m, 1H, NCH_2_), 1.87–1.77 (m, 1H, NCH_2_C*H*_2_), 1.44 (s, 3H, oxazolidine-CH_3_), 1.41 (d, *J* = 6.7 Hz, 1H, CHC*H*_3_), 1.33–1.23 (m, 1H, NCH_2_C*H*_2_), 0.99 (s, 1H, oxazolidine-CH_3_).

^**13**^**C NMR** (126 MHz, CDCl_3_) δ 142.9 (Ar-C_q_), 142.5 (Ar-C_q_), 128.2 (2 × Ar-C), 128.2 (2 × Ar-C), 127.6 (2 × Ar-C), 127.4 (Ar-C), 127.3 (2 × Ar-C), 125.8 (Ar-C), 101.2 (NHC_q_O), 77.1 (OCH), 68.0 (NCH), 64.4 (CHPh), 59.9 (oxazolidine-C_q_), 45.9 (NCH_2_), 28.6 (2 × oxazolidine-CH_3_), 21.2 (CH*C*H_3_), 19.8 (NCH_2_*C*H_2_).

**HRMS** (ESI-TOF) [M+H]^+^ calculated for C_22_H_29_N_2_O: 337.2274, found 337.2277.

### (*S*)-2-hexyl-4,4-dimethyl-2-{(*S*)-1-[(*R*)-1-phenylethyl]azetidin-2-yl}-1,3-oxazolidine 7a

According to the General Procedure, the reaction was carried out using dihydrooxazole (*R,S*)-**4b** (80 mg, 0.31 mmol) in dry toluene (3 mL) and hexyllithium (1.6 M in hexane, 290 μL, 0.46 mmol) affording **7a** as yellow oil, 96 mg, yield 90%, dr = 95:5, R_f_ 0.8 (30% AcOEt/hexane ${\text{[α]}}_{\text{D}}^{20}$ = −8.30° (*c* = 1, CHCl_3_).

**FT-IR** (film, cm^−1^) ν 3259, 2960, 2928, 2857, 1453, 1380, 1365, 1196, 1173, 1047, 930, 773, and 699.

^**1**^**H NMR** (500 MHz, CDCl_3_) δ 7.34–7.27 (m, 4H, Ar-H), 7.23–7.18 (m, 1H, Ar-H), 3.85 (q, *J* = 6.8 Hz, 1H, CHPh), 3.79 (t, *J* = 8.3 Hz, 1H, NCH), 3.53 (d, *J* = 7.6 Hz, 1H, OCH), 3.25 (d, *J* = 7.6 Hz, 1H, OCH), 3.19 (dd, *J* = 15.3, 8.6 Hz, 1H, NCH_2_), 2.93 (td, *J* = 7.9, 3.0 Hz, 1H, NCH_2_), 1.95–1.88 (m, 2H, NCH_2_C*H*_2_), 1.74–1.64 (m, 1H, hexyl-H), 1.51–1.42 (m, *J* = 13.0, 8.4 Hz, 1H, hexyl-H), 1.41–1.20 (m, 14H, CHC*H*_3_, oxazolidine-CH_3_ and 8 × hexyl-H), 1.19 (s, *J* = 8.0 Hz, 3H, oxazolidine-CH_3_), 0.88 (t, *J* = 6.9 Hz, 3H, hexyl-CH_3_).

^**13**^**C NMR** (126 MHz, CDCl_3_) δ 144.3 (Ar-C_q_), 128.2 (2 × Ar-C), 127.2 (2 × Ar-C), 126.6 (Ar-C), 100.7 (OC_q_NH), 77.1 (OCH_2_), 64.3 (NCH), 59.4 (oxazolidine-C_q_), 58.9 (CHPh), 42.9 (NCH_2_), 36.9 (hexyl-CH_2_), 31.9 (hexyl-CH_2_), 30.2 (hexyl-CH_2_), 29.6 (oxazolidine-CH_3_), 28.6 (oxazolidine-CH_3_), 24.2 (hexyl-CH_2_), 22.8 (hexyl-CH_2_), 20.6 (NCH_2_*C*H_2_), 14.2 (hexyl-CH_3_), 13.8 (CH*C*H_3_).

**HRMS** (ESI-TOF) [M+Na]^+^ calculated for C_22_H_36_N_2_NaO: 367.2725, found 367.2718.

### (*S*)-4,4-dimethyl-2-phenyl-2-{(*S*)-1-[(*R*)-1-phenylethyl]azetidin-2-yl}-1,3-oxazolidine 7d

According to the General Procedure, the reaction was carried out using dihydrooxazole (*R,S*)-**4b** (50 mg, 0.19 mmol) in dry toluene (3 mL) and phenyllithium (1.0 M in dibutyl ether, 290 μL, 0.29 mmol) affording oxazolidine **7b** as yellow oil, 59 mg, yield 90%, dr = 95:5). R_f_ 0.7 (30% AcOEt/hexane). ${\text{[α]}}_{\text{D}}^{20}$ = −3.09 (c = 0.5, CHCl_3_). (LUX-1, 99.5:0.5 Hex:iPrOH + 0.2% DEA, 0.5 mL/min).

**FT-IR** (film, cm^−1^) ν 3060, 3027, 2966, 2929, 2866, 1687, 1493, 1451, 1366, 1273, 1233, 1036, 742, and 700.

^**1**^**H NMR** (500 MHz, CDCl_3_) δ 7.59–7.55 (m, 2H, Ar-H), 7.39–7.21 (m, 8H, Ar-H overlapping CHCl_3_), 3.96 (q, *J* = 6.8 Hz, 1H, CHPh), 3.64 (t, *J* = 8.3 Hz, 1H, NCH), 3.32 (d, *J* = 7.3 Hz, 1H, OCH_2_), 3.16 (d, *J* = 7.3 Hz, 1H, OCH_2_), 3.06 (dt, *J* = 15.1, 7.5 Hz, 1H, NCH_2_), 2.97–2.90 (m, 1H, NCH_2_), 1.94 (tt, *J* = 17.4, 8.7 Hz, 1H, NCH_2_C*H*_2_), 1.47–1.36 (m, 4H, CHC*H*_3_ and NCH_2_C*H*_2_), 1.24 (s, 3H, oxazolidine-CH_3_), 0.86 (s, 3H, oxazolidine-CH_3_).

^**13**^**C NMR** (126 MHz, CDCl_3_) δ 144.4 (Ar-C_q_), 143.2 (Ar-C_q_), 128.9 (2 × Ar-C), 127.7 (2 × Ar-C), 127.5 (2 × Ar-C), 127.4 (Ar-C), 127.1 (2 × Ar-C), 126.6 (Ar-C_q_), 101.2 (OC_q_NH), 76.9 (OCH_2_) 67.3 (NCH), 59.9 (oxazolidine-C_q_), 59.8 (CHPh), 42.7 (NCH_2_), 28.5 (oxazolidine-CH_3_), 28.0 (oxazolidine-CH_3_), 20.2 (NCH_2_*C*H_2_), 14.6 (CH*C*H_3_).

**HRMS** (ESI-TOF) [M+Na]^+^ calculated for C_22_H_28_N_2_NaO: 359.2099, found 359.2092.

## Hydrolysis of 1,3-oxazolidines

General Procedure: To a solution of oxazolidines **5a-d**, **6a-c** and **7b** (0.16 mmol, 1 eq) in dichloromethane, silica (150 mg) was added. The reaction was stirred for 3 h at room temperature. The crude was filtered and the solvent was evaporated under reduced pressure to obtain the desire product.

1,3-Oxazolidines **7a** and **7c** undergo quantitative hydrolysis to corresponding acyl derivatives **10a** and **c** during the chromatography on silica gel.

### 1-(1-benzylazetidin-2-yl)pentan-1-one 8a

According to the General Procedure, the reaction was carried out using oxazolidine **5a** (50 mg, 0.16 mmol), ketoazetidine **8a** was obtained as colorless oil (33 mg, yield 90%).

**FT-IR** (film, cm^−1^) 2957, 2926, 2854, 1711, 1455, 1360, 1260, 1029, 913, 801, 747, and 700.

^**1**^**H NMR** (500 MHz, CDCl_3_): δ 7.33–7.23 (m, 5H, Ar-H overlapping CHCl_3_ signal), 3.73 (d, *J* = 12.5 Hz, 1H, CH_2_Ph), 3.68 (t, *J* = 8.6 Hz, 1H, azetidine-CH), 3.58 (d, *J* = 12.5 Hz, 1H, CH_2_Ph), 3.35–3.30 (m, 1H, azetidine-CH_2_), 2.94 (dd, *J* = 16.2, 8.0 Hz, 1H, azetidine-CH_2_), 2.53 (ddd, *J* = 17.5, 8.6, 6.3 Hz, 1H, butyl-CH_2_), 2.28-2.12 (m, 3H, azetidine-CH_2_ and butyl-CH_2_), 1.50–1.36 (m, 2H, butyl-CH_2_), 1.25-1.19 (m, 2H, butyl-CH_2_), 0.86 (t, *J* = 7.4 Hz, 3H, CH_3_).

^**13**^**C NMR** (126 MHz, CDCl_3_): δ 212.0 (C = O), 137.2 (Ar-C_q_), 129.0 (2 x Ar-C), 128.4 (2 x Ar-C), 127.3 (Ar-C), 71.33 (azetidine-CH), 62.9 (CH_2_Ph), 50.8 (azetidine-CH_2_), 37.7 (butyl-CH_2_), 25.1 (butyl-CH_2_), 22.3 (butyl-CH_2_), 21.8 (azetidine-CH_2_), 13.9 (CH_3_).

**HRMS** (ESI-TOF) [M+H]^+^ calculated for C_15_H_22_NONa: 254.1521, found 254.1510.

### 1-(1-benzylazetidin-2-yl)heptan-1-one 8b

According to the General Procedure, the reaction was carried out using from oxazolidine **5b** (50 mg, 0.15 mmol), ketoazetidine **8b** was obtained as colorless oil (35 mg, yield 90%).

**FT**-**IR** (film, cm^−1^) 2926, 2853, 1707, 1454, 1364, 1460, 1028, 912, 802, 735, and 699. ^1^H NMR (500 MHz, CDCl_3_): δ 7.36–7.22 (m, 5H, Ar-H overlapping CHCl_3_ signal), 3.73 (d, *J* = 12.6 Hz, 1H, CH_2_Ph), 3.68 (t, *J* = 8.7 Hz, 1H, azetidine-CH), 3.58 (d, *J* = 12.5 Hz, 1H, CH_2_Ph), 3.34–3.29 (m, 1H, azetidine-CH_2_), 2.94 (dd, *J* = 16.2, 8.0 Hz, 1H, azetidine-CH_2_), 2.53 (ddd, *J* = 17.5, 8.5, 6.3 Hz, 1H, hexyl-CH_2_), 2.28–2.11 (m, 3H, azetidine-CH_2_ and hexyl-CH_2_), 1.50–1.40 (m, 2H, hexyl-CH_2_), 1.30–1.17 (m, 6H, hexyl-CH_2_), 0.87 (t, *J* = 7.0 Hz, 3H, CH_3_).

^**13**^**C NMR** (126 MHz, CDCl_3_): δ 212.2 (C = O), 137.5 (Ar-C_q_), 129.2 (2 x Ar-C), 128.5 (2 x Ar-C), 127.5 (Ar-C), 71.5 (azetidine-CH), 63.1 (CH_2_Ph), 51.0 (azetidine-CH_2_), 31.8 (hexyl-CH_2_), 29.9 (hexyl-CH_2_), 29.1 (hexyl-CH_2_), 23.2 (hexyl-CH_2_), 22.7 (hexyl-CH_2_), 21.9 (azetidine-CH_2_), 14.2 (CH_3_).

**HRMS** (ESI-TOF) *m/z*: calcd for C_17_H_25_NONa [M+Na]^+^ 282.1834; found 282.1828.

### 1-(1-benzylazetidin-2-yl)ethanone 8c

According to the General Procedure, the reaction was carried out using oxazolidine **5c** (50 mg, 0.19 mmol) in dichloromethane (1 mL) affording 2-acylazetidine **8c** as yellow oil, yield 95%, 32 mg, dr = 95:5).

**FT-IR** (film, cm^−1^) 3086, 3062, 3028, 2965, 2930, 2867, 1955, 1881, 1734, 1658, 1652, 1531, 1454, 1162, 1069, and 914.

^**1**^**H NMR** (700 MHz, CDCl_3_) δ 7.33–7.23 (m, 5H, Ar-H overlapping CHCl_3_ signal), 3.73 (d, *J* = 12.6 Hz, 1H, CH_2_Ph), 3.66 (dd, *J* = 14.5, 5.8 Hz, 1H, azetidine-CH), 3.59 (d, *J* = 12.6 Hz, 1H, CH_2_Ph), 3.34–3.31 (m, 1H, azetidine-CH_2_), 2.94 (ddd, *J* = 9.3, 8.0, 6.9 Hz, 1H, azetidine-CH_2_), 2.23 (dtd, *J* = 10.8, 8.3, 2.5 Hz, 1H, azetidine-CH_2_), 2.18 (ddd, *J* = 19.4, 10.5, 8.9 Hz, 1H, azetidine-CH_2_), 2.05 (s, 3H, CH_3_).

^**13**^**C NMR** (176 MHz, CDCl_3_): δ 210.5 (C = O), 137.4 (Ar-C_q_), 129.1 (2 × Ar-C), 128.5 (2 × Ar-C), 127.5 (Ar-C), 71.8 (azetidine-CH), 63.2 (CH_2_Ph), 51.0 (azetidine-CH_2_), 25.7 (CH_3_), 21.8 (azetidine-CH_2_).

**HRMS** (ESI-TOF) [M+H]^+^ calculated for C_12_H_16_NO: 190.1232, found 190.1226.

### 1-(1-benzylazetidin-2-yl)propan-1-one 8d

According to the General Procedure, the reaction was carried out using oxazolidine **5d** (50 mg, 0.18 mmol), ketoazetidine **8d** was obtained as yellow oil (33 mg, yield 90%).

**FT-IR** (film, cm^−1^) 3063, 3029, 2964, 2928, 2875, 1955, 1709, 1634, 1495, 1454, 1404, 1241, 1077, 1028, 735, and 700.

^**1**^**H NMR** (500 MHz, CDCl_3_): δ 7.36–7.22 (m, 5H, Ar-H overlapping CHCl_3_ signal), 3.75 (d, *J* = 12.6 Hz, 1H, CH_2_Ph), 3.69 (t, *J* = 8.6 Hz, 1H, azetidine-CH), 3.57 (d, *J* = 12.6 Hz, 1H, CH_2_Ph), 3.33–3.28 (m, 1H, azetidine-CH_2_), 2.93 (q, *J* = 7.5 Hz, 1H, azetidine-CH_2_), 2.60 (dq, *J* = 18.4, 7.3 Hz, 1H, COCH_2_), 2.35–2.11 (m, 3H, azetidine-CH_2_ and COCH_2_), 0.95 (t, *J* = 7.3 Hz, 3H, CH_3_).

^**13**^**C NMR** (75 MHz, CDCl_3_): δ 212.6 (C = O), 137.6 (Ar-C_q_) 129.1 (2 x Ar-C), 128.5 (2 x Ar-C), 127.5 (Ar-C), 71.4 (azetidine-CH), 63.1 (CH_2_Ph), 51.1 (azetidine-CH_2_), 31.4 (CO*C*H_2_), 22.0 (azetidine-CH_2_), 7.33 (CH_3_).

**HRMS** (ESI-TOF) [M+H]^+^ calculated for C_13_H_18_NO: 204.1388, found 204.1381.

### 1-{(*R*)-1-[(*R*)-1-phenylethyl]azetidin-2-yl}pentan-1-one 9a

Starting from oxazolidine **6a** (50 mg, 0.16 mmol), ketoazetidine **9a** was obtained as pale yellow oil. (35 mg, yield 90%), er = 98:2 (LUX-1, 99:1 Hex:iPrOH + 0.5% DEA, 0.5mL/min). ${\text{[α]}}_{\text{D}}^{20}$ = + 149.46° (*c* = 1, CHCl_3_).

**FT-IR** (KBr, cm^−1^) ν 3391, 3028, 2960, 2930, 2871, 1705, 1493, 1453, 1371, 1282, 1029, 760, and 701.

^**1**^**H NMR** (500 MHz, CDCl_3_): δ 7.36–7.29 (m, 4H, Ar-H), 7.25–7.21 (m, 1H, Ar-H), 3.72 (t, *J* = 8.7 Hz, 1H, CHCO), 3.39 (q, *J* = 6.6 Hz, 1H, CHPh), 3.14–3.05 (m, 1H, NCH_2_), 2.80–2.60 (m, 3H, NC*H*H and COCH_2_), 2.21–2.12 (m, 1H, NCH_2_C*H*_2_ overlapping acetone), 2.10–2.00 (m, 1H, NCH_2_C*H*_2_), 1.66–1.54 (m, 2H, COCH_2_C*H*_2_), 1.40–1.31 (m, 2H, COCH_2_CH_2_C*H*_2_), 1.12 (d, *J* = 6.6 Hz, 3H, CHC*H*_3_), 0.94 (t, *J* = 7.4 Hz, 1H, CH_2_C*H*_3_).

^**13**^**C NMR** (126 MHz, CDCl_3_): δ 212.6 (CO), 143.1 (Ar-C_q_), 128.5 (2 x Ar-C), 127.4 (2 x Ar-C), 127.3 (Ar-C), 71.3 (*C*HCO), 67.7 (*C*HPh), 50.0 (NCH_2_), 37.4 (CO*C*H_2_), 25.7 (COCH_2_*C*H_2_), 22.6 (*C*H_2_CH_3_), 21.4 (*C*H_3_CH), 21.1 (NCH_2_*C*H_2_), 14.1 (CH_2_*C*H_3_).

**HRMS** (ESI-TOF) [M+H]^+^ calculated for C_16_H_24_NO: 246.1858, found 246.1852.

### 1-{(*R*)-1-[(*R*)-1-phenylethyl]azetidin-2-yl}heptan-1-one 9b

Starting from oxazolidine **6b** (50 mg, 0.145mmol), ketoazetidine **9b** was obtained as pale yellow oil (30 mg, yield 75%). ${\text{[α]}}_{\text{D}}^{20}$ = +102.22° (c = 0.7, CHCl_3_) (ADH, 99:1 Hex:iPrOH, 0.5 mL/min).

**FT-IR** (KBr, cm^−1^) ν 2958, 2928, 2854, 1706, 1493, 1452, 1370, 1282, 1068, 1029, 60, and 700.

^**1**^**H NMR** (500 MHz, CDCl_3_): δ 7.39–7.29 (m, 4H, Ar-H), 7.27–7.22 (m, 1H, Ar-H overlapping CHCl_3_), 3.72 (t, *J* = 8.6 Hz, 1H, NCH), 3.39 (q, *J* = 6.5 Hz, 1H, CHPh), 3.10 (dd, *J* = 11.0, 4.3 Hz, 1H, NCH), 2.86–2.58 (m, 3H, NC*H*H and COCH_2_), 2.22–2.11 (m, 1H, NCH_2_C*H*_2_), 2.10–2.01 (m, 1H, NCH_2_C*H*_2_), 1.64–1.54 (m, 2H, hexyl-CH_2_), 1.37–1.28 (m, 6H, hexyl-CH_2_), 1.12 (d, *J* = 6.6 Hz, 3H, CHC*H*_3_), 0.90 (t, *J* = 6.7 Hz, 3H, hexyl-CH_3_).

^**13**^**C NMR** (126 MHz, CDCl_3_): δ 212.7 (C = O), 143.1 (Ar-C_q_), 128.5 (2 × Ar-C), 127.5 (2 × Ar-C), 127.3 (Ar-C), 71.4 (NCH), 67.7 (CHPh), 50.0 (NCH_2_), 37.8 (hexyl-CH_2_), 31.9 (hexyl-CH_2_), 29.2 (hexyl-CH_2_), 23.5 (hexyl-CH_2_), 22.7 (hexyl-CH_2_), 21.5 (CH*C*H_3_), 21.1(NCH_2_*C*H_2_), 14.2 (hexyl-CH_3_).

**HRMS** calcd. for C_18_H_27_NNaO [M+Na]^+^ 296.1990; found 296.1978.

### 1-{(*R*)-1-[(*R*)-1-phenylethyl]azetidin-2-yl}ethanone 9c

Starting from oxazolidine **6c** (50 mg, 0.18 mmol), ketoazetidine **9c** was obtained as pale yellow oil (37 mg, yield 90%).

**FT-IR** (KBr, cm^−1^) ν 2968, 2929, 2850, 1705, 1493, 1453, 1354, 1282, 1245, 1172, 1072, 758, and 701.

^**1**^**H NMR** (500 MHz, CDCl_3_): δ 7.35–7.29 (m, 4H, Ar-H), 7.27–7.22 (m, 1H, Ar-H), 3.69 (t, *J* = 8.7 Hz, 1H, CHCO), 3.40 (q, *J* = 6.6 Hz, 1H, CHPh), 3.13–3.08 (m, 1H, NCH_2_), 2.78 (dd, *J* = 16.4, 8.0 Hz, 1H, NCH_2_), 2.34 (s, 3H, CH_3_CO), 2.22–2.02 (m, 2H, C*H*_2_CH), 1.13 (d, *J* = 6.6 Hz, 3H, CHC*H*_3_). ^**13**^**C NMR** (126 MHz, CDCl_3_): δ 211.2 (CO), 143.1 (Ar-C_q_), 128.5 (2 × Ar-C), 127.4 (2 × Ar-C), 127.3 (Ar-C), 71.6 (*C*HCO), 67.7 (*C*HPh), 50.0 (NCH_2_), 25.4 (*C*H_3_CO), 21.4 (*C*H_3_CH), 20.9 (NCH_2_*C*H_2_). **HRMS** (ESI-TOF) [M+Na]^+^ calculated for C_13_H_17_NNaO: 226.1208, found 226.1200.

### 1-{(*S*)-1-[(*R*)-1-phenylethyl]azetidin-2-yl}pentan-1-one 10a

Pale yellow oil. er = 98:2 (LUX-1, 99:1 Hex:iPrOH + 0.5% DEA, 0.5 mL/min). ${\text{[α]}}_{\text{D}}^{20}$ = −26.61° (*c* = 1, CHCl_3_).

**FT-IR** (KBr, cm^−1^) ν 2959, 2930, 2871, 1705, 1493, 1453, 1373, 1303, 1276, 1159, 1028, 760, and 701.

^**1**^**H NMR** (500 MHz, CDCl_3_): δ 7.26–7.17 (m, 5H, Ar-H), 3.63 (t, *J* = 8.6 Hz, 1H, CHCO), 3.52 (td, *J* = 7.3, 2.8 Hz, 1H, NCH_2_), 3.31 (q, *J* = 6.5 Hz, 1H, CHPh), 2.98 (dd, *J* = 16.0, 8.4 Hz, 1H, NCH_2_), 2.12–2.06 (m, 2H, NCH_2_C*H*_2_), 2.05–1.97 (m, 1H, COCH_2_), 1.92–1.83 (m, 1H, COCH_2_), 1.29 (d, *J* = 6.6 Hz, 3H, CHC*H*_3_), 1.22–0.98 (m, 4H, C*H*_2_C*H*_2_CH_3_), 0.76 (t, *J* = 7.2 Hz, 3H, CH_2_C*H*_3_).

^**13**^**C NMR** (126 MHz, CDCl_3_): δ 211.1 (CO), 142.0 (Ar-C_q_), 128.4 (2 × Ar-C), 128.4 (2 × Ar-C), 127.8 (Ar-C), 70.9 (*C*HCO), 68.5 (CHPh), 50.6 (NCH_2_), 38.3 (CO*C*H_2_), 25.3 (COCH_2_*C*H_2_), 22.3 (COCH_2_CH_2_*C*H_2_), 21.1 (NCH_2_*C*H_2_), 19.4 (CH*C*H_3_), 14.0 (CH_2_*C*H_3_).

**HRMS** (ESI-TOF) [M+H]^+^ calculated for C_16_H_24_NO: 246.1858, found 246.1852.

### 1-((*S*)-1-((*R*)-1-phenylethyl)azetidin-2-yl)heptan-1-one 10b

Starting from oxazolidine **7b** (50 mg, 0.145 mmol), ketoazetidine **10b** was obtained as pale yellow oil (36 mg, yield 90%), ${\text{[α]}}_{\text{D}}^{20}$ = −10.81° (*c* = 0.3, CHCl_3_). (LUX-1, 99:1 Hex:iPrOH +0.5% DEA, 0.5 mL/min).

**FT-IR** (KBr, cm^−1^) ν 2957, 2928, 2855, 1705, 1453, 1372, 1302, 1276, 1159, 1082, 1029, and 701.

^**1**^**H NMR** (500 MHz, CDCl_3_): δ 7.38–7.29 (m, 1H, Ar-H), 7.27–7.10 (m, 4H, 4 × Ar-H overlapping CDCl_3_), 3.63 (t, *J* = 8.5 Hz, 1H, NCH), 3.53 (td, *J* = 7.3, 2.7 Hz, 1H, NCH_2_), 3.32 (q, *J* = 6.6 Hz, 1H, CHPh), 2.99 (dd, *J* = 15.9, 8.6 Hz, 1H, NCH_2_), 2.13–1.97 (m, 3H, hexyl-H and NCH_2_), 1.88 (ddd, *J* = 17.5, 8.8, 5.9 Hz, 1H, hexyl-H), 1.33–1.08 (m, 9H, 6 × hexyl-H and CHC*H*_3_), 1.06–0.99 (m, 2H, hexyl-H), 0.85 (t, *J* = 7.3 Hz, 3H, hexyl-H).

^**13**^**C NMR** (126 MHz, CDCl_3_): δ 211.1 (C = O), 142.0 (Ar-C_q_), 128.4 (2 × Ar-C), 128.4 (2 × Ar-C), 127.8 (Ar-C), 71.0 (NCH), 68.5 (CHPh), 50.6 (NCH_2_), 38.6 (hexyl-C), 31.7 (hexyl-C), 28.9 (hexyl-C), 23.1 (hexyl-C), 22.6 (hexyl-C), 21.1 (NCH_2_*C*H_2_), 19.4 (CH*C*H_3_), 14.2 (hexyl-C).

**HRMS** (ESI-TOF) [M+H]^+^ calculated for C_18_H_27_NNaO: 296.1990, found 296.1985.

### 1-{(*S*)-1-[(*R*)-1-phenylethyl]azetidin-2-yl}ethanone 10c

Pale yellow oil. Yield 80%. ${\text{[α]}}_{\text{D}}^{20}$ = −3.52° (*c* = 0.3, CHCl_3_).

**FT-IR** (KBr, cm^−1^) ν 2965, 2928, 2851, 1706, 1493, 1453, 1352, 1244, 1167, 1030, and 702.

^**1**^**H NMR** (500 MHz, CDCl_3_): δ 7.35–7.30 (m, 1H, Ar-H), 7.28–7.20 (m, 4H, Ar-H overlapping CHCl_3_), 3.59 (t, *J* = 8.6 Hz, 1H, NCH), 3.52 (td, *J* = 7.4, 2.7 Hz, 1H, NCH_2_), 3.32 (q, *J* = 6.6 Hz, 1H, CHPh), 3.04–2.97 (m, 1H, NCH_2_), 2.15–2.02 (m, 2H, NCH_2_C*H*_2_), 1.69 (s, 3H, COCH_3_), 1.30 (d, *J* = 6.5 Hz, 3H, CHC*H*_3_).

^**13**^**C NMR** (126 MHz, CDCl_3_): δ 207.1 (C = O), 142.7 (Ar-C_q_), 128.5 (2 × Ar-C), 128.4 (2 × Ar-C), 128.0 (Ar-C), 71.5 (NCH), 68.3 (CHPh), 50.6 (NCH_2_), 25.5 (CO*C*H_3_), 20.8 (NCH_2_*C*H_2_), 19.2 (CH*C*H_3_).

**HRMS** (ESI-TOF) [M+Na]^+^ calculated for C_13_H_17_NNaO: 226.1208, found 226.1202.

### X-ray Structural Study of Compound (*S,S*)-3b

Colorless needles of compound (*S,S*)-**3b** were obtained by slow evaporation of solvent (Methanol) at room temperature. A single crystal (dimensions 0.500 × 0.350 × 0.220 mm) was selected and mounted on a glass fiber for the X-ray diffraction measurements. The X-ray diffraction experiment was carried out at room temperature by a Bruker-Nonius KappaCCD single crystal diffractometer, equipped with a charge-coupled device (CCD detector), using monochromatized MoKα radiation (λ 0.71073 Å). The automatic data collection was performed by the COLLECT software, cell determination and refinement by DIRAX and data reduction by EVAL. Absorption effects were corrected by SADABS program via a semi-empirical approach. Additional software used: WinGX21 for preparing the material for publication. The structure was solved by direct methods by using SIR2014 program and refined via full-matrix least squares on F2 by SHELXL2014/7. Non-hydrogen atoms were refined anisotropically. The hydrogen atoms were placed at calculated positions and refined isotropically using a riding model approximation with the displacement parameters set to Uiso(H) = 1.5·Ueq(C) in the case of methyl carbon and to Uiso(H) = 1.2·Ueq(C) for all other carbon atoms where Ueq is the equivalent isotropic displacement parameter of carbon. The compound (*S,S*)-**3b** belongs to the monoclinic crystal system with cell lengths (a 7.422(1); b 11.655(3); c 10.382(7); alpha 90; beta 110.40(1); gamma 90). Cell volume 841.8(2) Å3; Z 2. ρcalc 1.090 g/cm^3^; space group p21. A total of 3,806 reflections were collected at 298 K in the θ range from 3.410 to 27.489°, of which 2254 were observed (I > 2σ(I)). The final values of agreement factors were R 7.34 and wR 17.02% with a number of refined parameters was 187 and the maximum and minimum residual densities were Δρmax 0.52 and Δρmin −0.60. The complete crystallographic information on compound (*S,S*)-**3b** has been deposited at the Cambridge Crystallographic Data Center (deposit CCDC 1947700).

## Data Availability

All datasets generated for this study are included in the manuscript/Supplementary Files.

## Author Contributions

PM, MC, and FF have been involved in the synthesis of all compounds with the help of CC. AA and FF performed X-Ray analysis for compound (*S,S*)-**3b**. LP performed DFT and NMR calculations. RL and LD supervised this work and wrote the paper.

### Conflict of Interest Statement

The authors declare that the research was conducted in the absence of any commercial or financial relationships that could be construed as a potential conflict of interest.
