# Supplementary material for: Stereo- and Enantioselective Addition of Organolithiums to 2-Oxazolinylazetidines as a Synthetic Route to 2-Acylazetidines
Source: Front Chem. 2019 Sep 10;7:614. doi: 10.3389/fchem.2019.00614 (PMC6749145; doi:10.3389/fchem.2019.00614)
Supplement: Supplementary file 1 [file Data_Sheet_1.docx]

Stereo- and Enantioselective Addition of Organolithiums to 2-Oxazolinylazetidines as a Synthetic Route for Assessing 2-Acylazetidines

Pantaleo Musci,^1^ Marco Colella, ^1^ Flavio Fanelli,^1,2^ Angela Altomare,^2^ Luisa Pisano,^3^ Claudia Carlucci, ^1^ Renzo Luisi, ^1,*^ Leonardo Degennaro ^1,*^

^1^Flow Chemistry and Microreactor Technology FLAME-Lab, Department of Pharmacy–Drug Sciences, University of Bari “A. Moro” Via E. Orabona 4, 70125 Bari, Italy

^2^ CNR, Institute of Christallography IC-CNR, via Amendola 127/A, 70125 Bari, Italy

^3^Department of Chemistry and Pharmacy, University of Sassari, via Vienna 2, 07100 Sassari, Italy

*** Correspondence:**Leonardo Degennaro, [leonardo.degennaro@uniba.it](mailto:leonardo.degennaro@uniba.it)

Renzo Luisi, [renzo.luisi@uniba.it](mailto:renzo.luisi@uniba.it)

Table of Contents S1

^1^H of known esters **2a**, (*R*,*R*)-**2b**, (*R*,*S*)-**2b** S2

^1^H, ^13^C and NOESY NMR spectra for Isolated Compounds S4

X-ray structure and CIF of (*S,S*)-**3b** S56

DFT and GIAO calculations S57

**(*R,S*)-2b**

^1^H NMR (CDCl_3_, 500 MHz)

^1^H NMR (CDCl_3_, 500 MHz)

**(*R,R*)-2b**

^1^H NMR (CDCl_3_, 500 MHz)

^1^H NMR (CDCl_3_, 300 MHz)

**2a**

^1^H NMR (CDCl_3_, 500 MHz)

**3a**

^1^H NMR (CDCl_3_, 500 MHz)

**3a**

^13^C NMR (CDCl_3_, 126 MHz)

**4a**

^1^H NMR (CDCl_3_, 700 MHz)

**4a**

^13^C NMR (CDCl_3_, 126 MHz)

(*R*,*R*)-**3b**

^1^H NMR (CDCl_3_, 500 MHz)

(*R*,*R*)-**3b**

^13^C NMR (CDCl_3_, 126 MHz)

(*R*,*S*)-**3b**

^1^H NMR (CDCl_3_, 500 MHz)

(*R*,*S*)-**3b**

^13^C NMR (CDCl_3_, 126 MHz)

(*R*,*R*)-**4b**

^1^H NMR (CDCl_3_, 500 MHz)

(*R*,*R*)-**4b**

^13^C NMR (CDCl_3_, 126 MHz)

2 x CH_3_

All the interaction are depicted in the structure. Any interaction between phenylethyl group and oxazoline ring occurs. Then a trans arrangement is present

(*R*,*R*)-**4b**

H_E_/H_E’_

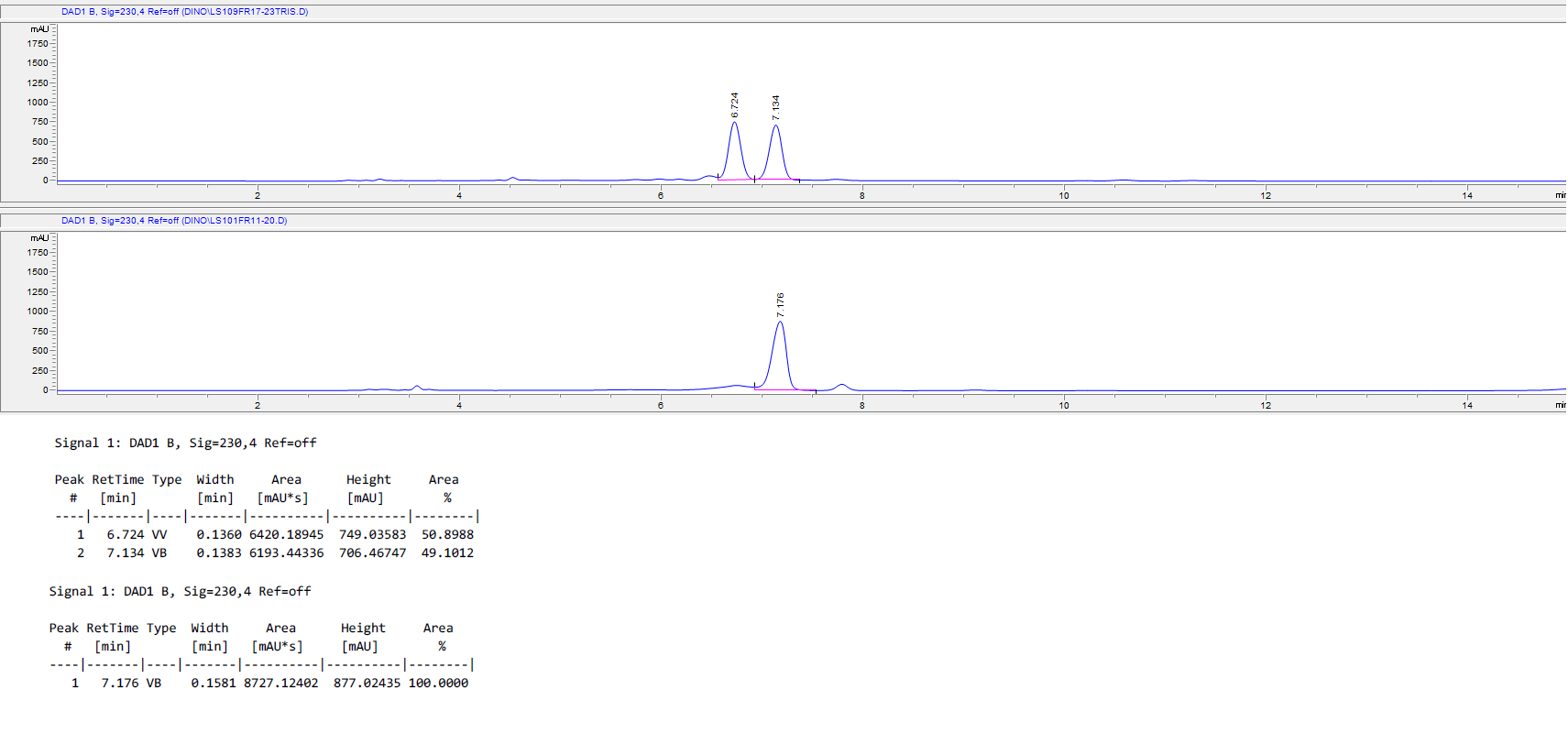

(*R*,*S*)-**4b**

^1^H NMR (CDCl_3_, 500 MHz)

(*R,S*)-**4b**

^13^C NMR (CDCl_3_, 126 MHz)

(*R*,*S*)-**4b**

All the interaction are depicted in the structure. Any interaction between phenylethyl group and oxazoline ring occurs. Then a trans arrangement is present

2 x CH_3_

H_E_/H_E’_

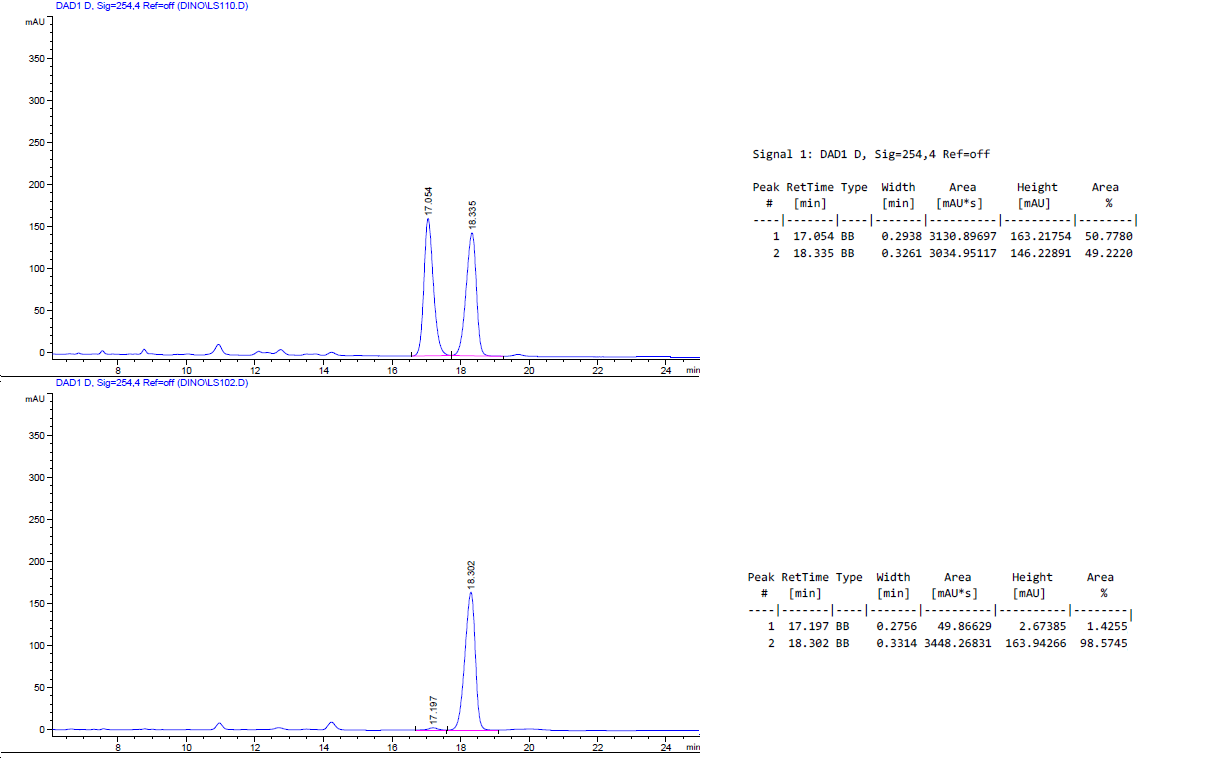

**5a**

^1^H NMR (CDCl_3_, 500 MHz)

**5a**

^13^C NMR (CDCl_3_, 176 MHz)

**5b**

^1^H NMR (CDCl_3_, 700 MHz)

**5b**

^13^C NMR (CDCl_3_, 126 MHz)

**5c**

^1^H NMR (CDCl_3_, 300 MHz)

**5c**

^13^C NMR (CDCl_3_, 126 MHz)

**5d**

^1^H NMR (CDCl_3_, 500 MHz)

**5d**

^13^C NMR (CDCl_3_, 126 MHz)

**5e**

^1^H NMR (CDCl_3_, 300 MHz)

**5e**

^13^C NMR (CDCl_3_, 176 MHz)

**6a**

^1^H NMR (CDCl_3_, 500 MHz)

**6a**

^13^C NMR (CDCl_3_, 126 MHz)

**6b**

^1^H NMR (CDCl_3_, 500 MHz)

**6b**

^13^C NMR (CDCl_3_, 126 MHz)

**6c**

^1^H NMR (CDCl_3_, 300 MHz)

**6c**

^13^C NMR (CDCl_3_, 126 MHz)

**6d**

^1^H NMR (CDCl_3_, 500 MHz)

**6d**

^13^C NMR (CDCl_3_, 126 MHz)


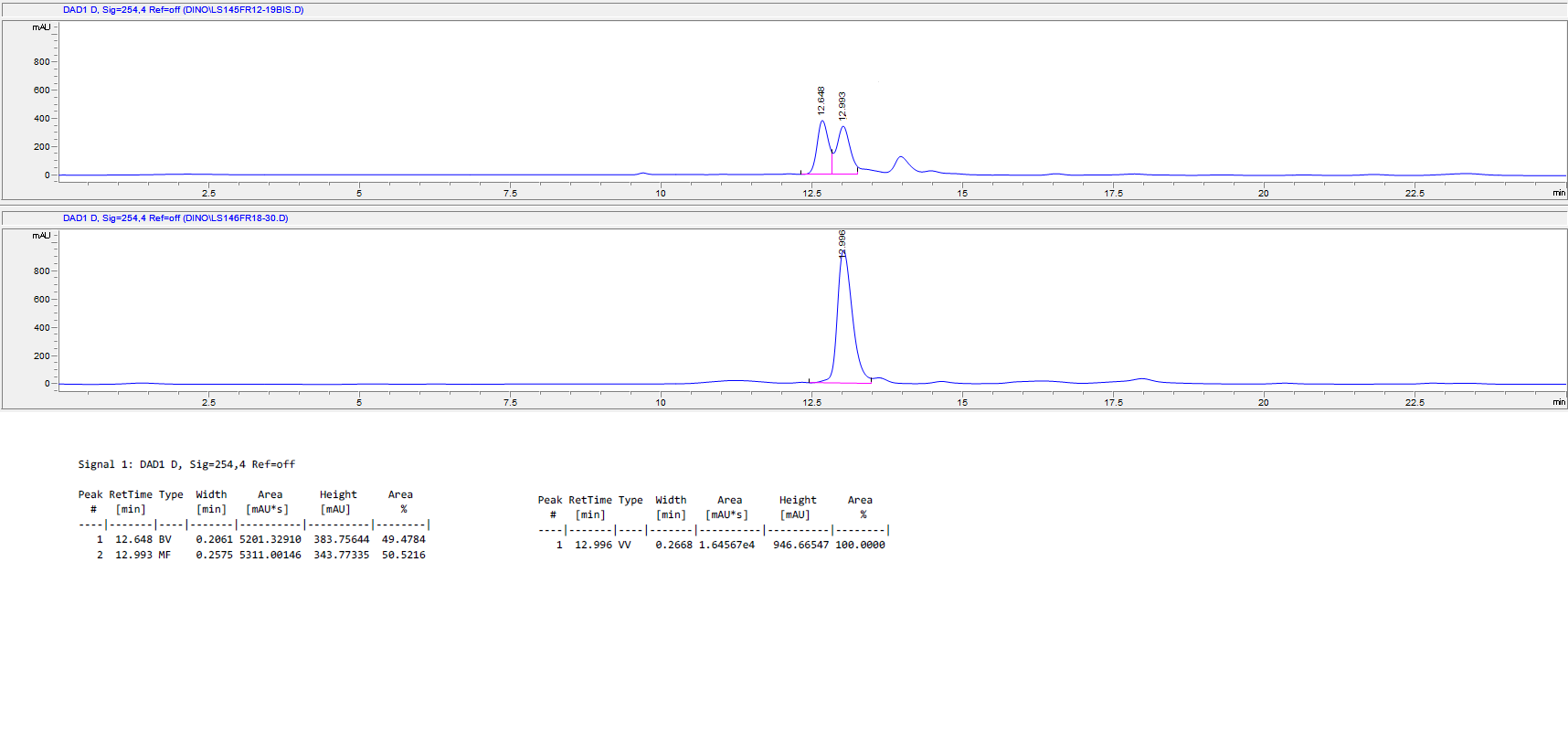

**7a**

^1^H NMR (CDCl_3_, 500 MHz)

**7a**

^13^C NMR (CDCl_3_, 126 MHz)

**7b**

^1^H NMR (CDCl_3_, 500 MHz)

**7b**

^13^C NMR (CDCl_3_, 126 MHz)


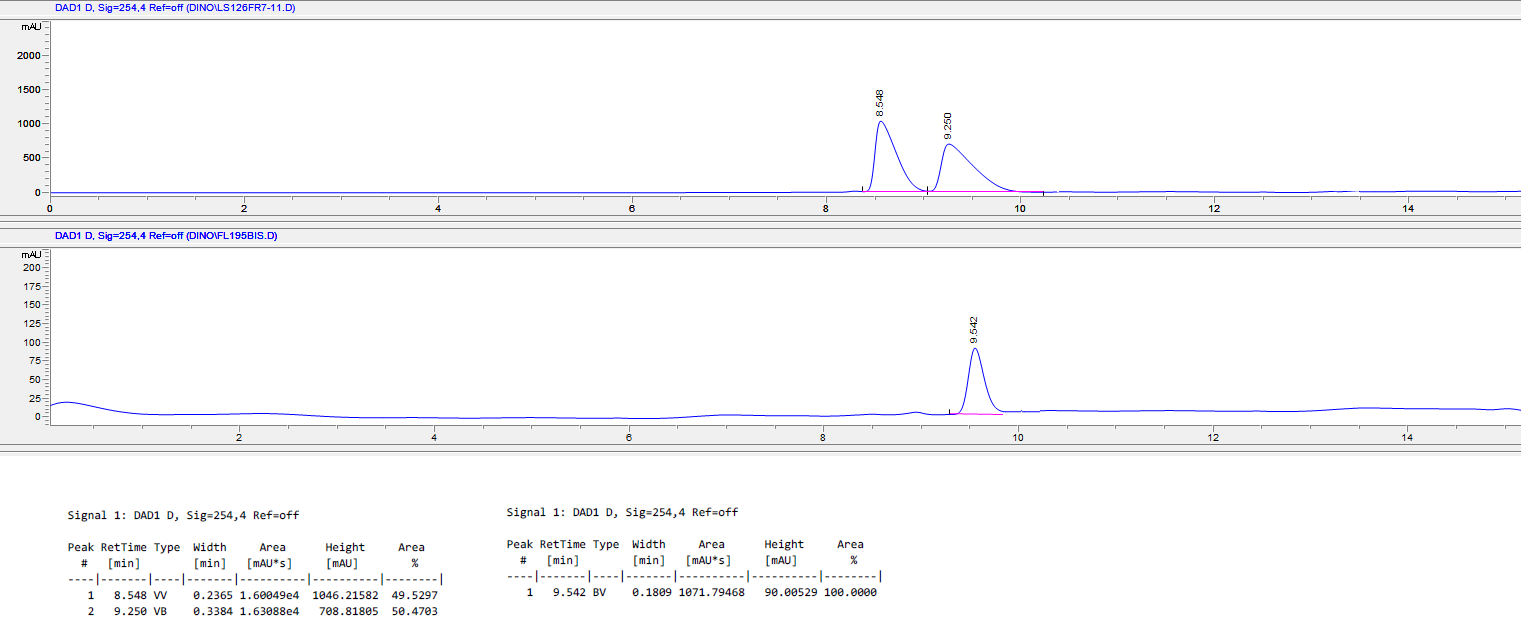

**8a**

^1^H NMR (CDCl_3_, 500 MHz)

**8a**

^13^C NMR (CDCl_3_, 126 MHz)

**8b**

^1^H NMR (CDCl_3_, 500 MHz)

**8b**

^13^C NMR (CDCl_3_, 126 MHz)

**8c**

^1^H NMR (CDCl_3_, 700 MHz)

**8c**

^13^C NMR (CDCl_3_, 176 MHz)

**8d**

^1^H NMR (CDCl_3_, 500 MHz)

**8d**

^13^C NMR (CDCl_3_, 75 MHz)

**9a**

^1^H NMR (CDCl_3_, 500 MHz)

**9a**

^13^C NMR (CDCl_3_, 126 MHz)


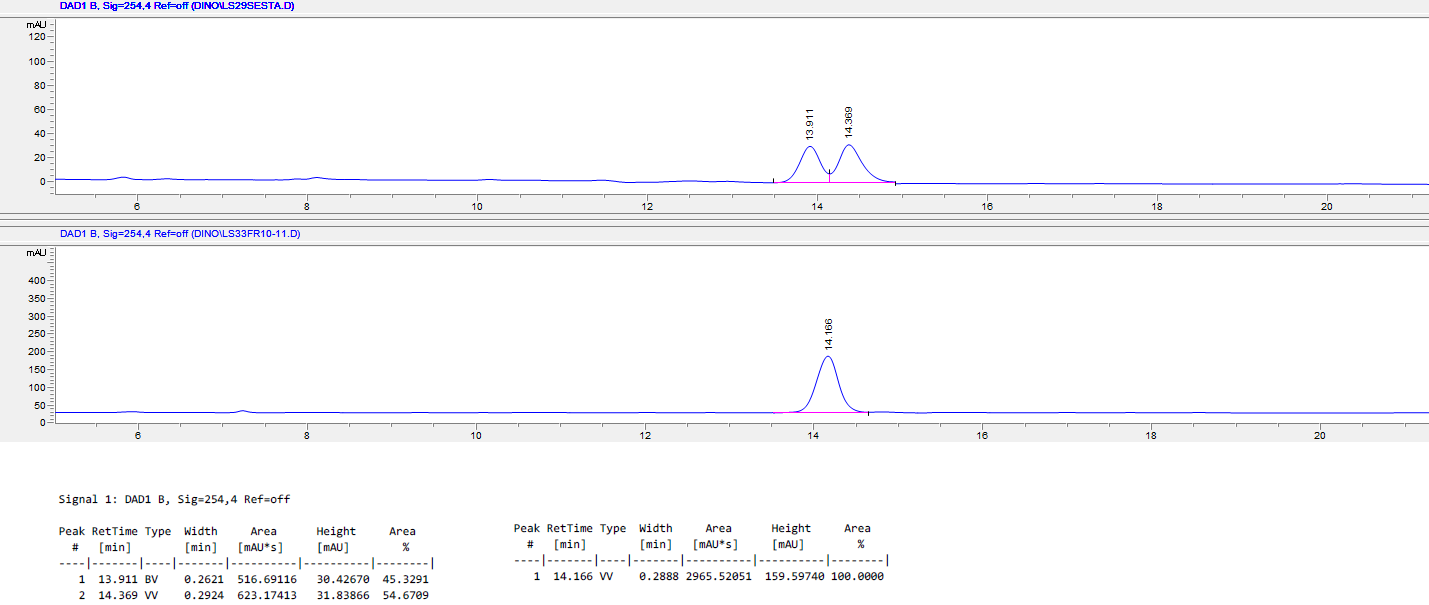


**9b**

^1^H NMR (CDCl_3_, 500 MHz)

**9b**

^13^C NMR (CDCl_3_, 126 MHz)

**9c**

^1^H NMR (CDCl_3_, 500 MHz)

**9c**

^13^C NMR (CDCl_3_, 126 MHz)

**10a**

^1^H NMR (CDCl_3_, 500 MHz)

**10a**

^13^C NMR (CDCl_3_, 126 MHz)


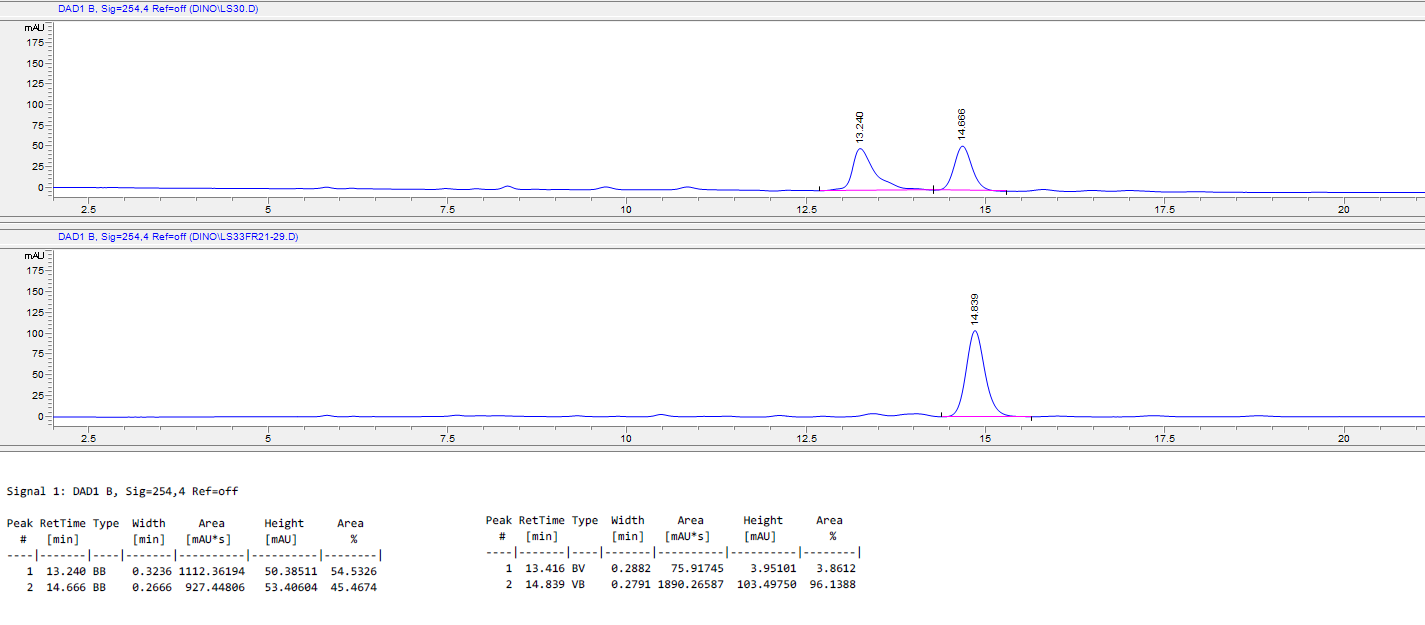


**10b**

^1^H NMR (CDCl_3_, 500 MHz)

**10b**

^13^C NMR (CDCl_3_, 126 MHz)


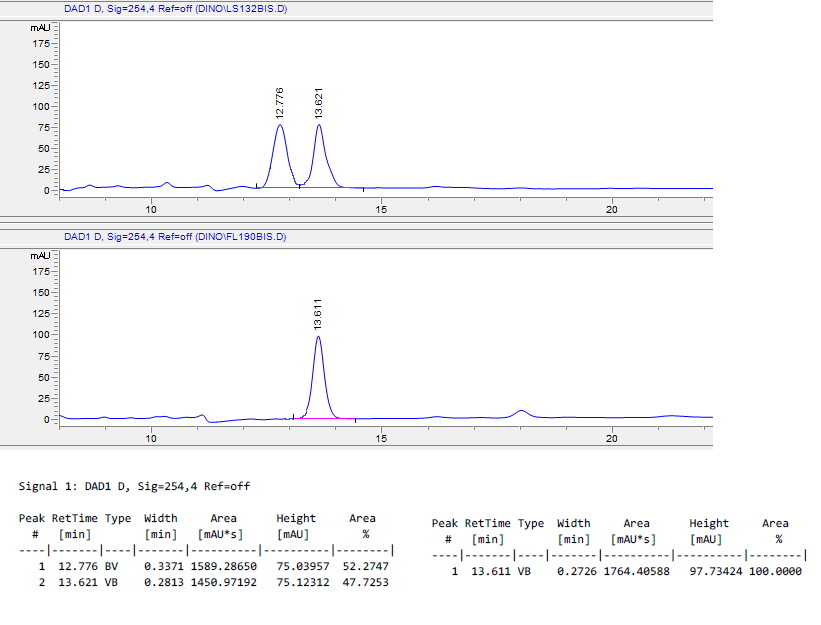


**10c**

^1^H NMR (CDCl_3_, 500 MHz)

**10c**

^13^C NMR (CDCl_3_, 126 MHz)

X-ray structure of (*S,S*)-**3b**

---------------------------------------------------------------
Summary of Data - Deposition Number 1947700
---------------------------------------------------------------
Compound Name: **(*S*)-*N*-(1-hydroxy-2-methylpropan-2-yl)-1-[(*S*)-1-phenylethyl]azetidine-2-carboxamide**
Data Block Name: data_shelx
Unit Cell Parameters: a 7.422(1) b 11.655(3) c 10.382(7) P21
---------------------------------------------------------------


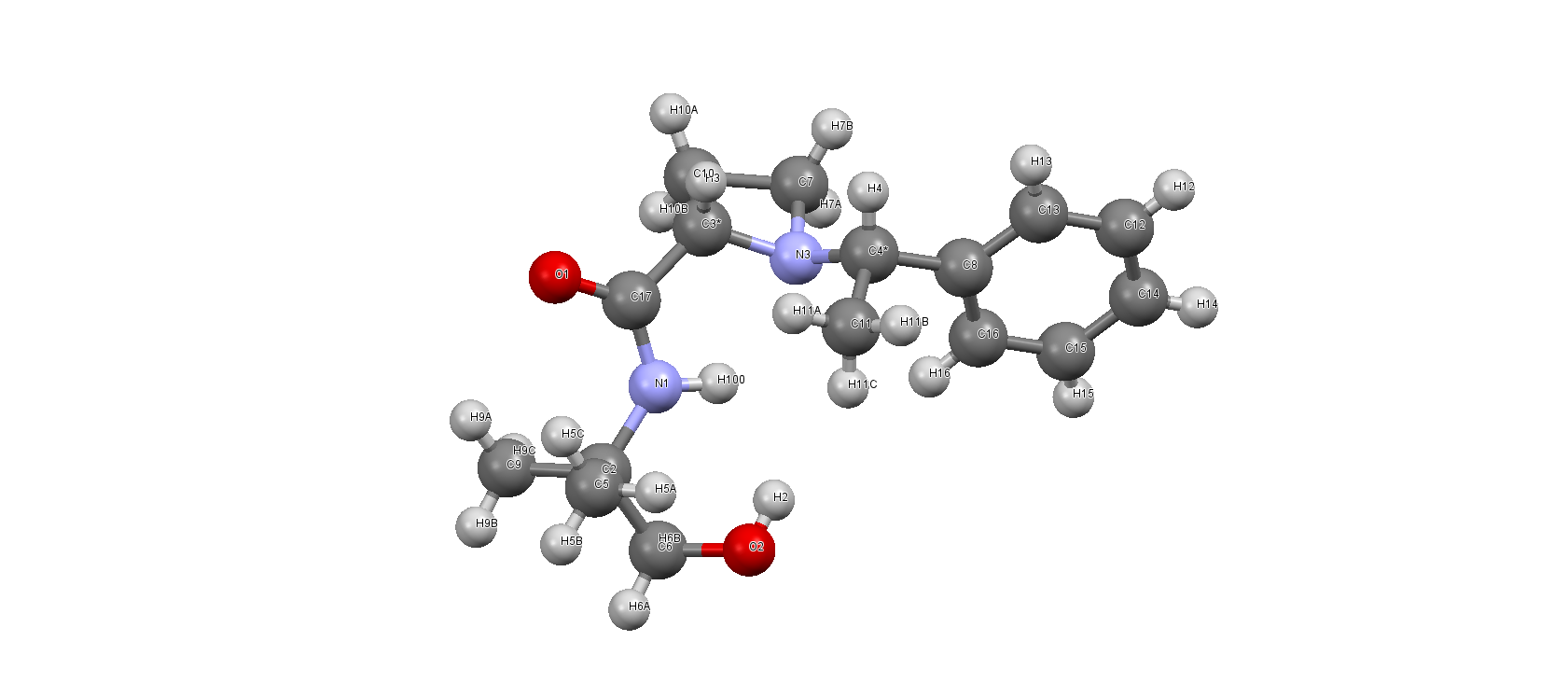


data_shelx

_audit_creation_method 'SHELXL-2014/7'

_shelx_SHELXL_version_number '2014/7'

_chemical_formula_sum

'C16 H24 N2 O2'

_chemical_formula_weight 276.37

loop_

_atom_type_symbol

_atom_type_description

_atom_type_scat_dispersion_real

_atom_type_scat_dispersion_imag

_atom_type_scat_source

'C' 'C' 0.0033 0.0016

'International Tables Vol C Tables 4.2.6.8 and 6.1.1.4'

'H' 'H' 0.0000 0.0000

'International Tables Vol C Tables 4.2.6.8 and 6.1.1.4'

'N' 'N' 0.0061 0.0033

'International Tables Vol C Tables 4.2.6.8 and 6.1.1.4'

'O' 'O' 0.0106 0.0060

'International Tables Vol C Tables 4.2.6.8 and 6.1.1.4'

_space_group_crystal_system monoclinic

_space_group_IT_number 4

_space_group_name_H-M_alt 'P 21'

_space_group_name_Hall 'P 2yb'

_shelx_space_group_comment

;

The symmetry employed for this shelxl refinement is uniquely defined by the following loop, which should always be used as a source of symmetry information in preference to the above space-group names.

They are only intended as comments.

loop_

_space_group_symop_operation_xyz

'x, y, z'

'-x, y+1/2, -z'

_cell_length_a 7.422(1)

_cell_length_b 11.655(3)

_cell_length_c 10.382(7)

_cell_angle_alpha 90

_cell_angle_beta 110.40(1)

_cell_angle_gamma 90

_cell_volume 841.8(2)

_cell_formula_units_Z 2

_cell_measurement_temperature 293(2)

_cell_measurement_reflns_used 216

_cell_measurement_theta_min 3

_cell_measurement_theta_max 25

_exptl_crystal_description block

_exptl_crystal_colour colourless

_exptl_crystal_density_meas ?

_exptl_crystal_density_method ?

_exptl_crystal_density_diffrn 1.090

_exptl_crystal_F_000 300

_exptl_transmission_factor_min ?

_exptl_transmission_factor_max ?

_exptl_crystal_size_max 0.500

_exptl_crystal_size_mid 0.350

_exptl_crystal_size_min 0.220

_exptl_absorpt_coefficient_mu 0.072

_shelx_estimated_absorpt_T_min 0.906

_shelx_estimated_absorpt_T_max 0.990

_exptl_absorpt_correction_type 'multi-scan'

_exptl_absorpt_correction_T_min 0.808

_exptl_absorpt_correction_T_max 0.879

_exptl_absorpt_process_details 'SADABS(Sheldrick, 2008)'

_exptl_absorpt_special_details ?

_diffrn_ambient_temperature 293(2)

_diffrn_radiation_wavelength 0.71073

_diffrn_radiation_type MoK\a

_diffrn_source 'fine-focus sealed tube'

_diffrn_measurement_device_type 'Bruker-Nonius KappaCCD'

_diffrn_measurement_method '\f scans and \w scans'

_diffrn_detector_area_resol_mean ?

_diffrn_reflns_number 13613

_diffrn_reflns_av_unetI/netI 0.0691

_diffrn_reflns_av_R_equivalents 0.0770

_diffrn_reflns_limit_h_min -9

_diffrn_reflns_limit_h_max 9

_diffrn_reflns_limit_k_min -15

_diffrn_reflns_limit_k_max 14

_diffrn_reflns_limit_l_min -13

_diffrn_reflns_limit_l_max 13

_diffrn_reflns_theta_min 3.410

_diffrn_reflns_theta_max 27.489

_diffrn_reflns_theta_full 25.242

_diffrn_measured_fraction_theta_max 0.996

_diffrn_measured_fraction_theta_full 0.995

_diffrn_reflns_Laue_measured_fraction_max 0.996

_diffrn_reflns_Laue_measured_fraction_full 0.995

_diffrn_reflns_point_group_measured_fraction_max 0.982

_diffrn_reflns_point_group_measured_fraction_full 0.989

_reflns_number_total 3806

_reflns_number_gt 2254

_reflns_threshold_expression 'I > 2\s(I)'

_reflns_Friedel_coverage 0.880

_reflns_Friedel_fraction_max 0.967

_reflns_Friedel_fraction_full 0.981

_reflns_special_details

Reflections were merged by SHELXL according to the crystal class for the calculation of statistics and refinement.

_reflns_Friedel_fraction is defined as the number of unique Friedel pairs measured divided by the number that would be possible theoretically, ignoring centric projections and systematic absences.

_computing_data_collection 'COLLECT (Nonius, 2002)'

_computing_cell_refinement

'DIRAX (Duisenberg,1992; Duisenberg et al., 2003)'

_computing_data_reduction

'EVAL (Nonius, 2002; Duisenberg et al., 2000)'

_computing_structure_solution 'SIR2014 (Burla et al., 2015)'

_computing_structure_refinement 'SHELXL-2014/7 (Sheldrick, 2014)'

_computing_molecular_graphics 'ORTEP-3 (Farrugia, 2012)'

_computing_publication_material

'WinGX (Farrugia, 2012) and publCIF (Westrip, 2010)'

_refine_special_details ?

_refine_ls_structure_factor_coef Fsqd

_refine_ls_matrix_type full

_refine_ls_weighting_scheme calc

_refine_ls_weighting_details

'w=1/[\s^2^(Fo^2^)+(0.1103P)^2^] where P=(Fo^2^+2Fc^2^)/3'

_atom_sites_solution_primary ?

_atom_sites_solution_secondary ?

_atom_sites_solution_hydrogens mixed

_refine_ls_hydrogen_treatment mixed

_refine_ls_extinction_method none

_refine_ls_extinction_coef .

_refine_ls_abs_structure_details

;

Flack x determined using 775 quotients [(I+)-(I-)]/[(I+)+(I-)]

(Parsons, Flack and Wagner, Acta Cryst. B69 (2013) 249-259).

;

_refine_ls_abs_structure_Flack 1.3(10)

_chemical_absolute_configuration 'rm'

_refine_ls_number_reflns 3806

_refine_ls_number_parameters 187

_refine_ls_number_restraints 1

_refine_ls_R_factor_all 0.1247

_refine_ls_R_factor_gt 0.0734

_refine_ls_wR_factor_ref 0.2067

_refine_ls_wR_factor_gt 0.1702

_refine_ls_goodness_of_fit_ref 1.091

_refine_ls_restrained_S_all 1.091

_refine_ls_shift/su_max 0.000

_refine_ls_shift/su_mean 0.000

loop_

_atom_site_label

_atom_site_type_symbol

_atom_site_fract_x

_atom_site_fract_y

_atom_site_fract_z

_atom_site_U_iso_or_equiv

_atom_site_adp_type

_atom_site_occupancy

_atom_site_site_symmetry_order

_atom_site_calc_flag

_atom_site_refinement_flags_posn

_atom_site_refinement_flags_adp

_atom_site_refinement_flags_occupancy

_atom_site_disorder_assembly

_atom_site_disorder_group

O1 O 0.5673(4) 0.7297(3) 0.0190(4) 0.0737(11) Uani 1 1 d . . . . .

O2 O 1.2412(5) 0.7650(5) 0.0708(4) 0.0948(15) Uani 1 1 d . . . . .

H2 H 1.2086 0.8012 0.1267 0.142 Uiso 1 1 calc R U . . .

N1 N 0.8699(5) 0.7923(3) 0.0508(4) 0.0515(9) Uani 1 1 d . . . . .

N3 N 0.9034(5) 0.9039(3) 0.2936(4) 0.0530(9) Uani 1 1 d . . . . .

C17 C 0.7139(6) 0.7850(4) 0.0843(5) 0.0547(10) Uani 1 1 d . . . . .

C2 C 0.9097(6) 0.7309(4) -0.0603(4) 0.0534(10) Uani 1 1 d . . . . .

C3 C 0.7184(6) 0.8533(4) 0.2075(5) 0.0586(12) Uani 1 1 d . . . . .

H3 H 0.6629 0.8087 0.2645 0.070 Uiso 1 1 calc R U . . .

C4 C 1.0116(8) 0.8450(5) 0.4214(5) 0.0683(13) Uani 1 1 d . . . . .

H4 H 0.9246 0.8290 0.4718 0.082 Uiso 1 1 calc R U . . .

C5 C 0.9246(8) 0.6024(5) -0.0290(7) 0.0747(14) Uani 1 1 d . . . . .

H5A H 1.0250 0.5890 0.0576 0.112 Uiso 1 1 calc R U . . .

H5B H 0.9536 0.5622 -0.1002 0.112 Uiso 1 1 calc R U . . .

H5C H 0.8047 0.5752 -0.0249 0.112 Uiso 1 1 calc R U . . .

C6 C 1.1020(7) 0.7770(5) -0.0593(5) 0.0642(12) Uani 1 1 d . . . . .

H6A H 1.1415 0.7354 -0.1259 0.077 Uiso 1 1 calc R U . . .

H6B H 1.0888 0.8573 -0.0852 0.077 Uiso 1 1 calc R U . . .

C7 C 0.8021(8) 1.0110(5) 0.3065(6) 0.0736(15) Uani 1 1 d . . . . .

H7A H 0.8635 1.0805 0.2911 0.088 Uiso 1 1 calc R U . . .

H7B H 0.7745 1.0160 0.3911 0.088 Uiso 1 1 calc R U . . .

C8 C 1.1719(8) 0.9217(5) 0.5091(5) 0.0658(13) Uani 1 1 d . . . . .

C9 C 0.7572(8) 0.7560(6) -0.1986(5) 0.0849(18) Uani 1 1 d . . . . .

H9A H 0.6364 0.7248 -0.2009 0.127 Uiso 1 1 calc R U . . .

H9B H 0.7934 0.7216 -0.2701 0.127 Uiso 1 1 calc R U . . .

H9C H 0.7451 0.8375 -0.2125 0.127 Uiso 1 1 calc R U . . .

C10 C 0.6305(9) 0.9730(5) 0.1823(6) 0.0822(17) Uani 1 1 d . . . . .

H10A H 0.5075 0.9790 0.1951 0.099 Uiso 1 1 calc R U . . .

H10B H 0.6260 1.0072 0.0961 0.099 Uiso 1 1 calc R U . . .

C11 C 1.0892(11) 0.7313(5) 0.3897(7) 0.098(2) Uani 1 1 d . . . . .

H11A H 0.9838 0.6825 0.3399 0.148 Uiso 1 1 calc R U . . .

H11B H 1.1646 0.6947 0.4741 0.148 Uiso 1 1 calc R U . . .

H11C H 1.1680 0.7454 0.3351 0.148 Uiso 1 1 calc R U . . .

C12 C 1.3604(13) 1.0012(8) 0.7276(6) 0.105(2) Uani 1 1 d . . . . .

H12 H 1.3830 1.0076 0.8212 0.126 Uiso 1 1 calc R U . . .

C13 C 1.2114(11) 0.9351(6) 0.6472(6) 0.0881(19) Uani 1 1 d . . . . .

H13 H 1.1340 0.8979 0.6878 0.106 Uiso 1 1 calc R U . . .

C14 C 1.4750(10) 1.0573(7) 0.6724(8) 0.102(2) Uani 1 1 d . . . . .

H14 H 1.5780 1.1005 0.7281 0.122 Uiso 1 1 calc R U . . .

C15 C 1.4393(11) 1.0503(10) 0.5349(8) 0.121(3) Uani 1 1 d . . . . .

H15 H 1.5133 1.0918 0.4951 0.145 Uiso 1 1 calc R U . . .

C16 C 1.2895(10) 0.9799(8) 0.4540(6) 0.104(2) Uani 1 1 d . . . . .

H16 H 1.2689 0.9724 0.3608 0.125 Uiso 1 1 calc R U . . .

H100 H 0.959(14) 0.843(10) 0.118(10) 0.156 Uiso 1 1 d . U . . .

loop_

_atom_site_aniso_label

_atom_site_aniso_U_11

_atom_site_aniso_U_22

_atom_site_aniso_U_33

_atom_site_aniso_U_23

_atom_site_aniso_U_13

_atom_site_aniso_U_12

O1 0.0426(16) 0.095(3) 0.087(2) -0.024(2) 0.0280(16) -0.0128(18)

O2 0.0467(17) 0.164(5) 0.079(2) -0.016(3) 0.0280(17) -0.013(2)

N1 0.0442(17) 0.053(2) 0.064(2) -0.0093(17) 0.0270(16) -0.0012(16)

N3 0.055(2) 0.053(2) 0.0542(19) 0.0004(17) 0.0221(17) 0.0018(17)

C17 0.042(2) 0.057(2) 0.069(3) -0.003(2) 0.024(2) 0.002(2)

C2 0.048(2) 0.056(2) 0.058(2) -0.012(2) 0.0204(18) 0.001(2)

C3 0.050(2) 0.065(3) 0.068(3) -0.008(2) 0.031(2) -0.001(2)

C4 0.076(3) 0.070(3) 0.063(3) 0.012(2) 0.030(3) -0.004(3)

C5 0.076(3) 0.055(3) 0.102(4) -0.010(3) 0.041(3) -0.002(3)

C6 0.062(3) 0.071(3) 0.073(3) -0.004(2) 0.040(2) 0.002(2)

C7 0.083(4) 0.065(3) 0.078(3) -0.012(3) 0.035(3) 0.008(3)

C8 0.072(3) 0.074(3) 0.051(2) 0.007(2) 0.020(2) 0.003(3)

C9 0.076(3) 0.109(5) 0.066(3) -0.009(3) 0.019(3) 0.014(3)

C10 0.070(3) 0.086(4) 0.090(4) -0.015(3) 0.027(3) 0.024(3)

C11 0.120(5) 0.061(3) 0.096(4) 0.017(3) 0.015(4) 0.019(4)

C12 0.117(6) 0.126(6) 0.056(3) -0.011(4) 0.011(4) 0.024(5)

C13 0.108(5) 0.104(5) 0.053(3) 0.006(3) 0.029(3) 0.003(4)

C14 0.071(4) 0.118(6) 0.091(4) -0.024(4) -0.005(3) 0.004(4)

C15 0.085(5) 0.177(9) 0.095(5) -0.025(5) 0.024(4) -0.054(5)

C16 0.084(4) 0.162(7) 0.065(3) -0.004(4) 0.024(3) -0.031(5)

_geom_special_details;

All esds (except the esd in the dihedral angle between two l.s. planes)

are estimated using the full covariance matrix. The cell esds are taken

into account individually in the estimation of esds in distances, angles

and torsion angles; correlations between esds in cell parameters are only

used when they are defined by crystal symmetry. An approximate (isotropic)

treatment of cell esds is used for estimating esds involving l.s. planes;

loop_

_geom_bond_atom_site_label_1

_geom_bond_atom_site_label_2

_geom_bond_distance

_geom_bond_site_symmetry_2

_geom_bond_publ_flag

O1 C17 1.243(5) . ?

O2 C6 1.392(6) . ?

N1 C17 1.323(5) . ?

N1 C2 1.473(5) . ?

N3 C4 1.461(6) . ?

N3 C3 1.475(6) . ?

N3 C7 1.487(7) . ?

C17 C3 1.497(6) . ?

C2 C9 1.515(7) . ?

C2 C6 1.521(6) . ?

C2 C5 1.528(7) . ?

C3 C10 1.523(7) . ?

C4 C8 1.513(8) . ?

C4 C11 1.526(9) . ?

C7 C10 1.529(8) . ?

C8 C13 1.368(7) . ?

C8 C16 1.377(9) . ?

C12 C14 1.348(12) . ?

C12 C13 1.368(11) . ?

C14 C15 1.361(11) . ?

C15 C16 1.401(10) . ?

loop_

_geom_angle_atom_site_label_1

_geom_angle_atom_site_label_2

_geom_angle_atom_site_label_3

_geom_angle

_geom_angle_site_symmetry_1

_geom_angle_site_symmetry_3

_geom_angle_publ_flag

C17 N1 C2 127.4(4) . . ?

C4 N3 C3 117.6(4) . . ?

C4 N3 C7 116.6(4) . . ?

C3 N3 C7 89.8(3) . . ?

O1 C17 N1 124.7(4) . . ?

O1 C17 C3 119.8(3) . . ?

N1 C17 C3 115.4(4) . . ?

N1 C2 C9 111.2(4) . . ?

N1 C2 C6 105.5(4) . . ?

C9 C2 C6 109.1(4) . . ?

N1 C2 C5 109.2(4) . . ?

C9 C2 C5 111.4(4) . . ?

C6 C2 C5 110.2(4) . . ?

N3 C3 C17 118.0(3) . . ?

N3 C3 C10 89.5(4) . . ?

C17 C3 C10 117.3(5) . . ?

N3 C4 C8 109.7(4) . . ?

N3 C4 C11 109.9(4) . . ?

C8 C4 C11 111.5(5) . . ?

O2 C6 C2 110.4(4) . . ?

N3 C7 C10 88.8(4) . . ?

C13 C8 C16 116.3(6) . . ?

C13 C8 C4 122.3(5) . . ?

C16 C8 C4 121.4(4) . . ?

C3 C10 C7 86.5(4) . . ?

C14 C12 C13 120.6(6) . . ?

C12 C13 C8 122.4(7) . . ?

C12 C14 C15 119.7(7) . . ?

C14 C15 C16 119.2(8) . . ?

C8 C16 C15 121.6(6) . . ?

_refine_diff_density_max 0.522

_refine_diff_density_min -0.602

_refine_diff_density_rms 0.198

_shelx_res_file;

shelx.res created by SHELXL-2014/7

TITL fl_hd-1

CELL 0.71073 7.4223 11.6550 10.3824 90.000 110.397 90.000

ZERR 2.00 0.0000 0.0000 0.0000 0.000 0.000 0.000

LATT -1

SYMM - X, 1/2 + Y, - Z

SFAC C H N O

UNIT 32 48 4 4

MERG 2

FMAP 2

PLAN 50 0.50 0.50

ACTA

L.S. 8

WGHT 0.110300

FVAR 1.98353

O1 4 0.567330 0.729716 0.018956 11.00000 0.04257 0.09544 =

0.08728 -0.02363 0.02803 -0.01280

O2 4 1.241189 0.765012 0.070775 11.00000 0.04673 0.16365 =

0.07881 -0.01644 0.02797 -0.01304

AFIX 83

H2 2 1.208596 0.801239 0.126740 11.00000 -1.50000

AFIX 0

N1 3 0.869865 0.792327 0.050834 11.00000 0.04423 0.05302 =

0.06362 -0.00933 0.02698 -0.00123

N3 3 0.903444 0.903920 0.293589 11.00000 0.05490 0.05256 =

0.05417 0.00039 0.02214 0.00181

C17 1 0.713866 0.784997 0.084304 11.00000 0.04157 0.05697 =

0.06929 -0.00330 0.02409 0.00172

C2 1 0.909713 0.730891 -0.060338 11.00000 0.04757 0.05568 =

0.05834 -0.01246 0.02036 0.00129

C3 1 0.718427 0.853305 0.207531 11.00000 0.05041 0.06514 =

0.06843 -0.00779 0.03112 -0.00088

AFIX 13

H3 2 0.662937 0.808682 0.264541 11.00000 -1.20000

AFIX 0

C4 1 1.011639 0.845003 0.421441 11.00000 0.07616 0.06978 =

0.06333 0.01188 0.02970 -0.00370

AFIX 13

H4 2 0.924603 0.828975 0.471759 11.00000 -1.20000

AFIX 0

C5 1 0.924621 0.602403 -0.029032 11.00000 0.07577 0.05469 =

0.10155 -0.00952 0.04097 -0.00198

AFIX 37

H5A 2 1.024985 0.589041 0.057634 11.00000 -1.50000

H5B 2 0.953580 0.562230 -0.100171 11.00000 -1.50000

H5C 2 0.804664 0.575224 -0.024894 11.00000 -1.50000

AFIX 0

C6 1 1.101978 0.776951 -0.059284 11.00000 0.06160 0.07083 =

0.07341 -0.00413 0.04013 0.00241

AFIX 23

H6A 2 1.141536 0.735375 -0.125899 11.00000 -1.20000

H6B 2 1.088759 0.857309 -0.085224 11.00000 -1.20000

AFIX 0

C7 1 0.802100 1.011021 0.306540 11.00000 0.08332 0.06516 =

0.07823 -0.01171 0.03546 0.00795

AFIX 23

H7A 2 0.863543 1.080534 0.291118 11.00000 -1.20000

H7B 2 0.774532 1.015951 0.391087 11.00000 -1.20000

AFIX 0

C8 1 1.171929 0.921696 0.509121 11.00000 0.07204 0.07387 =

0.05073 0.00743 0.02045 0.00261

C9 1 0.757185 0.756007 -0.198639 11.00000 0.07580 0.10903 =

0.06566 -0.00899 0.01935 0.01441

AFIX 37

H9A 2 0.636385 0.724828 -0.200942 11.00000 -1.50000

H9B 2 0.793415 0.721621 -0.270050 11.00000 -1.50000

H9C 2 0.745121 0.837483 -0.212546 11.00000 -1.50000

AFIX 0

C10 1 0.630541 0.972965 0.182305 11.00000 0.07014 0.08577 =

0.08992 -0.01547 0.02710 0.02439

AFIX 23

H10A 2 0.507517 0.979033 0.195091 11.00000 -1.20000

H10B 2 0.625972 1.007194 0.096062 11.00000 -1.20000

AFIX 0

C11 1 1.089195 0.731333 0.389686 11.00000 0.11972 0.06103 =

0.09626 0.01741 0.01489 0.01929

AFIX 37

H11A 2 0.983787 0.682464 0.339939 11.00000 -1.50000

H11B 2 1.164591 0.694655 0.474062 11.00000 -1.50000

H11C 2 1.168011 0.745351 0.335075 11.00000 -1.50000

AFIX 0

C12 1 1.360431 1.001241 0.727584 11.00000 0.11727 0.12598 =

0.05612 -0.01131 0.01064 0.02377

AFIX 43

H12 2 1.382957 1.007596 0.821202 11.00000 -1.20000

AFIX 0

C13 1 1.211379 0.935108 0.647181 11.00000 0.10845 0.10360 =

0.05267 0.00646 0.02865 0.00331

AFIX 43

H13 2 1.134022 0.897899 0.687842 11.00000 -1.20000

AFIX 0

C14 1 1.474975 1.057287 0.672415 11.00000 0.07098 0.11815 =

0.09098 -0.02433 -0.00465 0.00407

AFIX 43

H14 2 1.577990 1.100537 0.728110 11.00000 -1.20000

AFIX 0

C15 1 1.439253 1.050321 0.534909 11.00000 0.08497 0.17695 =

0.09465 -0.02511 0.02386 -0.05437

AFIX 43

H15 2 1.513340 1.091766 0.495105 11.00000 -1.20000

AFIX 0

C16 1 1.289466 0.979934 0.453993 11.00000 0.08439 0.16194 =

0.06485 -0.00446 0.02398 -0.03135

AFIX 43

H16 2 1.268906 0.972353 0.360771 11.00000 -1.20000

AFIX 0

H100 2 0.958662 0.842641 0.118178 11.00000 -1.50000

HKLF 4

REM fl_hd-1

REM R1 = 0.0734 for 2254 Fo > 4sig(Fo) and 0.1247 for all 3806 data

REM 187 parameters refined using 1 restraints

END

WGHT 0.1110 0.0000

REM Highest difference peak 0.522, deepest hole -0.602, 1-sigma level 0.198

Q1 4 0.9994 0.4585 -0.0982 11.00000 0.75 0.52

Q2 4 1.1010 0.4739 0.0064 11.00000 0.75 0.51

Q3 4 1.1281 0.5009 -0.0440 11.00000 0.75 0.51

Q4 4 0.8892 0.4511 -0.1592 11.00000 0.75 0.50

Q5 4 1.2180 0.5129 0.0366 11.00000 0.75 0.49

Q6 4 0.7354 1.0154 0.0052 11.00000 0.75 0.47

Q7 4 0.6715 0.9657 -0.0459 11.00000 0.75 0.46

Q8 4 0.8069 0.7809 0.5159 11.00000 0.75 0.39

Q9 4 0.7986 1.2343 0.3058 11.00000 0.75 0.39

Q10 4 0.6563 0.7761 0.4792 11.00000 0.75 0.39

Q11 4 0.9371 0.7332 0.5202 11.00000 0.75 0.39

Q12 4 0.7540 0.7458 0.4789 11.00000 0.75 0.38

Q13 4 0.9987 0.6993 0.5793 11.00000 0.75 0.38

Q14 4 1.1184 0.7087 0.5344 11.00000 0.75 0.37

Q15 4 0.5343 0.7904 0.4362 11.00000 0.75 0.36

Q16 4 1.5317 0.9406 0.9035 11.00000 0.75 0.36

Q17 4 0.7989 0.7130 0.4281 11.00000 0.75 0.35

Q18 4 0.9794 0.7317 0.4400 11.00000 0.75 0.32

Q19 4 0.4987 0.7941 0.3607 11.00000 0.75 0.31

Q20 4 1.3539 0.8075 0.3731 11.00000 0.75 0.31

Q21 4 1.4104 0.8533 0.3534 11.00000 0.75 0.31

Q22 4 1.2265 0.7890 0.3194 11.00000 0.75 0.27

Q23 4 1.4028 0.8961 0.8630 11.00000 0.75 0.24

Q24 4 0.4062 0.7343 -0.3390 11.00000 0.75 0.23

Q26 4 1.2579 0.6360 -0.1528 11.00000 0.75 0.17

Q27 4 1.0003 0.9778 -0.1523 11.00000 0.75 0.15

Q28 4 0.8645 0.5213 0.1122 11.00000 0.75 0.15

Q29 4 0.9497 0.5572 0.1956 11.00000 0.75 0.14

Q30 4 1.2284 1.1050 0.7335 11.00000 0.75 0.14

Q31 4 1.3620 0.9637 0.9619 11.00000 0.75 0.14

Q32 4 1.0308 0.7191 0.2674 11.00000 0.75 0.13

Q33 4 1.1492 0.6688 0.2985 11.00000 0.75 0.12

Q34 4 1.0358 0.5822 0.3025 11.00000 0.75 0.12

Q35 4 1.1343 0.9697 -0.1577 11.00000 0.75 0.11

Q36 4 0.5057 1.0185 0.1310 11.00000 0.75 0.09

**DFT and GIAO calculations**

***RRS*-6c** diastereoisomer ^1^H- and ^13^C-NMR theoretical prediction.

Conformational Search

%nprocshared=6

Will use up to 6 processors via shared memory.

%mem=500MW

%chk=OAAMeRRSs.chk

# opt=modredundant rb3lyp/3-21g

The following ModRedundant input section has been read:

D 43 16 17 19 S 5 30.000

D 2 3 11 12 S 5 30.000


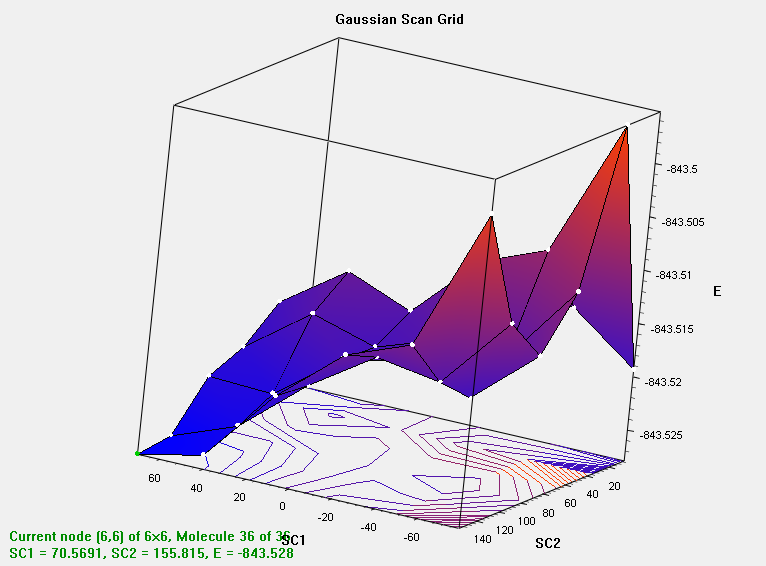


3-D Potential energy surface showing the variation of dihedral angles SC1 D(43,16,17,19) and SC2 D(2,3,11,12) and their corresponding energies(a. u.)

The 3 better conformational minima, reoptimized in vacuo at rb3lyp/6-311++g(d,p) level, converge towards the same structure. This lowest energy conformer was then considered for the fully unconstrained geometry optimization at SMD/DFT/B3LYP/6-311++G(d,p) level and vibrational analysis.


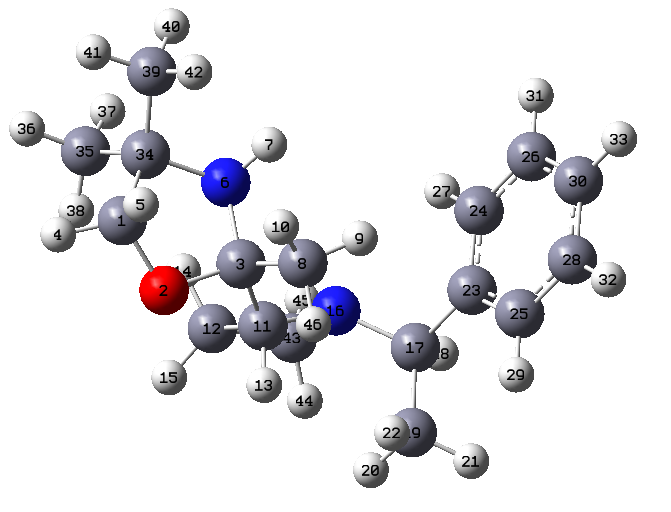


%nprocshared=6

Will use up to 6 processors via shared memory.

%chk=H:\G09\OAAMeRRSCDCl3.chk

%mem=99MW

# opt freq rb3lyp/6-311++g(d,p) scrf=(smd,solvent=chloroform)

-------------------------------------------------------------

Zero-point correction= 0.403884 (Hartree/Particle)

Thermal correction to Energy= 0.423548

Thermal correction to Enthalpy= 0.424492

Thermal correction to Gibbs Free Energy= 0.355756

Sum of electronic and zero-point Energies= -847.993193

Sum of electronic and thermal Energies= -847.973530

Sum of electronic and thermal Enthalpies= -847.972586

Sum of electronic and thermal Free Energies= -848.041322

________________________________________________________

%nprocshared=6

Will use up to 6 processors via shared memory.

%chk=H:\G09\OAAMeRRSCDCl3cs.chk

%mem=99MW

# MPW1PW91/gen scrf=(smd,solvent=chloroform)geom=check guess=read NMR

SCF GIAO Magnetic shielding tensor (ppm):

1 C Isotropic = 110.1563 Anisotropy = 58.2300

XX= 105.0616 YX= -27.5450 ZX= -7.5451

XY= -35.6239 YY= 111.2210 ZY= 17.9020

XZ= -3.1479 YZ= 19.7248 ZZ= 114.1862

Eigenvalues: 74.2681 107.2244 148.9762

2 O Isotropic = 229.6016 Anisotropy = 131.1031

XX= 246.8946 YX= -13.0752 ZX= 51.8588

XY= -28.6157 YY= 195.8132 ZY= 80.8520

XZ= 44.8238 YZ= 79.3600 ZZ= 246.0971

Eigenvalues: 120.2085 251.5927 317.0037

3 C Isotropic = 85.3938 Anisotropy = 54.8219

XX= 95.5413 YX= -8.8702 ZX= 28.1935

XY= -5.1258 YY= 59.8683 ZY= 14.8505

XZ= 18.0684 YZ= 12.1658 ZZ= 100.7718

Eigenvalues: 50.9901 83.2496 121.9417

4 H Isotropic = 28.0451 Anisotropy = 8.1261

XX= 32.0161 YX= -0.2248 ZX= 3.1797

XY= -1.5342 YY= 24.7534 ZY= 0.7611

XZ= 2.7045 YZ= 0.9028 ZZ= 27.3657

Eigenvalues: 24.0545 26.6183 33.4624

5 H Isotropic = 28.0389 Anisotropy = 7.6096

XX= 26.5823 YX= -4.1877 ZX= 0.8623

XY= -3.0566 YY= 28.4440 ZY= -2.8423

XZ= 0.8886 YZ= -2.6300 ZZ= 29.0904

Eigenvalues: 23.5616 27.4431 33.1120

6 N Isotropic = 167.7346 Anisotropy = 72.4679

XX= 208.4352 YX= -18.0030 ZX= -4.3660

XY= -24.0364 YY= 141.3857 ZY= 34.3519

XZ= 7.9288 YZ= 39.8003 ZZ= 153.3829

Eigenvalues: 106.8704 180.2869 216.0466

7 H Isotropic = 30.3778 Anisotropy = 9.8922

XX= 35.1831 YX= 3.4860 ZX= -0.7044

XY= 1.3712 YY= 32.0107 ZY= 4.7734

XZ= 0.5895 YZ= 4.7012 ZZ= 23.9397

Eigenvalues: 21.6648 32.4960 36.9726

8 C Isotropic = 163.0342 Anisotropy = 40.1118

XX= 160.1221 YX= 11.7739 ZX= -20.1696

XY= 11.2903 YY= 156.3858 ZY= -5.5161

XZ= -19.4253 YZ= -0.4919 ZZ= 172.5948

Eigenvalues: 141.3164 158.0108 189.7755

9 H Isotropic = 31.8881 Anisotropy = 14.0205

XX= 39.8254 YX= 3.0231 ZX= 3.8208

XY= -0.1107 YY= 28.0059 ZY= -1.0220

XZ= 4.5116 YZ= -0.4352 ZZ= 27.8330

Eigenvalues: 25.8951 28.5341 41.2351

10 H Isotropic = 30.5618 Anisotropy = 6.6640

XX= 31.3927 YX= -0.1692 ZX= -1.1650

XY= -2.5687 YY= 31.5828 ZY= -4.3635

XZ= -0.2657 YZ= -4.6311 ZZ= 28.7099

Eigenvalues: 25.1171 31.5638 35.0045

11 C Isotropic = 119.1442 Anisotropy = 36.0010

XX= 134.6586 YX= 0.3713 ZX= 19.5317

XY= -5.1039 YY= 125.1870 ZY= -15.7677

XZ= 12.5693 YZ= -7.7125 ZZ= 97.5869

Eigenvalues: 88.7051 125.5825 143.1448

12 C Isotropic = 168.1411 Anisotropy = 21.5137

XX= 162.2520 YX= -4.1569 ZX= 4.9864

XY= 3.6747 YY= 172.8492 ZY= -12.8595

XZ= 7.2174 YZ= -7.8795 ZZ= 169.3221

Eigenvalues: 156.5360 165.4037 182.4836

13 H Isotropic = 27.6450 Anisotropy = 3.9307

XX= 29.4411 YX= -0.5270 ZX= 1.3786

XY= -2.8696 YY= 26.5262 ZY= -0.0265

XZ= -0.1379 YZ= 0.9340 ZZ= 26.9676

Eigenvalues: 25.4441 27.2254 30.2654

14 H Isotropic = 29.0593 Anisotropy = 6.9847

XX= 31.1252 YX= -3.4253 ZX= -3.6021

XY= -2.1423 YY= 29.1287 ZY= -0.9435

XZ= -2.0264 YZ= -0.2228 ZZ= 26.9240

Eigenvalues: 24.6905 28.7716 33.7158

15 H Isotropic = 29.7790 Anisotropy = 10.1631

XX= 30.9284 YX= 4.8841 ZX= 1.0675

XY= 3.4933 YY= 33.0080 ZY= 1.5348

XZ= 0.5948 YZ= 1.5726 ZZ= 25.4006

Eigenvalues: 25.0955 27.6871 36.5544

16 N Isotropic = 188.1464 Anisotropy = 48.7374

XX= 218.2347 YX= -1.5606 ZX= -2.2195

XY= -18.7762 YY= 166.2106 ZY= -24.8770

XZ= 2.0928 YZ= -18.7208 ZZ= 179.9941

Eigenvalues: 149.2449 194.5564 220.6380

17 C Isotropic = 126.3122 Anisotropy = 37.9708

XX= 149.8093 YX= 6.6069 ZX= -5.1099

XY= 1.7036 YY= 123.3990 ZY= 7.4107

XZ= -10.2022 YZ= -4.3451 ZZ= 105.7284

Eigenvalues: 104.1799 123.1308 151.6261

18 H Isotropic = 27.8115 Anisotropy = 4.9973

XX= 28.2990 YX= -3.0600 ZX= 3.0993

XY= 0.6835 YY= 26.7792 ZY= -1.3416

XZ= 1.2466 YZ= -1.0170 ZZ= 28.3564

Eigenvalues: 26.1274 26.1642 31.1431

19 C Isotropic = 179.1480 Anisotropy = 4.0015

XX= 178.3902 YX= -6.3417 ZX= 1.9144

XY= 4.7394 YY= 179.4517 ZY= 3.5327

XZ= 2.4557 YZ= 0.1607 ZZ= 179.6023

Eigenvalues: 175.8328 179.7956 181.8157

20 H Isotropic = 29.8512 Anisotropy = 8.2301

XX= 27.9457 YX= 0.8399 ZX= -2.4841

XY= 2.2576 YY= 34.8529 ZY= 1.5463

XZ= -2.0012 YZ= 1.6466 ZZ= 26.7552

Eigenvalues: 24.5554 29.6604 35.3380

21 H Isotropic = 30.0696 Anisotropy = 10.9511

XX= 34.8397 YX= -3.6204 ZX= -4.1321

XY= -2.0256 YY= 29.1486 ZY= 2.2323

XZ= -3.0050 YZ= 0.9472 ZZ= 26.2205

Eigenvalues: 24.8493 27.9892 37.3703

22 H Isotropic = 30.0639 Anisotropy = 6.3994

XX= 28.8907 YX= -0.8434 ZX= 0.6053

XY= -1.2593 YY= 27.1970 ZY= -1.2588

XZ= 0.3637 YZ= -0.8060 ZZ= 34.1040

Eigenvalues: 26.6214 29.2401 34.3302

23 C Isotropic = 32.8288 Anisotropy = 202.7932

XX= 98.3070 YX= -81.6686 ZX= 53.5054

XY= -84.3923 YY= -18.8322 ZY= -23.0882

XZ= 58.2021 YZ= -35.4172 ZZ= 19.0117

Eigenvalues: -61.8731 -7.6648 168.0243

24 C Isotropic = 50.9963 Anisotropy = 191.8615

XX= 95.9156 YX= -60.9747 ZX= 89.2625

XY= -62.7043 YY= 46.9250 ZY= -11.5555

XZ= 95.2891 YZ= -1.6888 ZZ= 10.1484

Eigenvalues: -57.2739 31.3589 178.9040

25 C Isotropic = 56.6102 Anisotropy = 184.2540

XX= 127.7226 YX= -40.0745 ZX= 58.7619

XY= -37.2603 YY= 34.9236 ZY= -68.8858

XZ= 58.4935 YZ= -64.6633 ZZ= 7.1844

Eigenvalues: -50.1929 40.5773 179.4462

26 C Isotropic = 53.8535 Anisotropy = 186.5004

XX= 128.6555 YX= -38.2322 ZX= 54.7900

XY= -37.2037 YY= 31.8340 ZY= -69.3799

XZ= 60.5501 YZ= -70.2622 ZZ= 1.0710

Eigenvalues: -57.9361 41.3094 178.1871

27 H Isotropic = 23.1929 Anisotropy = 9.9835

XX= 22.0785 YX= 3.4896 ZX= 2.2029

XY= 2.6361 YY= 25.5599 ZY= 4.5296

XZ= 1.5421 YZ= 3.5813 ZZ= 21.9404

Eigenvalues: 19.3023 20.4279 29.8486

28 C Isotropic = 54.1468 Anisotropy = 186.1939

XX= 95.1003 YX= -62.2114 ZX= 93.5712

XY= -62.6512 YY= 54.6530 ZY= -1.8881

XZ= 89.4563 YZ= -1.8992 ZZ= 12.6872

Eigenvalues: -57.0922 41.2566 178.2761

29 H Isotropic = 23.8164 Anisotropy = 10.5846

XX= 24.7805 YX= 4.3989 ZX= -3.3114

XY= 4.7404 YY= 25.7669 ZY= -2.0446

XZ= -2.5753 YZ= -1.1162 ZZ= 20.9020

Eigenvalues: 19.1431 21.4334 30.8729

30 C Isotropic = 56.1463 Anisotropy = 185.6174

XX= 110.2665 YX= -84.3343 ZX= 47.4332

XY= -84.2650 YY= -9.0512 ZY= -25.4843

XZ= 46.6294 YZ= -28.2938 ZZ= 67.2237

Eigenvalues: -52.7155 41.2632 179.8912

31 H Isotropic = 23.5969 Anisotropy = 5.5208

XX= 23.3358 YX= 3.2595 ZX= -1.3231

XY= 3.0907 YY= 24.5163 ZY= 0.1912

XZ= -1.1451 YZ= 0.0032 ZZ= 22.9387

Eigenvalues: 20.2981 23.2152 27.2775

32 H Isotropic = 23.8232 Anisotropy = 5.2336

XX= 22.5166 YX= 2.1723 ZX= -0.9071

XY= 2.2458 YY= 25.7528 ZY= 1.8542

XZ= -1.0408 YZ= 1.9558 ZZ= 23.2002

Eigenvalues: 20.2795 23.8778 27.3123

33 H Isotropic = 23.9116 Anisotropy = 4.0817

XX= 23.0093 YX= 1.4668 ZX= -2.0955

XY= 1.2142 YY= 23.4033 ZY= 1.3590

XZ= -2.1440 YZ= 1.0860 ZZ= 25.3224

Eigenvalues: 20.6112 24.4909 26.6328

34 C Isotropic = 125.5702 Anisotropy = 47.7724

XX= 150.3506 YX= -18.0016 ZX= 11.1911

XY= -14.2950 YY= 103.4573 ZY= -1.3568

XZ= 8.1576 YZ= 7.4555 ZZ= 122.9027

Eigenvalues: 97.0759 122.2162 157.4184

35 C Isotropic = 157.6656 Anisotropy = 43.2325

XX= 168.0735 YX= 14.1580 ZX= -12.0079

XY= 17.1885 YY= 144.6293 ZY= -12.6342

XZ= -10.5503 YZ= -15.0665 ZZ= 160.2940

Eigenvalues: 134.0747 152.4349 186.4872

36 H Isotropic = 30.6784 Anisotropy = 11.2782

XX= 37.9539 YX= -0.6401 ZX= -1.6389

XY= 0.8302 YY= 27.1269 ZY= -0.3195

XZ= -1.6505 YZ= -0.7480 ZZ= 26.9545

Eigenvalues: 26.3653 27.4727 38.1972

37 H Isotropic = 30.5071 Anisotropy = 7.9587

XX= 28.7564 YX= 0.2092 ZX= -2.6298

XY= -0.1581 YY= 27.4143 ZY= -0.9530

XZ= -0.8879 YZ= 0.0690 ZZ= 35.3505

Eigenvalues: 27.3845 28.3239 35.8128

38 H Isotropic = 29.9437 Anisotropy = 5.8913

XX= 30.4992 YX= 1.5558 ZX= -2.8337

XY= 0.3171 YY= 32.9858 ZY= -0.6711

XZ= -1.8934 YZ= -1.8477 ZZ= 26.3462

Eigenvalues: 25.1992 30.7607 33.8712

39 C Isotropic = 160.4451 Anisotropy = 44.7606

XX= 150.8126 YX= -4.7761 ZX= -9.5707

XY= -1.9924 YY= 189.9500 ZY= -1.9215

XZ= -10.3569 YZ= 3.1401 ZZ= 140.5728

Eigenvalues: 134.4623 156.5875 190.2855

40 H Isotropic = 30.0598 Anisotropy = 8.6723

XX= 27.1791 YX= -1.0524 ZX= -0.8156

XY= -0.9047 YY= 31.3245 ZY= 5.0300

XZ= 0.1760 YZ= 3.4540 ZZ= 31.6758

Eigenvalues: 26.6795 27.6585 35.8413

41 H Isotropic = 30.3624 Anisotropy = 11.2320

XX= 33.8856 YX= -4.6932 ZX= 0.5052

XY= -5.4188 YY= 31.3600 ZY= -0.1855

XZ= 0.3092 YZ= -0.2086 ZZ= 25.8415

Eigenvalues: 25.8195 27.4172 37.8504

42 H Isotropic = 30.7255 Anisotropy = 8.0031

XX= 29.3606 YX= 0.3371 ZX= -2.4077

XY= 1.7503 YY= 34.8935 ZY= -2.9060

XZ= -1.9151 YZ= -1.8895 ZZ= 27.9223

Eigenvalues: 26.1325 29.9830 36.0609

43 C Isotropic = 136.2567 Anisotropy = 56.1919

XX= 141.1200 YX= 28.0622 ZX= -12.1484

XY= 23.1171 YY= 138.4200 ZY= -12.7911

XZ= -10.6528 YZ= -18.9489 ZZ= 129.2302

Eigenvalues: 112.7298 122.3224 173.7180

44 H Isotropic = 28.1281 Anisotropy = 6.8632

XX= 26.3771 YX= 0.1465 ZX= -0.8061

XY= -1.2392 YY= 31.9688 ZY= -1.1978

XZ= -0.9362 YZ= -3.1955 ZZ= 26.0383

Eigenvalues: 24.7119 26.9688 32.7036

45 H Isotropic = 28.0780 Anisotropy = 9.8360

XX= 24.6787 YX= -0.0953 ZX= 0.3200

XY= -0.1715 YY= 26.4913 ZY= -4.6283

XZ= 0.2790 YZ= -2.4967 ZZ= 33.0641

Eigenvalues: 24.6679 24.9309 34.6353

46 H Isotropic = 31.1586 Anisotropy = 8.2307

XX= 31.3378 YX= -1.6032 ZX= -3.1532

XY= -2.2079 YY= 27.8155 ZY= -0.9041

XZ= -3.2486 YZ= 2.1840 ZZ= 34.3226

Eigenvalues: 26.8937 29.9364 36.6458

%nprocshared=6

Will use up to 6 processors via shared memory.

%chk=H:\G09\OAAMeRRSCDCl3J.chk

%mem=99MW

# rb3lyp/6-311++g(d,p) scrf=(smd,solvent=chloroform) geom=check guess=

read NMR=spinspin

Total nuclear spin-spin coupling J (Hz):

1 2 3 4 5

1 0.000000D+00

2 0.211590D+02 0.000000D+00

3 0.162067D+00 0.229025D+02 0.000000D+00

4 0.140097D+03 -0.123939D+02 0.802156D+01 0.000000D+00

5 0.135856D+03 -0.352976D+01 -0.263677D+00 -0.762360D+01 0.000000D+00

6 0.871428D-02 0.525608D+00 0.138774D+01 0.694399D+00 -0.364381D-01

7 0.644183D+00 -0.376427D+00 -0.459958D+00 -0.758982D+00 -0.220904D+00

8 -0.126428D+00 -0.150419D+01 0.382265D+02 -0.662762D+00 -0.103391D+00

9 -0.226501D+00 -0.541553D+01 -0.105876D+01 -0.519169D-01 -0.216946D+00

10 0.124689D+00 -0.130386D+01 -0.527031D+01 -0.252578D+00 0.260331D+00

11 0.200606D+01 -0.129860D+02 0.534681D+02 -0.471456D+00 0.168639D+00

12 -0.786739D-01 -0.842042D+00 -0.182702D+01 -0.323225D-01 0.747961D-01

13 -0.126453D+00 -0.128373D+01 0.377070D+00 -0.112967D+00 -0.127389D+00

14 0.284733D-01 0.965516D-01 0.444709D+01 -0.178023D-01 -0.151740D+00

15 -0.824910D-03 -0.513976D-01 0.127418D+01 -0.271147D-01 -0.138611D+00

16 0.290964D+00 -0.110999D+01 0.349842D+01 0.181788D+00 0.627907D-02

17 -0.355619D-02 0.281150D-01 0.506172D+00 -0.187968D-01 -0.165617D-01

18 0.263856D-01 -0.513421D+00 -0.870950D-01 -0.121217D+00 -0.119023D+00

19 -0.105057D-01 -0.128351D-01 -0.151546D+00 -0.190754D-01 -0.141196D-01

20 -0.170761D-01 -0.297382D-02 0.164342D-01 -0.693183D-01 -0.785050D-01

21 -0.220340D-01 0.273792D-02 -0.103479D+00 -0.915455D-01 -0.850590D-01

22 -0.205936D-02 -0.750978D-01 -0.419420D-01 -0.560346D-01 -0.159183D-01

23 -0.954526D-02 -0.722248D-02 0.266810D+00 -0.158276D-01 0.246398D-02

24 -0.166278D-02 0.530291D-02 0.703845D-01 -0.172486D-01 0.178550D-02

25 -0.208356D-02 0.767406D-02 -0.257307D-01 -0.149795D-01 0.498803D-02

26 -0.405700D-02 0.201335D-02 0.723508D-02 -0.152386D-01 0.214675D-02

27 -0.189408D-01 0.156304D-01 0.383963D-01 -0.826288D-01 -0.531961D-01

28 -0.499886D-02 0.204673D-02 0.254978D-01 -0.164626D-01 0.163807D-02

29 -0.187897D-01 0.364530D-02 0.291918D-02 -0.742685D-01 -0.265183D-01

30 -0.350297D-02 0.394481D-02 0.932172D-02 -0.182496D-01 -0.173490D-03

31 -0.183501D-01 0.146414D-01 0.119253D-02 -0.783526D-01 -0.370055D-01

32 -0.186943D-01 0.108735D-01 -0.113038D-01 -0.756037D-01 -0.268416D-01

33 -0.202669D-01 0.129404D-01 -0.348306D-02 -0.785396D-01 -0.327621D-01

34 0.334702D+02 0.229932D+00 0.787546D-01 -0.162404D+00 -0.296309D+00

35 0.283981D+01 -0.110810D-01 0.397231D+00 0.373606D+01 0.506717D+01

36 0.111258D+01 -0.250730D-01 0.101876D+00 0.358328D+00 -0.197740D+00

37 0.837397D+01 0.836178D-01 -0.146262D+00 0.779375D+00 0.206391D+01

38 0.264531D+01 -0.609545D-01 -0.840815D-01 0.781420D-01 -0.196497D+00

39 0.258920D+01 -0.305392D+00 0.167226D+01 0.465546D+00 0.461346D+01

40 0.105411D+02 0.138317D-01 -0.283599D+00 -0.478857D+00 0.124792D+00

41 0.173759D+01 0.204821D+00 0.149397D+00 -0.140002D+00 -0.582826D-01

42 0.288173D+01 0.797816D-01 -0.130748D+00 -0.113005D+00 0.211192D+00

43 0.298467D-01 0.149715D+00 0.566571D+01 0.922465D-02 -0.235890D-01

44 -0.231717D-01 0.417816D-01 0.263673D+00 -0.939427D-01 -0.139750D+00

45 -0.251646D-01 -0.441965D-01 0.175403D+00 -0.120855D+00 -0.145556D+00

46 -0.147215D+00 -0.807249D+00 -0.317498D+01 -0.471630D-01 0.769689D-01

6 7 8 9 10

6 0.000000D+00

7 0.461860D+02 0.000000D+00

8 -0.548924D+00 0.407410D+01 0.000000D+00

9 0.388986D+00 0.292113D+00 0.119011D+03 0.000000D+00

10 0.596094D-01 -0.310235D-01 0.119650D+03 -0.122663D+02 0.000000D+00

11 0.363764D+01 0.180201D+01 0.578720D+01 0.262550D+00 0.605263D+01

12 0.113931D+01 0.210976D+00 0.180172D+01 -0.111143D+00 0.168943D+01

13 0.227789D+01 -0.225668D+00 0.154352D+01 0.354517D-01 -0.183649D+00

14 -0.726177D-01 -0.849192D-01 -0.367143D+00 -0.562372D-01 -0.166664D+00

15 0.512410D-01 -0.234926D+00 -0.207977D+00 -0.128774D+00 -0.195012D+00

16 0.969480D-01 0.187133D-01 0.351106D-01 0.125991D+00 -0.956182D-01

17 -0.233702D-01 0.110272D-01 -0.341302D-01 0.192433D+00 0.736179D-01

18 -0.113224D-01 -0.669221D-01 -0.537908D-01 -0.107458D+00 -0.217796D+00

19 0.845387D-01 -0.307144D-01 0.223839D-01 0.306813D-03 0.138098D-01

20 0.149439D-01 -0.107784D+00 -0.155899D-01 -0.762671D-01 -0.125821D+00

21 0.690110D-01 -0.104531D+00 0.249054D-01 -0.481416D-01 -0.106676D+00

22 0.183139D-01 -0.423222D-01 0.180376D+00 0.201446D+00 0.350272D-01

23 0.139587D-01 0.838133D-01 0.424196D+00 0.292420D+00 0.191458D+00

24 0.275335D-01 0.691584D-01 0.211487D+00 0.350273D+00 0.758757D-01

25 0.369573D-04 0.264521D-01 0.207127D+00 0.392491D+00 0.919546D-01

26 0.645514D-02 0.811311D-01 -0.627076D-01 0.934519D-01 -0.882861D-02

27 0.414223D-01 0.260357D+00 -0.583387D-01 0.172031D+00 -0.762551D-01

28 0.355834D-02 0.143374D-01 -0.677633D-01 0.818382D-01 -0.690767D-02

29 -0.821474D-03 -0.335989D-01 -0.623324D-01 0.143843D+00 -0.473801D-01

30 -0.239861D-02 0.266008D-01 0.622093D-01 0.950954D-01 0.500686D-01

31 0.335646D-02 0.144447D+00 0.204659D-01 0.164538D-01 -0.329276D-01

32 -0.583870D-02 -0.261620D-01 0.242344D-01 -0.229323D-03 -0.181879D-01

33 0.828636D-04 0.196473D-01 -0.391889D-01 -0.271211D-01 -0.619088D-01

34 0.218955D+01 -0.674730D+00 -0.300782D+00 0.149046D+00 -0.104865D+00

35 0.495284D+01 0.359332D+01 0.130611D+01 -0.145168D-01 0.845953D-01

36 0.498404D+01 0.732929D+00 0.356838D+00 -0.119048D+00 -0.124389D+00

37 0.885692D+00 -0.365152D+00 0.207924D-01 -0.728871D-01 -0.128657D+00

38 0.567788D+00 -0.363572D+00 -0.204734D-01 -0.121996D+00 -0.125056D+00

39 -0.275753D+00 0.510442D+01 -0.161739D+00 -0.311807D-01 0.398701D-01

40 0.524972D+00 -0.760112D-02 -0.442182D-01 -0.299649D-01 -0.639020D-01

41 0.239456D+01 -0.287549D+00 -0.130473D+00 -0.108072D+00 -0.293830D-01

42 0.473377D+00 0.686396D-01 0.574025D-01 0.746245D-01 0.305762D+00

43 -0.577788D-01 -0.195836D-01 -0.377995D+00 0.134893D-01 -0.161062D+00

44 0.147002D+00 -0.189593D+00 -0.230895D-01 -0.112392D+00 -0.186092D+00

45 0.616178D-01 -0.521862D-01 0.184498D+00 -0.902229D-01 -0.104840D+00

46 0.130664D+01 0.371937D-01 0.119696D+03 -0.118333D+02 -0.113656D+02

11 12 13 14 15

11 0.000000D+00

12 0.313098D+02 0.000000D+00

13 0.129940D+03 -0.246015D+01 0.000000D+00

14 -0.444533D+01 0.132054D+03 0.663581D+01 0.000000D+00

15 -0.140547D+01 0.133059D+03 0.741768D+01 -0.864610D+01 0.000000D+00

16 0.109116D+01 0.416508D+00 -0.124223D+00 0.629047D-01 0.371426D+01

17 -0.157500D+01 0.661173D+01 0.397131D+01 -0.222418D+00 0.162918D+01

18 0.698488D+01 -0.468751D+00 -0.719217D+00 -0.105496D+00 -0.333074D+00

19 0.388769D+01 -0.186656D+00 0.115972D+00 0.527935D+00 -0.759746D-01

20 0.376642D+00 -0.334491D-02 0.219577D+00 -0.105755D+00 0.180477D+00

21 0.849968D+00 -0.830641D-01 -0.238181D+00 -0.119235D-01 0.181068D+00

22 0.233953D+00 0.155656D-02 0.243922D+00 -0.125829D+00 0.194415D-02

23 -0.247757D+00 0.115565D+00 0.159859D+00 0.122499D+00 -0.419307D-01

24 -0.488390D-01 -0.892304D-02 0.129542D+00 -0.106095D-01 -0.920799D-02

25 -0.202023D-01 0.121075D+00 -0.295345D-01 -0.318931D-02 0.449048D-01

26 -0.462841D-02 -0.621217D-02 -0.165422D-01 -0.746271D-02 -0.425573D-01

27 0.292162D-01 0.825283D-02 -0.110913D+00 0.714221D-01 -0.917125D-01

28 -0.292531D-01 0.217366D-01 -0.810832D-02 -0.223588D-01 -0.364246D-01

29 -0.159096D-01 -0.215662D-01 0.809776D-01 -0.997806D-01 -0.900237D-01

30 0.294979D-02 -0.134097D-01 -0.275462D-01 -0.277815D-01 -0.179695D-01

31 -0.136654D-01 -0.241626D-01 -0.115570D+00 -0.294723D-01 -0.924672D-01

32 -0.181689D-01 -0.112411D-01 -0.643258D-01 -0.974740D-01 -0.794367D-01

33 -0.285153D-01 -0.245489D-01 -0.914604D-01 -0.823471D-01 -0.115302D+00

34 0.147940D+01 -0.142303D+00 0.403993D+00 0.136581D+00 -0.143483D-01

35 0.329939D-01 0.100895D+00 -0.201047D-01 0.563275D+00 -0.176996D-01

36 -0.116303D+00 0.209331D+00 -0.161787D+00 -0.386312D-01 -0.772684D-01

37 -0.190442D-01 -0.312269D-01 -0.150801D+00 0.266538D+00 -0.124492D-01

38 -0.294802D-02 0.785975D+00 -0.601960D-01 0.279098D+00 0.196017D+00

39 0.561136D-01 0.157994D+00 0.298823D+00 -0.267551D-01 -0.303561D-01

40 0.788685D-01 -0.201829D-01 -0.145388D+00 0.131845D-01 -0.101003D+00

41 0.515584D+00 -0.319484D-01 0.421318D-01 -0.489578D-01 -0.100661D+00

42 0.784960D-01 -0.259543D-01 -0.824954D-01 -0.646265D-01 -0.119725D+00

43 -0.574862D+01 0.283679D+02 0.465373D+00 -0.410101D+01 -0.157611D+01

44 0.390641D+00 -0.311784D+01 -0.487643D+00 0.743510D+01 0.812446D+01

45 0.839943D+01 -0.223561D+01 -0.976143D+00 0.815643D+01 0.201845D+01

46 0.204650D+01 0.172872D+00 0.583991D+00 -0.169007D+00 -0.110302D+00

16 17 18 19 20

16 0.000000D+00

17 0.198366D+00 0.000000D+00

18 -0.315690D-01 0.125521D+03 0.000000D+00

19 -0.768878D+00 0.357063D+02 -0.369511D+01 0.000000D+00

20 0.348928D+00 -0.340436D+01 0.315432D+01 0.119140D+03 0.000000D+00

21 0.176266D+01 -0.122647D+01 0.413948D+01 0.118945D+03 -0.128316D+02

22 0.521460D+00 -0.451697D+01 0.115661D+02 0.120161D+03 -0.111242D+02

23 0.261540D+01 0.474448D+02 -0.657095D+01 0.150664D+01 0.816895D+01

24 0.145800D+01 0.186572D+01 0.254645D+01 0.213898D+01 0.627932D+00

25 0.794675D+00 0.305124D+01 0.222385D+01 0.352158D+01 0.146038D+01

26 -0.186945D+00 0.311223D+01 -0.127757D+01 0.430310D+00 0.392838D+00

27 -0.868959D-01 0.374461D+01 -0.768241D+00 -0.137214D+00 0.218410D-01

28 -0.593606D-01 0.336433D+01 -0.113813D+01 0.186683D+00 0.105721D-01

29 -0.253339D+00 0.320215D+01 -0.125147D+01 0.441091D+00 -0.146870D+00

30 0.313109D+00 -0.682961D+00 0.105573D+01 0.152763D+00 0.802965D-01

31 0.765537D-01 0.365171D+00 0.270644D+00 0.191132D+00 -0.304782D-01

32 0.143799D+00 0.614506D+00 0.295684D+00 -0.481613D-01 -0.693588D-01

33 -0.187741D+00 0.416628D+00 -0.104518D+01 -0.583564D-01 -0.116486D+00

34 -0.642130D-01 -0.215556D-02 -0.346235D-01 0.119517D-01 -0.128209D-01

35 -0.725770D-02 -0.141529D-02 -0.206443D-01 -0.265499D-02 -0.153145D-01

36 -0.860951D-02 -0.200482D-01 -0.946687D-01 -0.228139D-01 -0.721126D-01

37 0.598163D-02 -0.788926D-02 -0.444594D-01 -0.188269D-01 -0.560650D-01

38 0.100155D-03 -0.618393D-02 -0.521325D-01 -0.129217D-01 -0.124989D-01

39 -0.587563D-02 -0.773510D-02 -0.212715D-01 -0.445345D-02 -0.169846D-01

40 -0.756759D-03 -0.124284D-01 -0.590611D-01 -0.176045D-01 -0.712498D-01

41 -0.660978D-02 -0.235619D-01 -0.978888D-01 -0.122429D-01 -0.765178D-01

42 -0.409291D-02 -0.110917D-01 -0.769366D-01 -0.127426D-01 -0.666026D-01

43 0.196082D+01 -0.821465D+00 0.651218D+01 0.222616D+00 0.218623D+00

44 -0.491583D-01 0.577336D+01 0.847759D-01 0.197937D-01 0.131363D+00

45 0.386437D+01 0.186409D+00 -0.974267D-02 -0.290320D+00 -0.172858D+00

46 0.618532D-01 0.703024D-02 -0.139602D+00 0.177476D+00 0.191236D-01

21 22 23 24 25

21 0.000000D+00

22 -0.114904D+02 0.000000D+00

23 0.298453D+00 0.230466D+01 0.000000D+00

24 -0.126753D+00 -0.228036D+00 0.611663D+02 0.000000D+00

25 0.943212D+00 0.283473D+00 0.613126D+02 -0.210564D-01 0.000000D+00

26 -0.495923D-01 -0.412011D-01 -0.131124D+01 0.602702D+02 0.867691D+01

27 -0.945121D-01 -0.975167D-01 -0.458121D+00 0.150374D+03 0.573706D+01

28 0.151358D+00 0.409680D-01 -0.156734D+01 0.889812D+01 0.592545D+02

29 0.333080D+00 0.240187D+00 0.350455D+00 0.591923D+01 0.148240D+03

30 -0.412159D-01 -0.133108D-01 0.832928D+01 -0.199734D+01 -0.194321D+01

31 -0.166103D+00 -0.179430D+00 0.647960D+01 0.183535D+01 -0.113445D+01

32 -0.159450D-01 0.267481D-01 0.636064D+01 -0.114791D+01 0.197488D+01

33 -0.104894D+00 -0.874760D-01 -0.129579D+01 0.662977D+01 0.681704D+01

34 -0.135381D-01 -0.112732D-01 0.173877D-02 0.124196D-01 -0.101629D-02

35 -0.224564D-01 -0.172313D-01 0.195344D-02 -0.261138D-02 -0.293773D-02

36 -0.905551D-01 -0.730674D-01 -0.153867D-01 -0.109311D-01 -0.156033D-01

37 -0.779047D-01 -0.665330D-01 -0.400505D-02 0.793311D-02 -0.110174D-01

38 -0.694258D-01 -0.328099D-01 -0.803842D-02 -0.286419D-02 -0.109139D-01

39 -0.163997D-01 -0.872726D-02 0.331266D-03 0.235140D-02 -0.126628D-02

40 -0.742109D-01 -0.575801D-01 0.657399D-02 0.162501D-01 -0.625079D-02

41 -0.790236D-01 -0.613545D-01 -0.872687D-02 -0.380517D-02 -0.114678D-01

42 -0.628595D-01 -0.175696D-01 0.171544D-01 0.214016D-01 0.845759D-02

43 -0.103838D+00 -0.159360D+00 0.374586D+01 -0.185952D+00 0.468282D+00

44 0.152037D-01 -0.749748D-01 -0.361591D+00 -0.686887D-02 -0.337542D-01

45 -0.152148D+00 -0.992536D-01 -0.210441D+00 -0.250193D-01 -0.597937D-01

46 -0.453682D-02 0.376828D+00 0.592832D-01 0.212392D-01 0.748717D-01

26 27 28 29 30

26 0.000000D+00

27 0.148519D+01 0.000000D+00

28 -0.138186D+01 -0.855354D+00 0.000000D+00

29 -0.888964D+00 0.135652D+01 0.101366D+01 0.000000D+00

30 0.584700D+02 0.705055D+01 0.596915D+02 0.659988D+01 0.000000D+00

31 0.149421D+03 0.738786D+01 0.687628D+01 0.365876D+00 0.166701D+01

32 0.709730D+01 0.398223D+00 0.149471D+03 0.722599D+01 0.150561D+01

33 0.176166D+01 0.701152D+00 0.170610D+01 0.621372D+00 0.150274D+03

34 0.384046D-02 0.262188D-01 -0.119947D-02 -0.148091D-01 -0.197907D-02

35 -0.499015D-03 0.108435D-02 -0.397377D-02 -0.174449D-01 -0.306412D-02

36 -0.967470D-02 -0.422738D-01 -0.177220D-01 -0.747577D-01 -0.148200D-01

37 0.348228D-02 0.705467D-01 -0.163367D-01 -0.664494D-01 -0.881718D-02

38 -0.674887D-02 0.116485D-01 -0.180715D-01 -0.568866D-01 -0.163681D-01

39 0.239084D-02 -0.353329D-03 -0.149000D-02 -0.150017D-01 0.305233D-03

40 0.167243D-01 0.656771D-01 -0.708830D-02 -0.617594D-01 0.406478D-02

41 -0.396479D-02 -0.441867D-01 -0.117864D-01 -0.699517D-01 -0.817988D-02

42 0.239979D-01 0.265092D-01 0.800579D-02 -0.275669D-01 0.153496D-01

43 0.198474D-01 0.944485D-01 0.217896D+00 0.938817D-01 -0.417339D-01

44 -0.330138D-02 0.625347D-02 -0.459940D-01 -0.459497D-01 -0.480015D-01

45 0.150012D-01 0.230378D+00 -0.452099D-01 -0.106630D+00 -0.482306D-01

46 0.101658D-01 -0.684968D-01 0.539801D-01 0.223250D+00 -0.135599D-01

31 32 33 34 35

31 0.000000D+00

32 0.829478D+00 0.000000D+00

33 0.663757D+01 0.681061D+01 0.000000D+00

34 -0.119103D-02 -0.163775D-01 -0.114950D-01 0.000000D+00

35 -0.577333D-02 -0.165268D-01 -0.139365D-01 0.408576D+02 0.000000D+00

36 -0.466038D-01 -0.689004D-01 -0.614164D-01 -0.901854D+00 0.115112D+03

37 0.132195D-01 -0.620495D-01 -0.414265D-01 -0.440448D+01 0.121205D+03

38 -0.398422D-01 -0.675959D-01 -0.653049D-01 -0.419794D+01 0.120825D+03

39 0.157571D-03 -0.117353D-01 -0.665603D-02 0.365485D+02 0.377840D+01

40 0.508476D-01 -0.486899D-01 -0.138417D-01 -0.410123D+01 0.245813D+01

41 -0.375586D-01 -0.603114D-01 -0.492457D-01 -0.179570D+01 0.123534D+01

42 0.369100D-01 -0.145826D-01 0.835438D-02 -0.356564D+01 0.771605D+01

43 -0.219696D-01 0.362842D-01 -0.756757D-02 0.559138D-01 0.125858D+00

44 -0.105531D+00 -0.108429D+00 -0.979323D-01 -0.373612D-01 0.234554D-01

45 -0.239289D-01 -0.119023D+00 -0.786108D-01 0.144516D-02 -0.264115D-01

46 -0.118771D+00 0.253267D-03 -0.775502D-01 0.867193D-01 0.428842D+00

36 37 38 39 40

36 0.000000D+00

37 -0.112688D+02 0.000000D+00

38 -0.114478D+02 -0.113592D+02 0.000000D+00

39 0.203473D+01 0.189473D+01 0.913132D+01 0.000000D+00

40 -0.240979D+00 0.176801D+00 -0.218981D+00 0.119696D+03 0.000000D+00

41 0.606727D+00 -0.253539D+00 -0.342444D-01 0.118342D+03 -0.127432D+02

42 0.229966D+00 -0.300063D+00 0.335514D+01 0.119280D+03 -0.108200D+02

43 -0.147870D-01 -0.143247D-01 0.382988D-01 -0.142489D-01 -0.199804D-01

44 -0.812924D-01 -0.782525D-01 -0.149516D-01 -0.176392D-01 -0.927884D-01

45 -0.683897D-01 0.567917D-01 0.455039D-01 -0.152945D-01 -0.164173D-01

46 0.193429D-01 -0.126100D+00 -0.129530D+00 -0.424474D-01 -0.157122D+00

41 42 43 44 45

41 0.000000D+00

42 -0.117491D+02 0.000000D+00

43 -0.140642D-01 -0.186712D-01 0.000000D+00

44 -0.108765D+00 -0.106601D+00 0.128067D+03 0.000000D+00

45 -0.785261D-01 -0.722270D-01 0.140503D+03 -0.496905D+01 0.000000D+00

46 -0.861187D-01 -0.289123D-01 -0.785964D-02 -0.519373D-01 -0.142200D+00

46

46 0.000000D+00

Table 1. Experimental and calculated ^1^H and^13^C NMR data for the ***RRS*-6c** diastereoisomer with his HSQC correlations

| **Label H** | **δ_H_ exp** | **δ_H_ scaled^1^** | **Label C (HSQCcorr)** | **δ_C_ exp** | **δ_C_ scaled^1^** |
| --- | --- | --- | --- | --- | --- |
| H_13_ | 3,35 | 3,67 | C_3_ | 98,98 | 99,04 |
| H_18_ | 3,64 | 3,50 | C_1_ (H_4_, H_5_) | 77,47 | 75,88 |
| H_4_ | 3,72 | 3,28 | C_11_ | 67,63 | 67,47 |
| H_5_ | 3,54 | 3,29 | C_17_ (H_18_) | 64,12 | 60,76 |
| H_44_ | 2,99 | 3,26 | C_34_ | 59,63 | 61,45 |
| H_45_ | 2,69 | 3,21 | C_43_ (H_44_, H_45_) | 46,04 | 51,45 |
| H_14_ | 1,87 | 2,32 | C_39_ (H_37_) | 29,66 | 28,83 |
| H_15_ | 1,83 | 1,62 | C_35_ (H_41_) | 28,4 | 31,42 |
| H_21_ | 1,31 | 1,42 | C_8_ (H_10_) | 22,92 | 26,40 |
| H_10_ | 1,39 | 1,25 | C_19_ (H_21_) | 20,83 | 11,32 |
| H_37_ | 1,38 | 1,06 | C_12_ (H_14_, H_15_) | 20,2 | 21,62 |
| H_41_ | 1,22 | 1,05 |  |  |  |
| CMAE^2^ |  | 0,28 |  |  | 2,78 |
| (R^2^)^3^ |  | 0,91 |  |  | 0,97 |
| ^1^ δ_scaled_ = (δ_calc_- intercept)/(slope); ^2^ CMAE = (1)/(N)∑i N\|δscaled-δexp\|;^3^ last-squares linear fitting parameter of the correlation plots between computed (without scaling) and experimental data | | | | | |


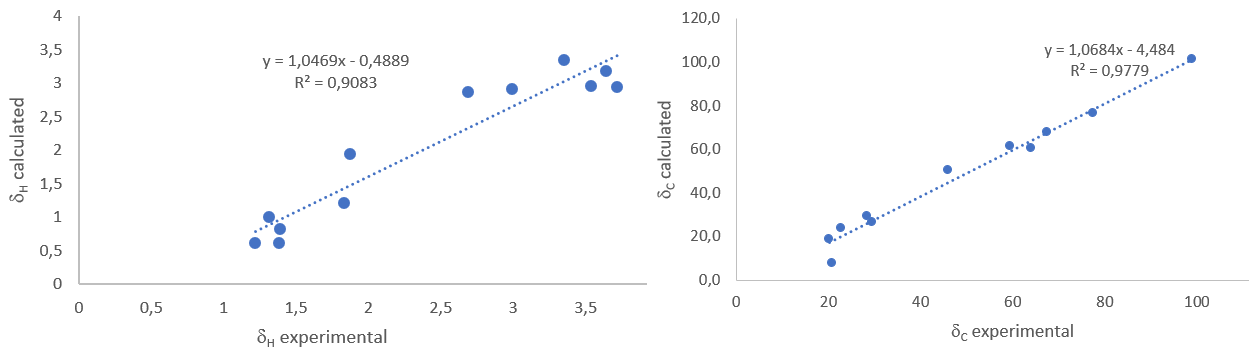


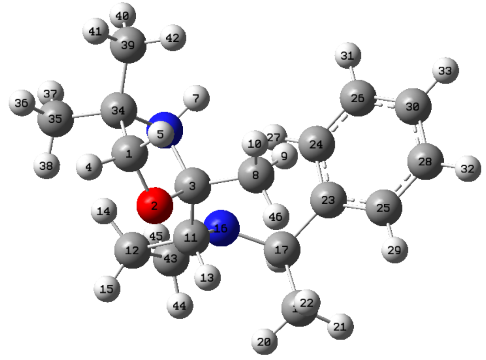

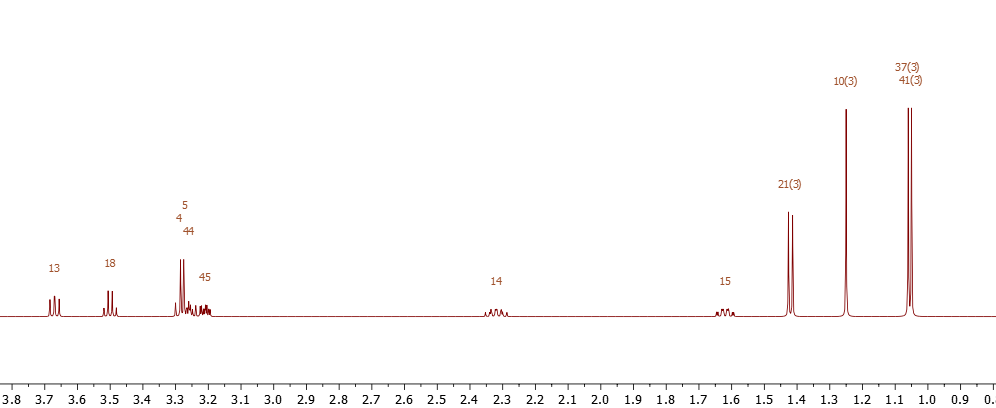


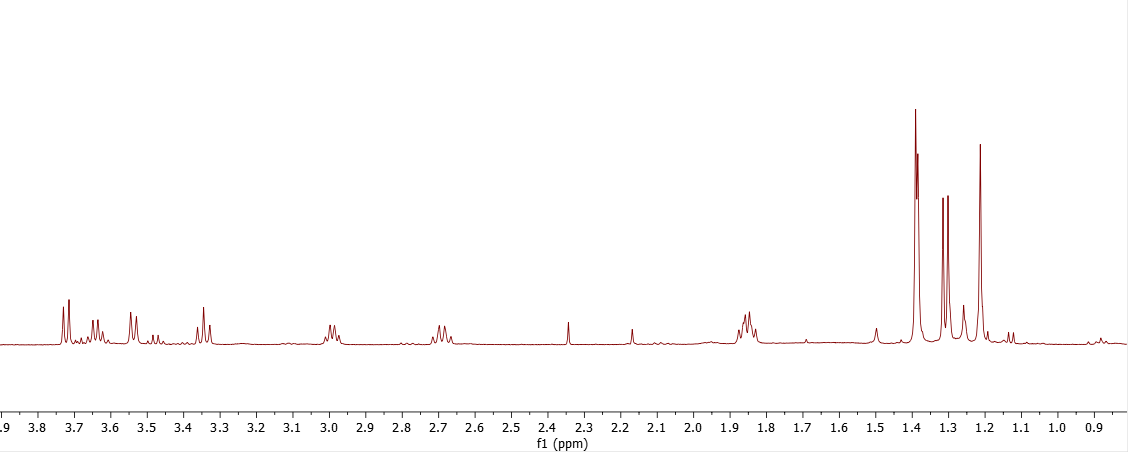


Experimental ^1^H NMR spectra of (*R*,*R*,*R*)-**6c** (bottom), and calculated ^1^H NMR spectra of (*R*,*R*,*S*)-**6c** (top).

***RRR*-6c** diastereoisomer ^1^H- and ^13^C-NMR theoretical prediction.

Conformational Search

%nprocshared=6

%mem=500MW

%chk=OAAMeRRRs.chk

# opt=modredundant rb3lyp/3-21g geom=check

The following ModRedundant input section has been read:

D 43 16 17 19 S 5 30.000

D 2 3 11 12 S 5 30.000


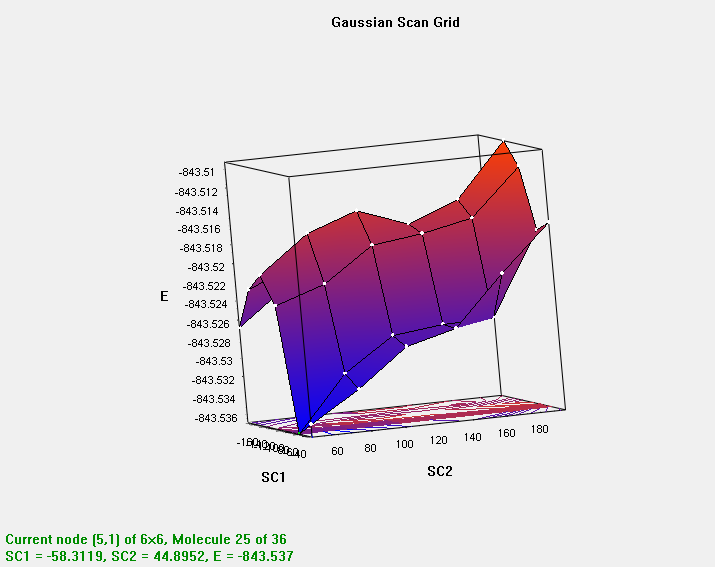


3-D Potential energy surface showing the variation of dihedral angles SC1 D(43,16,17,19) and SC2 D D(2,3,11,12) and their corresponding energies(a. u.)

The 2 better conformational minima, reoptimized in vacuo at rb3lyp/6-311++g(d,p) level, converge towards the same structure. This lowest energy conformer was then considered for the fully unconstrained geometry optimization at SMD/DFT/B3LYP/6-311++G(d,p) level and vibrational analysis.


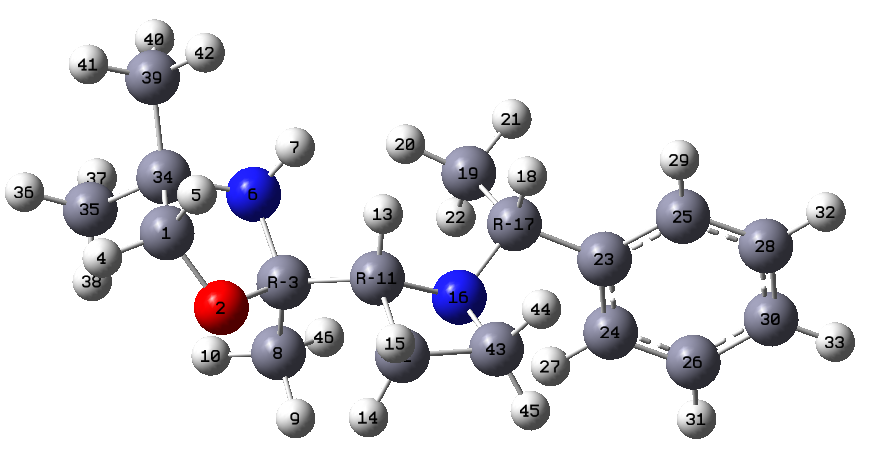


%nprocshared=6

Will use up to 6 processors via shared memory.

%mem=500MW

%chk=OAAMeRRRCDCl3s2 .chk

# opt freq rb3lyp/6-311++g(d,p) scrf=(smd,solvent=chloroform)

Zero-point correction= 0.403864 (Hartree/Particle)

Thermal correction to Energy= 0.423656

Thermal correction to Enthalpy= 0.424600

Thermal correction to Gibbs Free Energy= 0.355650

Sum of electronic and zero-point Energies= -847.999104

Sum of electronic and thermal Energies= -847.979312

Sum of electronic and thermal Enthalpies= -847.978367

Sum of electronic and thermal Free Energies= -848.047318

%nprocshared=6

Will use up to 6 processors via shared memory.

%mem=500MW

%chk=OAAMeRRRCDCl3s2cs.chk

# MPW1PW91/gen scrf=(smd,solvent=chloroform) geom=check guess=read NMR

Calculating GIAO nuclear magnetic shielding tensors.

SCF GIAO Magnetic shielding tensor (ppm):

1 C Isotropic = 108.5475 Anisotropy = 53.9373

XX= 108.1791 YX= 26.3462 ZX= 15.5979

XY= 21.4894 YY= 122.6458 ZY= 2.0937

XZ= 25.5197 YZ= 1.3357 ZZ= 94.8177

Eigenvalues: 75.6814 105.4555 144.5057

2 O Isotropic = 221.1570 Anisotropy = 152.1818

XX= 232.9714 YX= -38.1093 ZX= 64.6761

XY= -27.0236 YY= 293.3998 ZY= -24.3217

XZ= 87.9052 YZ= -11.8037 ZZ= 137.0996

Eigenvalues: 94.9349 245.9245 322.6115

3 C Isotropic = 82.9570 Anisotropy = 54.1347

XX= 89.5427 YX= -14.0297 ZX= 20.2389

XY= -12.8545 YY= 98.8140 ZY= -16.8161

XZ= 23.0634 YZ= -12.5122 ZZ= 60.5142

Eigenvalues: 47.9741 81.8500 119.0467

4 H Isotropic = 27.8144 Anisotropy = 7.6518

XX= 31.6931 YX= -2.4687 ZX= 1.7558

XY= -1.2390 YY= 27.0295 ZY= -0.9347

XZ= 2.1702 YZ= -0.9868 ZZ= 24.7205

Eigenvalues: 24.1179 26.4096 32.9156

5 H Isotropic = 28.2587 Anisotropy = 8.2782

XX= 26.9633 YX= 2.6558 ZX= 3.7289

XY= 2.2397 YY= 25.8697 ZY= 0.0164

XZ= 3.1265 YZ= -0.7194 ZZ= 31.9432

Eigenvalues: 23.1537 27.8449 33.7775

6 N Isotropic = 148.6623 Anisotropy = 70.6565

XX= 172.2480 YX= 23.1152 ZX= 12.2096

XY= 23.4393 YY= 162.6500 ZY= 15.0311

XZ= 11.9380 YZ= 17.3166 ZZ= 111.0890

Eigenvalues: 105.9547 144.2656 195.7666

7 H Isotropic = 29.5903 Anisotropy = 9.4250

XX= 34.7184 YX= 0.6780 ZX= -2.6614

XY= 1.2998 YY= 27.4764 ZY= 5.7441

XZ= -3.4018 YZ= 5.8121 ZZ= 26.5761

Eigenvalues: 20.6288 32.2685 35.8736

8 C Isotropic = 163.4068 Anisotropy = 27.9610

XX= 158.9627 YX= -4.3186 ZX= 5.5949

XY= 0.6765 YY= 154.4329 ZY= 7.8634

XZ= 13.3088 YZ= 5.8197 ZZ= 176.8248

Eigenvalues: 149.7258 158.4472 182.0475

9 H Isotropic = 29.7748 Anisotropy = 7.9196

XX= 27.4715 YX= 2.4837 ZX= -0.2865

XY= 2.3600 YY= 32.0754 ZY= 2.7512

XZ= 0.6126 YZ= 3.9656 ZZ= 29.7774

Eigenvalues: 25.8846 28.3852 35.0545

10 H Isotropic = 30.1053 Anisotropy = 6.9748

XX= 31.8114 YX= 2.2997 ZX= -1.9926

XY= 1.8392 YY= 25.6432 ZY= 1.2597

XZ= -2.7032 YZ= 1.2347 ZZ= 32.8614

Eigenvalues: 24.5643 30.9965 34.7552

11 C Isotropic = 107.5319 Anisotropy = 33.9684

XX= 128.1445 YX= 1.8075 ZX= -2.7706

XY= -15.2146 YY= 90.5707 ZY= -10.3764

XZ= -8.8439 YZ= -1.8501 ZZ= 103.8806

Eigenvalues: 86.4950 105.9233 130.1775

12 C Isotropic = 166.3791 Anisotropy = 24.9994

XX= 174.9535 YX= 11.8311 ZX= -5.9939

XY= 10.2225 YY= 162.1396 ZY= -0.3824

XZ= -1.5724 YZ= -10.5745 ZZ= 162.0441

Eigenvalues: 154.4773 161.6146 183.0453

13 H Isotropic = 28.4296 Anisotropy = 6.0123

XX= 31.9318 YX= 2.0151 ZX= 0.0704

XY= 1.4694 YY= 26.0672 ZY= -1.2238

XZ= -0.5942 YZ= 0.3005 ZZ= 27.2897

Eigenvalues: 25.5085 27.3425 32.4378

14 H Isotropic = 29.4351 Anisotropy = 5.7501

XX= 28.4085 YX= -0.5846 ZX= -2.1582

XY= -1.1817 YY= 32.2445 ZY= 2.6366

XZ= -1.0109 YZ= 0.9845 ZZ= 27.6523

Eigenvalues: 26.2495 28.7873 33.2685

15 H Isotropic = 29.7026 Anisotropy = 9.6435

XX= 27.9909 YX= -1.5004 ZX= 1.7909

XY= -2.5086 YY= 29.8281 ZY= -5.0399

XZ= 1.5502 YZ= -4.3638 ZZ= 31.2888

Eigenvalues: 25.6832 27.2930 36.1316

16 N Isotropic = 181.2476 Anisotropy = 49.2963

XX= 212.5183 YX= -4.4981 ZX= 1.6096

XY= -8.2347 YY= 185.6573 ZY= 0.0195

XZ= -7.0264 YZ= 6.1335 ZZ= 145.5673

Eigenvalues: 145.2584 184.3727 214.1118

17 C Isotropic = 114.5666 Anisotropy = 40.7349

XX= 118.0140 YX= -26.2883 ZX= 0.4831

XY= -20.2510 YY= 116.0944 ZY= 8.3418

XZ= -2.4950 YZ= 8.6097 ZZ= 109.5914

Eigenvalues: 92.0049 109.9716 141.7232

18 H Isotropic = 28.1097 Anisotropy = 6.1879

XX= 28.9090 YX= -1.4725 ZX= -2.6997

XY= -1.8751 YY= 25.6695 ZY= 2.3964

XZ= -1.5523 YZ= 0.5395 ZZ= 29.7506

Eigenvalues: 24.8826 27.2115 32.2350

19 C Isotropic = 162.8038 Anisotropy = 30.2544

XX= 160.1924 YX= 8.6586 ZX= -6.3195

XY= 14.7551 YY= 169.5296 ZY= -7.5279

XZ= -9.3114 YZ= -9.7743 ZZ= 158.6894

Eigenvalues: 151.1844 154.2536 182.9735

20 H Isotropic = 29.9703 Anisotropy = 10.0491

XX= 36.4762 YX= -0.3095 ZX= 1.0955

XY= 2.5907 YY= 29.5239 ZY= 0.3335

XZ= -0.3330 YZ= -0.3303 ZZ= 23.9109

Eigenvalues: 23.8991 29.3422 36.6697

21 H Isotropic = 30.4904 Anisotropy = 8.8546

XX= 29.4956 YX= -2.7718 ZX= -1.2212

XY= -0.5121 YY= 35.8332 ZY= 1.6049

XZ= -1.8698 YZ= 0.2504 ZZ= 26.1424

Eigenvalues: 25.5313 29.5464 36.3935

22 H Isotropic = 30.4111 Anisotropy = 5.7756

XX= 29.1637 YX= -0.7327 ZX= 0.3393

XY= 1.4877 YY= 28.3396 ZY= -2.6660

XZ= -0.1718 YZ= -0.8820 ZZ= 33.7299

Eigenvalues: 27.7062 29.2656 34.2615

23 C Isotropic = 33.5307 Anisotropy = 202.7392

XX= -59.1290 YX= -6.4649 ZX= -6.9165

XY= -4.0091 YY= 145.6368 ZY= -60.2459

XZ= -10.3064 YZ= -59.1140 ZZ= 14.0843

Eigenvalues: -61.0323 -7.0657 168.6902

24 C Isotropic = 53.8885 Anisotropy = 190.5824

XX= -5.5360 YX= 12.5179 ZX= 40.4784

XY= 8.7233 YY= 154.9499 ZY= -65.9938

XZ= 46.4934 YZ= -65.8517 ZZ= 12.2517

Eigenvalues: -54.1537 34.8757 180.9434

25 C Isotropic = 53.1029 Anisotropy = 166.6845

XX= 40.4588 YX= -11.0310 ZX= -23.7288

XY= -19.4352 YY= 138.0800 ZY= -65.8999

XZ= -24.1860 YZ= -71.7566 ZZ= -19.2301

Eigenvalues: -53.3369 48.4197 164.2259

26 C Isotropic = 52.6630 Anisotropy = 187.8869

XX= 32.2659 YX= -10.2041 ZX= -23.1377

XY= -11.1184 YY= 148.0229 ZY= -76.8025

XZ= -24.6877 YZ= -77.9020 ZZ= -22.2997

Eigenvalues: -59.6265 39.6946 177.9209

27 H Isotropic = 23.3812 Anisotropy = 11.4428

XX= 27.1515 YX= 1.4022 ZX= 5.6488

XY= 0.6402 YY= 19.5438 ZY= 1.5501

XZ= 4.6112 YZ= 1.4922 ZZ= 23.4482

Eigenvalues: 18.8591 20.2747 31.0097

28 C Isotropic = 53.7104 Anisotropy = 186.5753

XX= -4.3949 YX= 16.8477 ZX= 46.9148

XY= 15.8778 YY= 154.6714 ZY= -60.6868

XZ= 46.3904 YZ= -64.4689 ZZ= 10.8547

Eigenvalues: -58.0770 41.1143 178.0939

29 H Isotropic = 23.8299 Anisotropy = 9.3337

XX= 28.9942 YX= -1.2676 ZX= -2.8602

XY= -0.7815 YY= 20.1157 ZY= 0.6362

XZ= -2.3564 YZ= 0.9833 ZZ= 22.3799

Eigenvalues: 19.8509 21.5865 30.0524

30 C Isotropic = 54.8500 Anisotropy = 185.1581

XX= -50.4219 YX= -8.1583 ZX= -17.0880

XY= -6.1214 YY= 159.5631 ZY= -48.0593

XZ= -18.6875 YZ= -47.8780 ZZ= 55.4089

Eigenvalues: -54.4903 40.7517 178.2887

31 H Isotropic = 23.6240 Anisotropy = 6.1313

XX= 27.6785 YX= -0.2381 ZX= -0.1621

XY= -0.3780 YY= 20.4366 ZY= 1.0375

XZ= -0.3662 YZ= 1.1082 ZZ= 22.7568

Eigenvalues: 20.0118 23.1485 27.7115

32 H Isotropic = 23.7284 Anisotropy = 5.3790

XX= 26.4032 YX= 0.4307 ZX= 1.5415

XY= 0.3720 YY= 20.8451 ZY= 1.7107

XZ= 1.5340 YZ= 1.3730 ZZ= 23.9370

Eigenvalues: 20.1993 23.6715 27.3144

33 H Isotropic = 23.7893 Anisotropy = 4.4278

XX= 24.4175 YX= -0.3329 ZX= -0.4763

XY= -0.1937 YY= 21.2030 ZY= 2.1651

XZ= -0.6192 YZ= 2.0754 ZZ= 25.7475

Eigenvalues: 20.3669 24.2599 26.7412

34 C Isotropic = 122.8082 Anisotropy = 46.5545

XX= 149.1608 YX= 0.4523 ZX= 19.0235

XY= 0.1542 YY= 119.8609 ZY= -4.4059

XZ= 12.0840 YZ= -17.0790 ZZ= 99.4028

Eigenvalues: 91.1515 123.4285 153.8445

35 C Isotropic = 160.1685 Anisotropy = 41.7006

XX= 164.0887 YX= -4.7136 ZX= -18.2473

XY= -7.2475 YY= 143.2114 ZY= -7.2099

XZ= -19.0434 YZ= -8.0928 ZZ= 173.2054

Eigenvalues: 136.3312 156.2054 187.9689

36 H Isotropic = 30.5359 Anisotropy = 10.9599

XX= 37.2869 YX= 0.5293 ZX= -1.7189

XY= -0.1214 YY= 26.2435 ZY= 0.0122

XZ= -2.9316 YZ= 0.4539 ZZ= 28.0773

Eigenvalues: 26.1856 27.5796 37.8425

37 H Isotropic = 30.5070 Anisotropy = 8.5903

XX= 28.4469 YX= 0.9836 ZX= -1.7229

XY= 0.6266 YY= 31.1030 ZY= -3.6610

XZ= -0.8703 YZ= -5.1101 ZZ= 31.9712

Eigenvalues: 27.0637 28.2235 36.2339

38 H Isotropic = 29.6015 Anisotropy = 7.0577

XX= 29.3145 YX= 1.1520 ZX= -3.2720

XY= 2.0884 YY= 27.6647 ZY= 2.9632

XZ= -1.9688 YZ= 3.9393 ZZ= 31.8252

Eigenvalues: 24.2177 30.2801 34.3066

39 C Isotropic = 161.3735 Anisotropy = 44.9581

XX= 152.5213 YX= 13.8987 ZX= -0.3225

XY= 12.6878 YY= 161.8086 ZY= 25.4992

XZ= -1.8599 YZ= 21.6289 ZZ= 169.7904

Eigenvalues: 134.8064 157.9684 191.3455

40 H Isotropic = 29.9123 Anisotropy = 8.7498

XX= 27.2112 YX= 1.9849 ZX= 0.1611

XY= 1.3870 YY= 35.4121 ZY= -0.6432

XZ= 0.5637 YZ= 0.6101 ZZ= 27.1135

Eigenvalues: 26.6182 27.3731 35.7455

41 H Isotropic = 30.2835 Anisotropy = 10.6965

XX= 34.8874 YX= 2.1597 ZX= 3.3736

XY= 2.8749 YY= 27.5486 ZY= 1.8291

XZ= 3.7047 YZ= 2.0290 ZZ= 28.4143

Eigenvalues: 25.9898 27.4461 37.4145

42 H Isotropic = 30.5956 Anisotropy = 8.7585

XX= 28.8245 YX= 1.4967 ZX= -1.0243

XY= 0.5508 YY= 28.1211 ZY= 3.8857

XZ= -2.1879 YZ= 2.9514 ZZ= 34.8412

Eigenvalues: 25.8359 29.5163 36.4346

43 C Isotropic = 137.0495 Anisotropy = 52.3365

XX= 115.0462 YX= -6.3513 ZX= 1.5581

XY= 3.2053 YY= 162.4087 ZY= -21.2089

XZ= 4.3669 YZ= -16.6501 ZZ= 133.6935

Eigenvalues: 114.5257 124.6823 171.9404

44 H Isotropic = 29.1040 Anisotropy = 5.7914

XX= 30.0274 YX= 0.3067 ZX= -2.8503

XY= -1.7549 YY= 27.6458 ZY= -5.0737

XZ= -1.7042 YZ= -1.7141 ZZ= 29.6388

Eigenvalues: 24.4021 29.9450 32.9649

45 H Isotropic = 28.6134 Anisotropy = 7.4291

XX= 30.4638 YX= 3.7455 ZX= 1.7063

XY= 1.3388 YY= 31.1903 ZY= 1.2093

XZ= 1.5016 YZ= -0.6739 ZZ= 24.1860

Eigenvalues: 23.7827 28.4913 33.5661

46 H Isotropic = 29.7963 Anisotropy = 8.0606

XX= 32.1865 YX= -3.4103 ZX= 1.8460

XY= -2.5131 YY= 26.2276 ZY= -1.3495

XZ= 2.8806 YZ= -1.4267 ZZ= 30.9748

Eigenvalues: 24.9775 29.2414 35.1700

%MEM=500MW

%chk=OAAMeRRRCDCl3s2J.chk

# B3LYP/6-311++G(d,p) guess=read geom=check scrf(smd, solvent=chlorofo

rm) NMR=(spinspin,readatoms)

Total nuclear spin-spin coupling J (Hz):

1 2 3 4 5

1 0.000000D+00

2 0.000000D+00 0.000000D+00

3 0.000000D+00 0.000000D+00 0.000000D+00

4 0.000000D+00 0.000000D+00 0.000000D+00 0.000000D+00

5 0.000000D+00 0.000000D+00 0.000000D+00 -0.704367D+01 0.000000D+00

6 0.000000D+00 0.000000D+00 0.000000D+00 0.000000D+00 0.000000D+00

7 0.000000D+00 0.000000D+00 0.000000D+00 0.000000D+00 0.000000D+00

8 0.000000D+00 0.000000D+00 0.000000D+00 0.000000D+00 0.000000D+00

9 0.000000D+00 0.000000D+00 0.000000D+00 0.000000D+00 0.000000D+00

10 0.000000D+00 0.000000D+00 0.000000D+00 0.000000D+00 0.000000D+00

11 0.000000D+00 0.000000D+00 0.000000D+00 0.000000D+00 0.000000D+00

12 0.000000D+00 0.000000D+00 0.000000D+00 0.000000D+00 0.000000D+00

13 0.000000D+00 0.000000D+00 0.000000D+00 -0.118992D+00 0.283339D+00

14 0.000000D+00 0.000000D+00 0.000000D+00 -0.823502D-01 -0.658310D-01

15 0.000000D+00 0.000000D+00 0.000000D+00 -0.783699D-01 0.151462D+00

16 0.000000D+00 0.000000D+00 0.000000D+00 0.000000D+00 0.000000D+00

17 0.000000D+00 0.000000D+00 0.000000D+00 0.000000D+00 0.000000D+00

18 0.000000D+00 0.000000D+00 0.000000D+00 -0.102538D+00 0.360223D-01

19 0.000000D+00 0.000000D+00 0.000000D+00 0.000000D+00 0.000000D+00

20 0.000000D+00 0.000000D+00 0.000000D+00 0.000000D+00 0.000000D+00

21 0.000000D+00 0.000000D+00 0.000000D+00 -0.941582D-01 -0.625507D-01

22 0.000000D+00 0.000000D+00 0.000000D+00 -0.114771D+00 -0.874270D-01

23 0.000000D+00 0.000000D+00 0.000000D+00 0.000000D+00 0.000000D+00

24 0.000000D+00 0.000000D+00 0.000000D+00 0.000000D+00 0.000000D+00

25 0.000000D+00 0.000000D+00 0.000000D+00 0.000000D+00 0.000000D+00

26 0.000000D+00 0.000000D+00 0.000000D+00 0.000000D+00 0.000000D+00

27 0.000000D+00 0.000000D+00 0.000000D+00 0.000000D+00 0.000000D+00

28 0.000000D+00 0.000000D+00 0.000000D+00 0.000000D+00 0.000000D+00

29 0.000000D+00 0.000000D+00 0.000000D+00 0.000000D+00 0.000000D+00

30 0.000000D+00 0.000000D+00 0.000000D+00 0.000000D+00 0.000000D+00

31 0.000000D+00 0.000000D+00 0.000000D+00 0.000000D+00 0.000000D+00

32 0.000000D+00 0.000000D+00 0.000000D+00 0.000000D+00 0.000000D+00

33 0.000000D+00 0.000000D+00 0.000000D+00 0.000000D+00 0.000000D+00

34 0.000000D+00 0.000000D+00 0.000000D+00 0.000000D+00 0.000000D+00

35 0.000000D+00 0.000000D+00 0.000000D+00 0.000000D+00 0.000000D+00

36 0.000000D+00 0.000000D+00 0.000000D+00 0.000000D+00 0.000000D+00

37 0.000000D+00 0.000000D+00 0.000000D+00 0.000000D+00 0.000000D+00

38 0.000000D+00 0.000000D+00 0.000000D+00 0.000000D+00 0.000000D+00

39 0.000000D+00 0.000000D+00 0.000000D+00 0.000000D+00 0.000000D+00

40 0.000000D+00 0.000000D+00 0.000000D+00 0.000000D+00 0.000000D+00

41 0.000000D+00 0.000000D+00 0.000000D+00 0.000000D+00 0.000000D+00

42 0.000000D+00 0.000000D+00 0.000000D+00 0.000000D+00 0.000000D+00

43 0.000000D+00 0.000000D+00 0.000000D+00 0.000000D+00 0.000000D+00

44 0.000000D+00 0.000000D+00 0.000000D+00 -0.115014D+00 -0.191603D-01

45 0.000000D+00 0.000000D+00 0.000000D+00 -0.128701D+00 -0.100353D+00

46 0.000000D+00 0.000000D+00 0.000000D+00 0.000000D+00 0.000000D+00

6 7 8 9 10

6 0.000000D+00

7 0.000000D+00 0.000000D+00

8 0.000000D+00 0.000000D+00 0.000000D+00

9 0.000000D+00 0.000000D+00 0.000000D+00 0.000000D+00

10 0.000000D+00 0.000000D+00 0.000000D+00 0.000000D+00 0.000000D+00

11 0.000000D+00 0.000000D+00 0.000000D+00 0.000000D+00 0.000000D+00

12 0.000000D+00 0.000000D+00 0.000000D+00 0.000000D+00 0.000000D+00

13 0.000000D+00 0.000000D+00 0.000000D+00 0.000000D+00 0.000000D+00

14 0.000000D+00 0.000000D+00 0.000000D+00 0.000000D+00 0.000000D+00

15 0.000000D+00 0.000000D+00 0.000000D+00 0.000000D+00 0.000000D+00

16 0.000000D+00 0.000000D+00 0.000000D+00 0.000000D+00 0.000000D+00

17 0.000000D+00 0.000000D+00 0.000000D+00 0.000000D+00 0.000000D+00

18 0.000000D+00 0.000000D+00 0.000000D+00 0.000000D+00 0.000000D+00

19 0.000000D+00 0.000000D+00 0.000000D+00 0.000000D+00 0.000000D+00

20 0.000000D+00 0.000000D+00 0.000000D+00 0.000000D+00 0.000000D+00

21 0.000000D+00 0.000000D+00 0.000000D+00 0.000000D+00 0.000000D+00

22 0.000000D+00 0.000000D+00 0.000000D+00 0.000000D+00 0.000000D+00

23 0.000000D+00 0.000000D+00 0.000000D+00 0.000000D+00 0.000000D+00

24 0.000000D+00 0.000000D+00 0.000000D+00 0.000000D+00 0.000000D+00

25 0.000000D+00 0.000000D+00 0.000000D+00 0.000000D+00 0.000000D+00

26 0.000000D+00 0.000000D+00 0.000000D+00 0.000000D+00 0.000000D+00

27 0.000000D+00 0.000000D+00 0.000000D+00 0.000000D+00 0.000000D+00

28 0.000000D+00 0.000000D+00 0.000000D+00 0.000000D+00 0.000000D+00

29 0.000000D+00 0.000000D+00 0.000000D+00 0.000000D+00 0.000000D+00

30 0.000000D+00 0.000000D+00 0.000000D+00 0.000000D+00 0.000000D+00

31 0.000000D+00 0.000000D+00 0.000000D+00 0.000000D+00 0.000000D+00

32 0.000000D+00 0.000000D+00 0.000000D+00 0.000000D+00 0.000000D+00

33 0.000000D+00 0.000000D+00 0.000000D+00 0.000000D+00 0.000000D+00

34 0.000000D+00 0.000000D+00 0.000000D+00 0.000000D+00 0.000000D+00

35 0.000000D+00 0.000000D+00 0.000000D+00 0.000000D+00 0.000000D+00

36 0.000000D+00 0.000000D+00 0.000000D+00 0.000000D+00 0.000000D+00

37 0.000000D+00 0.000000D+00 0.000000D+00 0.000000D+00 0.000000D+00

38 0.000000D+00 0.000000D+00 0.000000D+00 0.000000D+00 0.000000D+00

39 0.000000D+00 0.000000D+00 0.000000D+00 0.000000D+00 0.000000D+00

40 0.000000D+00 0.000000D+00 0.000000D+00 0.000000D+00 0.000000D+00

41 0.000000D+00 0.000000D+00 0.000000D+00 0.000000D+00 0.000000D+00

42 0.000000D+00 0.000000D+00 0.000000D+00 0.000000D+00 0.000000D+00

43 0.000000D+00 0.000000D+00 0.000000D+00 0.000000D+00 0.000000D+00

44 0.000000D+00 0.000000D+00 0.000000D+00 0.000000D+00 0.000000D+00

45 0.000000D+00 0.000000D+00 0.000000D+00 0.000000D+00 0.000000D+00

46 0.000000D+00 0.000000D+00 0.000000D+00 0.000000D+00 0.000000D+00

11 12 13 14 15

11 0.000000D+00

12 0.000000D+00 0.000000D+00

13 0.000000D+00 0.000000D+00 0.000000D+00

14 0.000000D+00 0.000000D+00 0.708602D+01 0.000000D+00

15 0.000000D+00 0.000000D+00 0.766637D+01 -0.974367D+01 0.000000D+00

16 0.000000D+00 0.000000D+00 0.000000D+00 0.000000D+00 0.000000D+00

17 0.000000D+00 0.000000D+00 0.000000D+00 0.000000D+00 0.000000D+00

18 0.000000D+00 0.000000D+00 0.547861D+00 0.333881D+00 -0.246607D+00

19 0.000000D+00 0.000000D+00 0.000000D+00 0.000000D+00 0.000000D+00

20 0.000000D+00 0.000000D+00 0.000000D+00 0.000000D+00 0.000000D+00

21 0.000000D+00 0.000000D+00 -0.846049D-01 -0.179425D+00 0.115888D+00

22 0.000000D+00 0.000000D+00 -0.100910D+00 -0.893873D-01 -0.832209D-01

23 0.000000D+00 0.000000D+00 0.000000D+00 0.000000D+00 0.000000D+00

24 0.000000D+00 0.000000D+00 0.000000D+00 0.000000D+00 0.000000D+00

25 0.000000D+00 0.000000D+00 0.000000D+00 0.000000D+00 0.000000D+00

26 0.000000D+00 0.000000D+00 0.000000D+00 0.000000D+00 0.000000D+00

27 0.000000D+00 0.000000D+00 0.000000D+00 0.000000D+00 0.000000D+00

28 0.000000D+00 0.000000D+00 0.000000D+00 0.000000D+00 0.000000D+00

29 0.000000D+00 0.000000D+00 0.000000D+00 0.000000D+00 0.000000D+00

30 0.000000D+00 0.000000D+00 0.000000D+00 0.000000D+00 0.000000D+00

31 0.000000D+00 0.000000D+00 0.000000D+00 0.000000D+00 0.000000D+00

32 0.000000D+00 0.000000D+00 0.000000D+00 0.000000D+00 0.000000D+00

33 0.000000D+00 0.000000D+00 0.000000D+00 0.000000D+00 0.000000D+00

34 0.000000D+00 0.000000D+00 0.000000D+00 0.000000D+00 0.000000D+00

35 0.000000D+00 0.000000D+00 0.000000D+00 0.000000D+00 0.000000D+00

36 0.000000D+00 0.000000D+00 0.000000D+00 0.000000D+00 0.000000D+00

37 0.000000D+00 0.000000D+00 0.000000D+00 0.000000D+00 0.000000D+00

38 0.000000D+00 0.000000D+00 0.000000D+00 0.000000D+00 0.000000D+00

39 0.000000D+00 0.000000D+00 0.000000D+00 0.000000D+00 0.000000D+00

40 0.000000D+00 0.000000D+00 0.000000D+00 0.000000D+00 0.000000D+00

41 0.000000D+00 0.000000D+00 0.000000D+00 0.000000D+00 0.000000D+00

42 0.000000D+00 0.000000D+00 0.000000D+00 0.000000D+00 0.000000D+00

43 0.000000D+00 0.000000D+00 0.000000D+00 0.000000D+00 0.000000D+00

44 0.000000D+00 0.000000D+00 -0.400219D+00 0.776728D+01 0.770271D+01

45 0.000000D+00 0.000000D+00 -0.107504D+01 0.795061D+01 0.166119D+01

46 0.000000D+00 0.000000D+00 0.000000D+00 0.000000D+00 0.000000D+00

16 17 18 19 20

16 0.000000D+00

17 0.000000D+00 0.000000D+00

18 0.000000D+00 0.000000D+00 0.000000D+00

19 0.000000D+00 0.000000D+00 0.000000D+00 0.000000D+00

20 0.000000D+00 0.000000D+00 0.000000D+00 0.000000D+00 0.000000D+00

21 0.000000D+00 0.000000D+00 0.251086D+01 0.000000D+00 0.000000D+00

22 0.000000D+00 0.000000D+00 0.112647D+02 0.000000D+00 0.000000D+00

23 0.000000D+00 0.000000D+00 0.000000D+00 0.000000D+00 0.000000D+00

24 0.000000D+00 0.000000D+00 0.000000D+00 0.000000D+00 0.000000D+00

25 0.000000D+00 0.000000D+00 0.000000D+00 0.000000D+00 0.000000D+00

26 0.000000D+00 0.000000D+00 0.000000D+00 0.000000D+00 0.000000D+00

27 0.000000D+00 0.000000D+00 0.000000D+00 0.000000D+00 0.000000D+00

28 0.000000D+00 0.000000D+00 0.000000D+00 0.000000D+00 0.000000D+00

29 0.000000D+00 0.000000D+00 0.000000D+00 0.000000D+00 0.000000D+00

30 0.000000D+00 0.000000D+00 0.000000D+00 0.000000D+00 0.000000D+00

31 0.000000D+00 0.000000D+00 0.000000D+00 0.000000D+00 0.000000D+00

32 0.000000D+00 0.000000D+00 0.000000D+00 0.000000D+00 0.000000D+00

33 0.000000D+00 0.000000D+00 0.000000D+00 0.000000D+00 0.000000D+00

34 0.000000D+00 0.000000D+00 0.000000D+00 0.000000D+00 0.000000D+00

35 0.000000D+00 0.000000D+00 0.000000D+00 0.000000D+00 0.000000D+00

36 0.000000D+00 0.000000D+00 0.000000D+00 0.000000D+00 0.000000D+00

37 0.000000D+00 0.000000D+00 0.000000D+00 0.000000D+00 0.000000D+00

38 0.000000D+00 0.000000D+00 0.000000D+00 0.000000D+00 0.000000D+00

39 0.000000D+00 0.000000D+00 0.000000D+00 0.000000D+00 0.000000D+00

40 0.000000D+00 0.000000D+00 0.000000D+00 0.000000D+00 0.000000D+00

41 0.000000D+00 0.000000D+00 0.000000D+00 0.000000D+00 0.000000D+00

42 0.000000D+00 0.000000D+00 0.000000D+00 0.000000D+00 0.000000D+00

43 0.000000D+00 0.000000D+00 0.000000D+00 0.000000D+00 0.000000D+00

44 0.000000D+00 0.000000D+00 0.434957D+00 0.000000D+00 0.000000D+00

45 0.000000D+00 0.000000D+00 -0.124901D+00 0.000000D+00 0.000000D+00

46 0.000000D+00 0.000000D+00 0.000000D+00 0.000000D+00 0.000000D+00

21 22 23 24 25

21 0.000000D+00

22 -0.119640D+02 0.000000D+00

23 0.000000D+00 0.000000D+00 0.000000D+00

24 0.000000D+00 0.000000D+00 0.000000D+00 0.000000D+00

25 0.000000D+00 0.000000D+00 0.000000D+00 0.000000D+00 0.000000D+00

26 0.000000D+00 0.000000D+00 0.000000D+00 0.000000D+00 0.000000D+00

27 0.000000D+00 0.000000D+00 0.000000D+00 0.000000D+00 0.000000D+00

28 0.000000D+00 0.000000D+00 0.000000D+00 0.000000D+00 0.000000D+00

29 0.000000D+00 0.000000D+00 0.000000D+00 0.000000D+00 0.000000D+00

30 0.000000D+00 0.000000D+00 0.000000D+00 0.000000D+00 0.000000D+00

31 0.000000D+00 0.000000D+00 0.000000D+00 0.000000D+00 0.000000D+00

32 0.000000D+00 0.000000D+00 0.000000D+00 0.000000D+00 0.000000D+00

33 0.000000D+00 0.000000D+00 0.000000D+00 0.000000D+00 0.000000D+00

34 0.000000D+00 0.000000D+00 0.000000D+00 0.000000D+00 0.000000D+00

35 0.000000D+00 0.000000D+00 0.000000D+00 0.000000D+00 0.000000D+00

36 0.000000D+00 0.000000D+00 0.000000D+00 0.000000D+00 0.000000D+00

37 0.000000D+00 0.000000D+00 0.000000D+00 0.000000D+00 0.000000D+00

38 0.000000D+00 0.000000D+00 0.000000D+00 0.000000D+00 0.000000D+00

39 0.000000D+00 0.000000D+00 0.000000D+00 0.000000D+00 0.000000D+00

40 0.000000D+00 0.000000D+00 0.000000D+00 0.000000D+00 0.000000D+00

41 0.000000D+00 0.000000D+00 0.000000D+00 0.000000D+00 0.000000D+00

42 0.000000D+00 0.000000D+00 0.000000D+00 0.000000D+00 0.000000D+00

43 0.000000D+00 0.000000D+00 0.000000D+00 0.000000D+00 0.000000D+00

44 -0.238705D+00 -0.123361D+00 0.000000D+00 0.000000D+00 0.000000D+00

45 -0.301846D+00 -0.797342D-01 0.000000D+00 0.000000D+00 0.000000D+00

46 0.000000D+00 0.000000D+00 0.000000D+00 0.000000D+00 0.000000D+00

26 27 28 29 30

26 0.000000D+00

27 0.000000D+00 0.000000D+00

28 0.000000D+00 0.000000D+00 0.000000D+00

29 0.000000D+00 0.000000D+00 0.000000D+00 0.000000D+00

30 0.000000D+00 0.000000D+00 0.000000D+00 0.000000D+00 0.000000D+00

31 0.000000D+00 0.000000D+00 0.000000D+00 0.000000D+00 0.000000D+00

32 0.000000D+00 0.000000D+00 0.000000D+00 0.000000D+00 0.000000D+00

33 0.000000D+00 0.000000D+00 0.000000D+00 0.000000D+00 0.000000D+00

34 0.000000D+00 0.000000D+00 0.000000D+00 0.000000D+00 0.000000D+00

35 0.000000D+00 0.000000D+00 0.000000D+00 0.000000D+00 0.000000D+00

36 0.000000D+00 0.000000D+00 0.000000D+00 0.000000D+00 0.000000D+00

37 0.000000D+00 0.000000D+00 0.000000D+00 0.000000D+00 0.000000D+00

38 0.000000D+00 0.000000D+00 0.000000D+00 0.000000D+00 0.000000D+00

39 0.000000D+00 0.000000D+00 0.000000D+00 0.000000D+00 0.000000D+00

40 0.000000D+00 0.000000D+00 0.000000D+00 0.000000D+00 0.000000D+00

41 0.000000D+00 0.000000D+00 0.000000D+00 0.000000D+00 0.000000D+00

42 0.000000D+00 0.000000D+00 0.000000D+00 0.000000D+00 0.000000D+00

43 0.000000D+00 0.000000D+00 0.000000D+00 0.000000D+00 0.000000D+00

44 0.000000D+00 0.000000D+00 0.000000D+00 0.000000D+00 0.000000D+00

45 0.000000D+00 0.000000D+00 0.000000D+00 0.000000D+00 0.000000D+00

46 0.000000D+00 0.000000D+00 0.000000D+00 0.000000D+00 0.000000D+00

31 32 33 34 35

31 0.000000D+00

32 0.000000D+00 0.000000D+00

33 0.000000D+00 0.000000D+00 0.000000D+00

34 0.000000D+00 0.000000D+00 0.000000D+00 0.000000D+00

35 0.000000D+00 0.000000D+00 0.000000D+00 0.000000D+00 0.000000D+00

36 0.000000D+00 0.000000D+00 0.000000D+00 0.000000D+00 0.000000D+00

37 0.000000D+00 0.000000D+00 0.000000D+00 0.000000D+00 0.000000D+00

38 0.000000D+00 0.000000D+00 0.000000D+00 0.000000D+00 0.000000D+00

39 0.000000D+00 0.000000D+00 0.000000D+00 0.000000D+00 0.000000D+00

40 0.000000D+00 0.000000D+00 0.000000D+00 0.000000D+00 0.000000D+00

41 0.000000D+00 0.000000D+00 0.000000D+00 0.000000D+00 0.000000D+00

42 0.000000D+00 0.000000D+00 0.000000D+00 0.000000D+00 0.000000D+00

43 0.000000D+00 0.000000D+00 0.000000D+00 0.000000D+00 0.000000D+00

44 0.000000D+00 0.000000D+00 0.000000D+00 0.000000D+00 0.000000D+00

45 0.000000D+00 0.000000D+00 0.000000D+00 0.000000D+00 0.000000D+00

46 0.000000D+00 0.000000D+00 0.000000D+00 0.000000D+00 0.000000D+00

36 37 38 39 40

36 0.000000D+00

37 0.000000D+00 0.000000D+00

38 0.000000D+00 0.000000D+00 0.000000D+00

39 0.000000D+00 0.000000D+00 0.000000D+00 0.000000D+00

40 0.000000D+00 0.000000D+00 0.000000D+00 0.000000D+00 0.000000D+00

41 0.000000D+00 0.000000D+00 0.000000D+00 0.000000D+00 0.000000D+00

42 0.000000D+00 0.000000D+00 0.000000D+00 0.000000D+00 0.000000D+00

43 0.000000D+00 0.000000D+00 0.000000D+00 0.000000D+00 0.000000D+00

44 0.000000D+00 0.000000D+00 0.000000D+00 0.000000D+00 0.000000D+00

45 0.000000D+00 0.000000D+00 0.000000D+00 0.000000D+00 0.000000D+00

46 0.000000D+00 0.000000D+00 0.000000D+00 0.000000D+00 0.000000D+00

41 42 43 44 45

41 0.000000D+00

42 0.000000D+00 0.000000D+00

43 0.000000D+00 0.000000D+00 0.000000D+00

44 0.000000D+00 0.000000D+00 0.000000D+00 0.000000D+00

45 0.000000D+00 0.000000D+00 0.000000D+00 -0.607556D+01 0.000000D+00

46 0.000000D+00 0.000000D+00 0.000000D+00 0.000000D+00 0.000000D+00

46

46 0.000000D+00

Table 2. Experimental and calculated ^1^H and^13^C NMR data for the ***RRR*-6c** diastereoisomer with his HSQC correlations

| **Label H** | **δ_H_ exp** | **δ_H_ scaled**^1^ | **Label C (HSQC_corr)_** | **δ_C_ exp** | **δ_C_ scaled**^1^ |
| --- | --- | --- | --- | --- | --- |
| H_13_ | 3,35 | 3,26 | C_3_ | 98,98 | 96,55 |
| H_18_ | 3,64 | 3,61 | C_1_ (H_4,_ H_5_) | 77,47 | 73,13 |
| H_4_ | 3,72 | 3,93 | C_11_ | 67,63 | 74,06 |
| H_5_ | 3,54 | 3,45 | C_17_ (H_18_) | 64,12 | 67,63 |
| H_44_ | 2,99 | 3,05 | C_34_ | 59,63 | 60,09 |
| H_45_ | 2,69 | 2,52 | C_43_ (H_44,_ H_45_) | 46,04 | 47,06 |
| H_14_ | 1,87 | 2,00 | C_39_ (H_37_) | 29,66 | 25,91 |
| H_15_ | 1,83 | 1,89 | C_35_ (H_41_) | 28,4 | 24,80 |
| H_21_(Me) | 1,31 | 1,23 | C_8_ (H_10_) | 22,92 | 22,94 |
| H_10_(Me) | 1,39 | 1,44 | C_19_ (H_21_) | 20,83 | 23,50 |
| H_37_(Me) | 1,38 | 1,31 | C_12_ (H_14,_ H_15_) | 20,2 | 20,22 |
| H_41_(Me) | 1,22 | 1,25 |  |  |  |
| CMAE^2^ |  | 0,09 |  |  | 2,22 |
| (R^2^) ^3^ |  | 0,99 |  |  | 0,98 |
| ^1^ δscaled = (δcalc- intercept)/(slope);^2^ CMAE = (1)/(N)∑i N\|δscaled-δexp\|;^3^ last-squares linear fitting parameter of the correlation plots between computed (without scaling) and experimental data | | | | | |


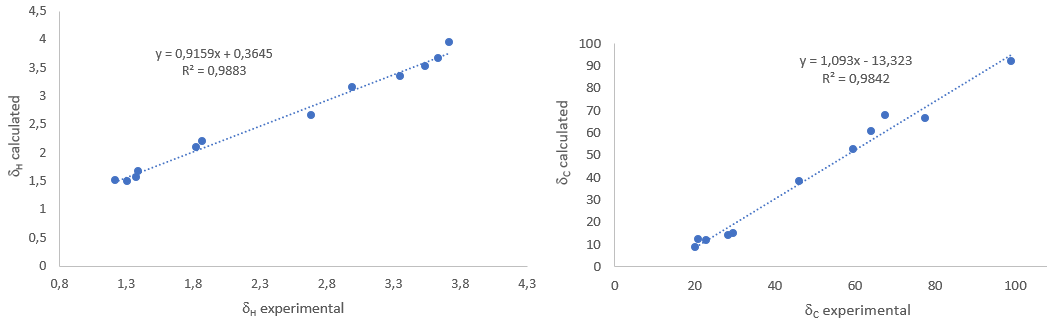


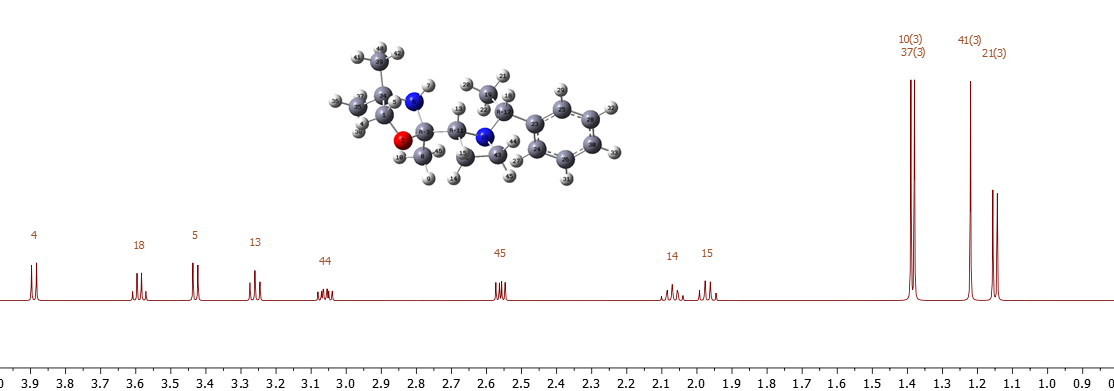


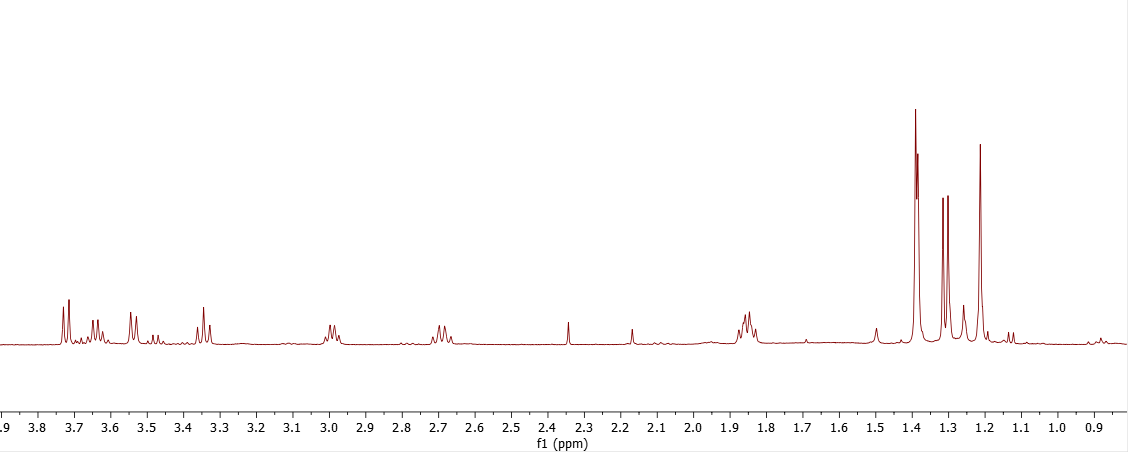


Experimental ^1^H NMR spectra of (*R*,*R*,*R*)-**6c** (bottom), and calculated ^1^H NMR spectra of (*R*,*R*,*R*)-**6c** (top).
